# Both Benzannulation and Heteroatom-Controlled Photophysical Properties in Donor–π–Acceptor Ionic Dyes: A Combined Experimental and Theoretical Study

**DOI:** 10.3390/ma18204676

**Published:** 2025-10-12

**Authors:** Przemysław Krawczyk, Beata Jędrzejewska

**Affiliations:** 1 Faculty of Pharmacy, Collegium Medicum, Nicolaus Copernicus University, Kurpińskiego 5, 85-096 Bydgoszcz, Poland; przemekk@cm.umk.pl; 2 Faculty of Chemical Technology and Engineering, Bydgoszcz University of Science and Technology, Seminaryjna 3, 85-326 Bydgoszcz, Poland

**Keywords:** donor–π–acceptor ionic dyes, spectroscopic properties, benzannulation, heteroatoms, quantum chemical calculations

## Abstract

Donor–π–acceptor (D–π–A) dyes have garnered significant attention due to their unique optical properties and potential applications in various fields, including optoelectronics, chemical sensing and bioimaging. This study presents the design, synthesis, and comprehensive photophysical investigation of a series of ionic dyes incorporating five- and six-membered heterocyclic rings as electron-donating and electron-withdrawing units, respectively. The influence of the dye structure, i.e., (a) the systematically varied heteroatom (NMe, S and O) in donor moiety, (b) benzannulation of the acceptor part and (c) position of the donor vs. acceptor, on the photophysical properties was evaluated by steady-state and time-resolved spectroscopy across solvents of varying polarity. To probe solvatochromic behavior, the Reichardt parameters and the Catalán four-parameter scale, including polarizability (SP), dipolarity (SdP), acidity (SA) and basicity (SB) parameters, were applied. Emission dynamics were further analyzed through time-resolved fluorescence spectroscopy employing multi-exponential decay models to accurately describe fluorescence lifetimes. Time-dependent density functional theory (TDDFT) calculations supported the experimental findings by elucidating electronic structures, charge-transfer character, and dipole moments in the ground and excited states. The experimental results show the introduction of O or S instead of NMe causes substantial hypsochromic shifts in the absorption and emission bands. Benzannulation enhances the photoinduced charge transfer and causes red-shifted absorption spectra to be obtained without deteriorating the emission properties. Hence, by introducing an appropriate modification, it is possible to design materials with tunable photophysical properties for practical applications, e.g., in opto-electronics or sensing.

## 1. Introduction

Dyes with a donor–π–acceptor (D–π–A) structure constitute a class of organic compounds of significant importance in the fields of molecular photonics, organic electronics and solar energy conversion [1,2,3,4]. Their characteristic molecular architecture, comprising a conjugated π-electron system connecting electron donor (D) and acceptor (A), enables efficient charge transfer in the excited state, which translates into unique photophysical properties such as intense absorption in visible light, high fluorescence efficiency, and susceptibility to modulation of optical properties in response to external factors [5,6,7].

One effective way to modify the optical properties of D–π–A dyes is benzannulation, i.e., the introduction of additional benzene rings into the molecular core. This structural modification leads to the expansion of the conjugated system and affects both the stability and nature of electronic states, particularly the HOMO–LUMO levels. It has been shown that benzannulation can lead to a bathochromic shift in absorption bands, increased fluorescence, and improved chemical stability of the dyes [8,9]. Importantly, this effect may be strongly dependent on the site and method of benzannulation and the presence of heteroatoms in the molecular structure. In most cases, a bathochromic spectral shift is observed upon the introduction of an additional aromatic ring into the parent structure, which results from a reduction in the separation between the HOMO and LUMO energies due to destabilization of the highest occupied molecular orbital (HOMO) and stabilization of the lowest unoccupied molecular orbital (LUMO) [10]. A classic example is the benzannulation of benzene, leading to the formation of naphthalene and anthracene [11]. However, examples of small molecules that exhibit the opposite effect, i.e., a blue shift in the absorption band, after benzannulation can also be found in the literature [12,13].

Controlling photophysical properties using heteroatoms such as nitrogen, sulfur, or oxygen opens new possibilities for the design of D–π–A systems. These heteroatoms can influence the electron density, polarity, and orbital character of excited states, and thus modulate photophysical properties [14,15].

A particularly interesting group is ionic dyes derived from methylpyridine and methylquinoline, which utilize five-membered heteroaromatic systems such as furan, thiophene, and methylpyrrole as donor moieties. The structures of thiophene and furan are very similar to those of pyrrole, except that NH is replaced by S and O, respectively. Consequently, the heteroatom in each has one lone electron pair that is part of an aromatic sextet, as in pyrrole, but it also has a second electron pair that is not part of the aromatic system and is in a sp^2^ hybrid orbital in the plane of the ring. Mesomeric forms analogous to pyrrole can be written for each of them. In the case of pyrrole, resonance leads to the fixation of partial negative charges on carbon and partial positive charges on nitrogen, and electron transfer occurs from nitrogen towards carbons. Thus, electron donors containing heteroatoms (O, S, N) differ not only in electronegativity and ability to couple to the π system but also influence the planarization of the molecule and intermolecular interactions, which can significantly modulate both the absorption and emission of light. For example, thiophene, due to its higher polarity and ability to stabilize the excited state, often results in a bathochromic spectral shift, while furan, being more electrophilic, promotes efficient charge transfer while maintaining high fluorescence. Methylpyrrole combines the features of both systems, simultaneously increasing the molecule’s planarity and improving its emission properties in polar environments [16,17,18,19].

Understanding the mechanisms responsible for these phenomena requires an interdisciplinary approach, combining experimental spectroscopy methods (e.g., UV-Vis, fluorescence, TCSPC) with quantum calculations (e.g., DFT, TDDFT), which allows for precise assignment of observed electronic transitions and insight into the nature of excited states.

In recent years, there has been growing interest in using integrated experimental and theoretical studies for the rational design of D–π–A dyes with desired properties. In particular, these studies focus on understanding the influence of structural modification of the core (including benzannulation) and subtle chemical effects induced by heteroatoms, which may lead to the development of new functional materials with optimized optoelectronic parameters [20,21,22].

The aim of this study was to characterize the photophysical properties of twelve ionic compounds with the D–π–A structure in the context of their structure modifications, i.e., (a) systematically changing heteroatom (NMe, S, and O) in the donor moiety, (b) benzannulation of the acceptor moiety, and (c) position of the acceptor relative to the donor. The experimental results were supplemented with theoretical calculations.

We suspect that structural modification of ionic D–π–A dyes by benzannulation of the acceptor moiety (methylpyridine vs. methylquinoline) and the use of various heterocyclic electron donors (furan, thiophene, methylpyrrole) significantly influence the photophysical properties of these compounds, in particular, the position and intensity of absorption and emission bands, the nature of electronic transitions, and the efficiency of charge transfer. It is assumed that the presence of heteroatoms in electron donors and the elongation of the conjugated system via benzannulation will have a synergistic effect, modulating the optical and electronic properties of these systems, which can be captured and verified using integrated spectroscopic and computational methods.

## 2. Materials and Methods

### 2.1. Materials and Instruments

**Figure 1:**
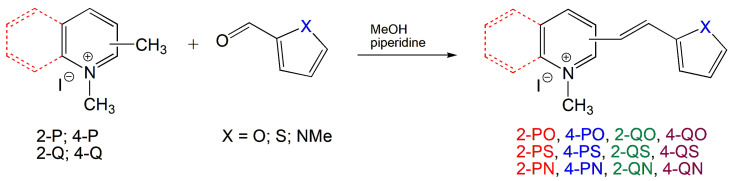
Scheme of the synthesis of push-pull dyes containing a five-membered heterocycle as an electron-donor.

The preparation of ionic D–π–A dyes containing a five-membered heteroaromatic systems such as furan, thiophene, and methylpyrrole as an electron donor was based on the methodology described in the literature [23,24,25,26,27]. The synthetic route is shown in Figure 1, and the method and the analysis of ^1^H and ^13^C NMR, IR spectra and HPLC chromatograms are presented in the ESI file.

All reagents and solvents were commercially available from Merck Life Science Sp. z o.o., Poznań, Poland, an affiliate of Merck KGaA, Darmstadt, Germany and were used without further purification. Melting points were measured on a Boëthius apparatus (type PHMK 05, VEB Analytik, Jena, Germany) without correction. ^1^H and ^13^C NMR spectra were recorded on a on a Bruker Ascent^TM^ 400 NMR spectrometer (Bruker, Billerica, MA, USA) in DMSO-*d*_6_ using tetramethylsilane (TMS) as the internal standard. FT-IR spectra were recorded on a Bruker spectrometer Vector 22 (Bruker, Billerica, MA, USA) using a powder sample on a KBr plate. The HPLC analysis was conducted by a Waters Breeze 2 HPLC (Waters, Milford, MA, USA) system equipped with UV-Vis detector and a Symmetry C18 column (3.5 μm, 4.6 × 75 mm). Separation was conducted under isocratic conditions with 1.0 mL/min flow rate at room temperature, 10 μL injection volume and HPLC grade methanol with 0.05% TFA (trifluoroacetic acid) as a mobile phase. The electronic absorption and fluorescence spectra were measured on a Shimadzu UV-Vis Multispec-1501 (Shimadzu, Kyoto, Japan) and a Hitachi F-7100 spectrophotometers (Hitachi, Tokyo, Japan), respectively.

The fluorescence quantum yields for the dyes were calculated by comparison to the fluorescence quantum yield of the reference, i.e., Coumarine I in ethanol (*Φ_ref_* = 0.64, Ex = 366 nm [28] for 2-PO, 4-PO, 2-PS and 4-PS), Coumarine 153 in ethanol (*Φ_ref_* = 0.38, Ex = 420 nm [29] for 2-PN, 4-PN, 2-QO, 4-QO, 2-QS and 4-QS) and Rhodamine 6G in ethanol (*Φ_ref_* = 0.94, Ex = 488 nm [29] for 2-QN and 4-QN).

The fluorescence decay curves were recorded on Edinburgh Instruments FLS920P Spectrometers (Edinburgh Instruments, Livingston, UK) using a time-correlated single-photon counting (TCSPC) method, employing picosecond diode lasers generating pulses of about 81.5 ps at 466.6 nm or 55 ps at 373 nm for excitation. The fluorescence decays were fitted to two- or tri-exponential functions using FAST program. The average lifetime, *τ_av_* is calculated as *τ_av_* = (Σ*_i_α_i_τ_i_*)/(*Σ_i_α_i_*), where *α_i_* and *τ_i_* are the amplitudes and lifetimes.

### 2.2. Computational Details

Density functional theory (DFT) approach implemented in Gaussian 16 program package [30] with PBE0/(6-311++G(d,p)/LANL2DZ) level of theory were applied to calculate the geometry of the tested dyes. Hessian analysis was used to check whether all structures correspond to minima on the potential energy surface. Time-dependent density functional theory (TDDFT/PBE0) allowed for the calculation of vertical absorption to characterize electronic properties [31]. Equation (1), which considers the numerical differentiation of the excitation energy (E) in the presence of an electric field F of 0.001 a.u. strength, allowed for the estimation of the dipole moments and the polarities of the charge transfer (CT) state.

(1) $${\Delta \mu }_{i}=\mu_{i}^{CT}-\mu_{i}^{GS}=\frac{E^{CT}\left({+F}_{i}\right)-E^{CT}\left({-F}_{i}\right)}{-2F_{i}}-\frac{E^{GS}\left({+F}_{i}\right)-E^{GS}\left({-F}_{i}\right)}{-2F_{i}},$$

where *i* stands for the Cartesian component of the dipole moment difference.

The density differences were obtained at the PBE0/6-311++G(d,p) level and are represented with a contour threshold of 0.02 a.u. The Le Bahers’ procedure was used to determine the charge transfer parameters, i.e., the charge transfer distance (*D*_CT_) and the amount of charge transferred (*q*_CT_) [32]. The Integral Equation Formalism for the Polarizable Continuum Model (IEF–PCM) was used to determine the solvent effect on the linear optical properties [33,34].

## 3. Results and Discussion

### 3.1. Spectral Properties

The target dyes were prepared by condensing quaternary salts with five-membered heterocyclic aldehydes in methanol using piperidine as a catalyst. The synthesis is based on the well-known method for obtaining hemicyanine dyes [23,24,25,26,27].

The structure and purity of the compounds were confirmed by IR, ^1^H, and ^13^C NMR spectra, as well as HPLC. The ^1^H NMR spectra show two doublets for the vinyl protons with a coupling constant of approximately 16 Hz, which corresponds to the values expected for the *trans* configuration. Furthermore, they exhibit one singlet at approximately 4.5 ppm, assigned to the methyl group in the pyridine(quinoline) subunit, and in the case of methylpyrrole derivatives, an additional singlet peak at approximately 3.8–3.9 ppm assigned to the methyl protons in this moiety.

From FT-IR analysis, the stretching vibrations for the C=C and C=N bonds in the pyridine and quinoline rings are in the range of 1600–1400 cm^−1^, as a strong or medium band and for C-H in the 3100–3000 cm^−1^ region. Bands in the region 1250–1000 cm^−1^ are due to C-H in-plane bending. However, the stretching vibration for C-N bonds are observed in similar range. Furthermore, the C-H out–of–plane bending vibration of the *trans*–vinyl group is observed around 950–980 cm^−1^. In general, the strong band attributed to the carbon-hydrogen bending vibrations of the =C-H group occurs in the region 1000–650 cm^−1^. The C-H stretching vibrations in the methyl group appear in the region of 2800–3000 cm^−1^. The methyl group gives also absorptions around 1380 cm^−1^ and 1480 cm^−1^ identified as the symmetrical and asymmetrical deformation vibrations, respectively.

The C-O-C symmetric and asymmetric stretching vibration at 1260–1000 cm^−1^ is characteristic for furan derivatives. Moreover, the C-H valence ring vibration bands in furan derivatives are at about 3125–3165 cm^−1^, the C=C valence ring vibration bands at about 1500 cm^−1^ and 1450 cm^−1^, and the C-H out-of-plane bending vibration bands at about 1010 cm^−1^ and 750 cm^−1^.

The IR spectra of compounds with thiophene subunit show the typical characteristic stretching vibration, C-C, stretching vibration of in-plane C-H, C-S, and C-H out-of-plane bending vibration absorption in the range of 1600–1400 cm^−1^, 1200–1000 cm^−1^ and 900–700 cm^−1^ [35].

The main features of the IR spectrum of pyrrole derivatives include bands for C=C ring vibrations in the range of 1650–1480 cm^−1^, weak and relatively sharp bands for C-H stretching vibrations in the ring in the range of 3100–3000 cm^−1^, skeletal vibrations of the ring in the range of 1000–1300 cm^−1^, deformational (bending) vibrations of C-H bonds in the in-plane and out-of-plane range of 1100 cm^−1^ and lower and in the range of 700–900 cm^−1^, respectively.

In HPLC only one peak was observed, confirming the absence of impurities in the synthesized compounds.

The detailed analysis is given in the ESI file. The data are consistent with the proposed structures.

### Spectroscopic Analysis

**Figure 2:**
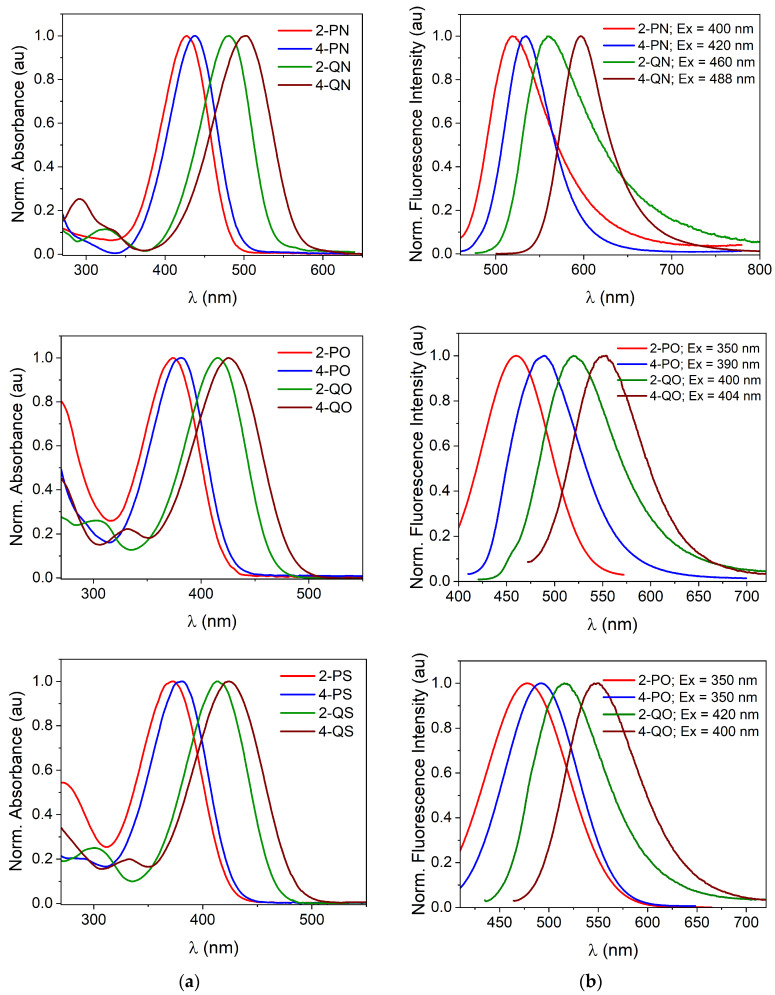
(**a**) Normalized absorption and (**b**) fluorescence spectra of the titled dyes recorded in DMSO at 20 °C.

The electronic absorption spectra of the titled compounds recorded in DMSO are presented in Figure 2a and their corresponding data are presented in Table 1.

These compounds exhibit a main absorption band in the range of 300–500 nm for thiophene and furan derivatives and 350–600 nm for methylpyrrole-based dyes, which are assigned to π–π* transitions of intramolecular charge transfer (ICT) nature. Their extinction coefficients (ε) range from ca. 2.0 × 10^4^ M^−1^ cm^−1^ to 5.0 × 10^4^ M^−1^ cm^−1^ and increase with elongation of the π-electron conjugation and the electronic capacity of the 5-membered heterocyclic ring.

Comparison of the UV-Vis spectra of ionic push-pull dyes clearly indicates that the elongation of the π-electron system of the acceptor group influences the position of the absorption band (Figure S1). Benzannulation of the pyridinium ring, leading to the formation of the quinoline structure, results in a shift in the absorption maximum toward longer wavelengths. For example, λ^ABS^ in DMSO moved from 427 nm, 438 nm, 374 nm, 381 nm, 372 nm and 381 nm to 482 nm, 502 nm, 414 nm, 425 nm, 414 nm and 424 nm for 2-PN, 4-PN, 2-PO, 4-PO, 2-PS and 4-PS, respectively. This effect can be attributed to two overlapping factors: (1) elongation of the conjugated system and (2) increased acceptor capacity of the quinoline system. Quinoline, as a structure with greater charge dispersion in the π system, allows for more efficient electron delocalization along the molecule, stabilizing the excited state and reducing the energy difference between the HOMO and LUMO. As shown in Table 2, the Δ*E*_GAP_ for the quinoline derivatives is lower, e.g., it changed from 2.4367 eV to 2.3091 eV for 2-PN and 2-QN, respectively. Consequently, the energy of absorbed radiation decreases, which manifests itself by a shift in the absorption maximum toward longer wavelengths.

A closer inspection of the electronic properties reveals that although quinoline derivatives generally exhibit enhanced electron delocalization and lower HOMO–LUMO gaps compared to their pyridinium analogues, the 4-QO and 4-QS compounds represent notable exceptions, showing larger Δ*E*_GAP_ values than the corresponding 4-PO and 4-PS. This anomaly can be rationalized by considering the combined effects of donor-heteroatom character, geometry, and solvent stabilization. In particular, the oxygen and sulfur substituents introduce strong inductive contributions that, in the *para*-substituted quinolinium framework, reduce the efficiency of resonance interactions and electron delocalization, thereby maintaining the LUMO at a higher energy level. Moreover, steric and geometric constraints associated with the quinolinium system limit planarity compared to pyridinium analogues, which is reflected in the density difference plots (Figure S3) as a less extended charge redistribution upon excitation. NBO analysis further indicates weaker iodine participation and lower N–I bond polarization in 4-QO and 4-QS relative to their pyridinium counterparts, which correlates with reduced charge-transfer efficiency. Finally, solvent-specific stabilization plays a role, as shown by lower *q*_CT_ and *D*_CT_ values in DMSO for these quinoline derivatives (Table S3), indicating less effective stabilization of the excited state. Altogether, these results demonstrate that while benzannulation usually promotes ICT, in the specific case of oxygen- and sulfur-substituted quinolinium derivatives the inductive effects, reduced planarity, and weaker donor–acceptor coupling outweigh the conjugation gain, resulting in an increased band gap.

The presence of an additional benzene ring also contributes to the increased electronic acidity of the acceptor system, which enhances the directional charge flow from the donor (e.g., thiophene, furan, or *N*-methylpyrrole) toward the acceptor. This effect enhances the push-pull nature of the molecule and further stabilizes the excited state.

The extent of the bathochromic shift also depends on the nature of the donor group. The largest shifts are observed for dyes containing *N*-methylpyrrole. The observed band shifts can be related both to the values of the constants σ^+^ for the individual heterocyclic systems—thiophene, furan, and *N*-methylpyrrole—and to the electronic properties of the heteroatoms themselves.

Based on literature data, it can be assumed that the lowest values of constants σ^+^ (−0.76 for thiophen-2-yl, −0.85 for furan-2-yl, −1.61 for pyrrol-2-yl) [36,37,38], indicating the relative strength of the donor effect of the substituents, will be exhibited by compounds containing *N*-methylpyrrole, followed by furan and thiophene. Therefore, for *N*-methylpyrrole derivatives, the most efficient charge transfer from the donor group toward the acceptor group can be expected, which promotes extended electronic delocalization in the molecule and leads to a red shift in the absorption maximum (bathochromic shift). Similarly, furan and thiophene, as weaker electron donors, should cause a smaller band shift.

Furthermore, the influence of substituents can also be interpreted in the context of the electronegativity of heteroatoms, which decreases in the following order: O, N, and S.

Higher electronegativity leads to a stronger inductive effect, which works opposite to the resonance effect. In the case of furan, despite the strong electronegativity of oxygen, the mesomeric effect predominates, allowing for a moderate absorption shift. For thiophene, the lower electronegativity of sulfur results in a weaker inductive effect and weaker charge transfer, which is reflected in the smallest band shift among the systems analyzed. In *N*-methylpyrrole, on the other hand, the presence of nitrogen allows for effective coupling to the π system, while exerting a moderate inductive influence, favoring a strong absorption band shift.

Moreover, a similar trend in the position of the absorption spectra is due to the molecular topology. In all cases, it was observed that position 4 (*para*) of the acceptor subunit relative to the conjugated donor moiety had an absorption maximum shifted toward longer wavelengths compared to their analogues at position 2 (*ortho*). This effect is particularly evident for dyes containing a stronger electron donor, i.e., *N*-methylpyrrole (Δλ^ABS^ = 11 nm for 4-PN vs. 2-PN, and Δλ^ABS^ = 20 nm for 4-QN vs. 2-QN in DMSO). Generally speaking, 4-conjugated systems, i.e., those in which the donor and acceptor are connected through a system enabling efficient, linear charge transfer along the conjugated π system, absorb longer-wavelength radiation. In *para* isomers, charge delocalization between the donor and acceptor is more effective, which promotes excited state stabilization and reduces the energy difference between the HOMO and LUMO. In *ortho* systems, spatial proximity of groups and steric constraints can disrupt the planar conformation of the molecule, reducing the efficiency of π-conjugation and consequently increasing the energy of absorbed radiation.

The obtained results clearly indicate that the position of the absorption band is the result of the synergistic effect of several factors, i.e., benzannulation of the *N*-methylpyridinium ring, electron-donating capacity of 5-membered heterocycles and mutual arrangement of the acceptor moiety in relation to the donor subunit.

The emission spectra show similar trends to the absorption spectra (Figure 2b and Figure S2). Depending on the relative position of the acceptor subunit (4 vs. 2) relative to the conjugated donor molecule, these bands are red shifted by 15 nm, 14 nm, and 22 nm for the PN, PS, and PO derivatives, and by 37 nm, 28 nm, and 29 nm for the QN, QS, and QO derivatives, respectively. Furthermore, the bathochromic shift in the fluorescence band with benzannulation of the methylpyridinium group due to the elongation of the conjugated system is 41 nm, 40 nm, and 56 nm for the compounds 2-QN vs. 2-PN, 2-QS vs. 2-PS, and 2-QO vs. 2-QP, respectively, and is even greater for compounds substituted at the 4-position of the 6-membered ring.

However, these compounds show very weak fluorescence. The low fluorescence quantum yields, which in most cases do not exceed 1%, can be rationalized by structural features that favor efficient non-radiative decay pathways. The vinyl bridge connecting the donor and acceptor fragments provides significant rotational freedom, which facilitates internal conversion and photoinduced isomerization processes that effectively compete with radiative relaxation. This interpretation is consistent with the electron density difference maps (Figure S4), which reveal pronounced charge depletion on the donor fragment and accumulation around the iodine-substituted aromatic ring. Such a redistribution weakens π–π coupling across the vinyl bridge and increases its flexibility in the excited state, favoring rotational relaxation. In addition, the presence of the heavy iodine atom enhances intersystem crossing (ISC) through the heavy-atom effect. NBO analysis of the N–I bond (Table S4) confirms a substantial iodine contribution (28–32%) and strong bond polarization, which support efficient spin–orbit coupling and facilitate non-radiative population of triplet states. Furthermore, the oscillator strengths calculated for some low-lying excitations are relatively small, suggesting that radiative deactivation competes poorly with alternative decay channels. In summary, these results indicate that the dominant structural factor governing the low fluorescence quantum yields is the rotational freedom around the vinyl bridge, which promotes internal conversion, while the presence of iodine further amplifies non-radiative deactivation through enhanced ISC. The explanation is consistent with the mechanism of multiple fluorescence of *o*-, *m*- and *p*-(dimethylamino)stilbazolium dyes proposed by Strehmel et al. [39]. Also, Fromherz et al. reported a relatively low (0.05%) fluorescence quantum yield for a rotatable compound ((aminophenyl)pyridinium) in highly polar and fluid solvents [40]. Another example of low fluorescence efficiency due to rotations and conformational changes in the molecule, effective charge transfer (ICT), fast internal conversion (IC), intersystem transitions (ISC) and the influence of the environment (polarity, pH) which compete with fluorescence emission, leading to its quenching are D–π–A compounds studied by us [41,42,43,44] and other research groups [26,38,45,46,47,48].

To better understand the photophysics of the studied compounds, their fluorescence decay curves were recorded in DMSO using a time-correlated single-photon counting (TCSPC) method and deconvoluted using a two- or tri-exponential function (Table 3). For *N*-methylpyrrole and furan derivatives, the time profile was found to exhibit fast (τ_1_) and slower decay (τ_2_) components. The presence of a broad, long-wavelength fluorescence band and two decay components in the tested molecules suggests the existence of two emitting states in polar solvents: an non-relaxed intramolecular charge transfer state (ICT)_NR_ and a relaxed ICT state (ICT)_R_ [44]. From the triexponential decay functions typical of thiophene derivatives, the fast decay lifetime was fitted to values of approximately 0.4–0.7 ns (τ_1_), the middle one to 0.83–1.09 ns (τ_2_), and the slow one to above 4 ns (τ_3_). Therefore, according to the model available in the literature for D–π–A salts [49,50,51], the longest fluorescence lifetime can be attributed to emission from the relaxed ICT state, the shortest to emission from the *E* (*trans*) isomer, and finally the middle lifetime to emission from the *Z* (*cis*) form [41].

Based on the calculated average fluorescence lifetimes and fluorescence quantum yields, the rate constants for the radiative and non-radiative rate constants were determined. According to the data summarized in Table 3, the deactivation of the excited state occurs mainly by non-radiative processes (the non-radiative rate constants are several orders of magnitude larger than the radiative ones), including the participation of the singlet excited state in the photoisomerization reaction, the singlet-triplet intersystem crossing and the internal conversion processes [41].

The steady-state and time-resolved results indicate the existence of several emitting forms of the tested push-pull dyes. The multiple fluorescence could essentially result from: (i) a possible transition from the locally excited π→π* (LE) state to the charge transfer (CT) state; (ii) intramolecular relaxation of the locally excited π→π* state involving rotation about single bonds at the double bond in the central part of the molecule [39].

The absorption and fluorescence spectra of the tested dyes were also analyzed in solvents of different polarities. The UV-vis spectroscopy of the titled compounds in 1,4-dioxane showed yellow and red solutions with λ_max_ ranging from 366 nm to 497 nm (Table 1). Dissolution in a more polar solvent such as DMSO causes a slight positive solvatochromic effect (red shift not exceeding 10 nm), resulting in λ_max_ of 372 nm and 502 nm for 2-PS and 4-QN.

Fluorescence shows a similar trend in a more polar environment, but the bathochromic effect is more pronounced. The emission maximum of thiophene derivatives was most solvent-dependent, with a red shift of 34 nm and 36 nm for 4-PS and 4-QS, respectively, when the environment was changed from 1,4-dioxane to DMSO.

**Figure 3:**
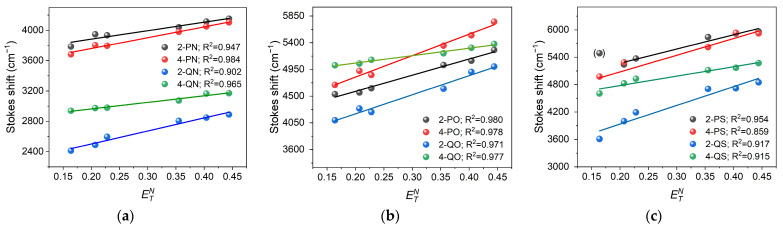
Dependence of Stokes shift on the $E_{T}^{N}$ polarity parameters of the solvents for: (**a**) 1-methyl-2-pyrrolyl derivatives; (**b**) 2-furyl derivatives; and (**c**) 2-thienyl derivatives; the point in parentheses was not included in the correlation.

The dyes studied exhibit a Stokes shift greater than 2400 cm^−1^, increasing with solvent polarity. The observed large shift between the absorption and fluorescence bands is due to the stronger ICT effect [16], indicating that the emitting state has a relaxed geometry, different from the geometry of the Franck-Condon state achieved by absorption [26]. This behavior also indicates a change in the dipole moment upon excitation, which is confirmed by the studies by Zhan and Wang [45] on the fluorescence properties of the 4-pyridinium analogue of our compound 4-PN in 20 solvents of different polarity and theoretical calculations described in the next subsection. The solvatochromic behavior of the dyes is caused by the ICT of the electron-donating 2-thienyl, 2-furyl, and 1-methyl-2-pyrrolyl groups to the electron-accepting pyridinyl or quinolinyl groups. To investigate the influence of different solvent properties on the Stokes shift, the $E_{T}^{N}$ Reichardt parameters and the Catalán four-parameter scale including: polarizability (SP), dipolarity (SdP), acidity (SA) and basicity (SB) parameters [52,53,54,55] were used. The $E_{T}^{N}$ scale, based on the extreme polarities of water and tetramethylsilane (TMS) used as reference, divides solvents into three groups: protic (from 0.5 to 1), dipolar, non-hydrogen donating (from 0.3 to 0.5) and apolar (from 0 to 0.3) [56]. A linear correlation with a satisfactory correlation coefficient between the Stokes shift and $E_{T}^{N}$ was found as shown in Figure 3. The observed trend indicates a clear influence of the environmental properties on the energy difference between the excited and ground states of the studied compounds. The positive slope of the regression line suggests that solvents with higher polarity stabilize the excited state to a greater extent than the ground state, leading to lower emission energy and thus an increased Stokes shift. This effect is typical of solvatochromic phenomena and confirms that the studied systems exhibit characteristic properties dependent on their surrounding environment. It should be emphasized that the highest values of the correlation coefficient (*R*^2^) for the fitted regression line are for most dyes, which indicates a good quality of the linear model fit to the experimental data and confirms the unambiguous relationship between the $E_{T}^{N}$ parameter and the Stokes shift.

The change in the Stokes shift value with environment was also correlated with the solvent parameters according to the Catalán model based on Equation (2).

(2) $${y=y}_{0}+a_{\mathrm{SP}}\mathrm{SP}+b_{\mathrm{SdP}}\mathrm{SdP}+c_{\mathrm{SA}}\mathrm{SA}+d_{\mathrm{SB}}\mathrm{SB},$$

where: *y*_0_ is the property of substance in interest in the absence of solvent, e.g., in the gas-phase and the *a*_SP_, *b*_SdP_, *c*_SA_, and *d*_SB_ are the corresponding coefficients of the solvent. The Catalán coefficients and the values of regression coefficients (R-square) determined by multilinear regression analysis are summarized in Table S1.

Fitting the Stokes shift values for most of the tested dyes to Equation (1) yield satisfactory fits, indicating high sensitivity of the dyes to environmental changes and the significant contribution of solvation parameters in shaping their photophysical properties. Only for compounds 4-QN and 2-PS the results were subject to a large error, suggesting the need to consider additional effects, such as specific intermolecular interactions or differences in electronic structure to analyze spectroscopic data in different environments.

In general, the shift between absorption and fluorescence λ_max_ is controlled by the overall effect of the solvent. As the data obtained show, the SdP scale provides an adequate description of the Stokes shift with some contribution from SA. The *b*SdP and *c*SA coefficients have high values with relatively low errors. Therefore, the Stokes shift strongly depends on the dipolarity and acidity of the solvent. In contrast, the influence of the solvent’s basicity and polarizability is smaller with higher uncertainty, although significant for most of the dyes tested. Based on the results, it can be concluded that non-specific and specific interactions must be considered when analyzing the solvatochromic properties of the dyes.

### 3.2. Theoretical Results

Studies on the electronic properties of the investigated compounds reveal significant structural and solvent-dependent relationships, which are reflected in the energies of frontier orbitals and parameters characterizing charge transfer. Comparing the electronic properties of both derivative series (PN/PO/PS and QN/QO/QS), it can be concluded that molecules with a thiophene ring exhibit the smallest HOMO–LUMO energy gap (Δ*E*_GAP_, Table S2), which translates into their highest susceptibility to excitation and strongest optical activity. On the other hand, compounds with a furan ring display the widest range of Δ*E*_GAP_ values depending on the environment, reaching a maximum for 2-QO in 1,4-Dx (2.92 eV). Structures with a pyrrole fragment show intermediate energy values, resulting in the highest stability of the charge transfer parameter *q*_CT_ (Table S3), regardless of the solvent. The spatial distribution of HOMO and LUMO (Figure S3) indicates strong orbital separation in *ortho* isomers, which is also reflected in the *q*_CT_ and *D*_CT_ values (Table S3). For 2-QS and 2-PO, the HOMO is mainly localized on the donor fragment, containing sulfur or oxygen, while the LUMO covers the iodine-bearing group and the adjacent aromatic ring. In *para* isomers, this separation weakens, as confirmed by lower *q*_CT_ values. An example is compound QS in DMSO, where the amount of transferred charge for the *ortho* isomer is 0.543 e, while for the *para* isomer, it drops to 0.308 e. Electron density difference plots (Δ*ρ*(r), Figure S4) reveal clear depletion zones located on the donor side of the molecule (–N(CH_3_)–, –O–, –S–), while the increase zones are concentrated near the iodine-containing fragment. Systems with strongly separated depletion and accumulation zones exhibit the highest *q*_CT_ and *D*_CT_ values. Changing the substituent position from *ortho* to *para* results in a shorter charge transfer distance and a reduced *q*_CT_ value. This effect is particularly pronounced in structures containing sulfur and oxygen. For PS derivatives in DMSO, *q*_CT_ drops from 0.559 e (*ortho*) to 0.542 e (*para*), while *D*_CT_ increases from 1.667 Å to 3.025 Å, indicating a shift in the charge transfer mechanism. A similar trend is observed in the PO series; for 2-PO in THF, the values are *q*_CT_ = 0.481 e and *D*_CT_ = 2.355 Å, while for 4-PO, these values drop to 0.333 e and 1.557 Å, respectively, although the *D*_CT_ increases again in DMSO. Although the *ortho* derivatives exhibit stronger local bond polarization, as confirmed by NBO analysis (Table S4), which can give the impression of more intense intramolecular charge-transfer within the donor fragment, the quantitative parameters, *q*_CT_ and *D*_CT_, clearly demonstrate that *para* connectivity provides more efficient long-range charge transfer. In *para* systems, extended conjugation along the *π*-bridge favors global delocalization of electron density, which correlates with larger excited-state dipole moments and bathochromically shifted absorption maxima. Thus, the *ortho* arrangement enhances local polarization effects, whereas the *para* geometry enables more effective overall charge delocalization and stabilization of the excited state.

Moreover, the theoretical calculations indicate that thiophene-containing derivatives exhibit the lowest HOMO–LUMO energy gaps, experimental absorption spectra show that *N*-methylpyrrole derivatives (PN/QN) possess the most bathochromically shifted bands. This discrepancy likely arises from the fact that Δ*E*_GAP_ alone does not fully determine the absorption maximum position. The overall electronic conjugation, donor strength, and molecular topology, particularly the enhanced electronic coupling in NMe-containing systems, play a dominant role in stabilizing the excited state and shifting the absorption to longer wavelengths.

Although theoretical calculations indicate that thiophene-containing derivatives exhibit the lowest HOMO–LUMO energy gaps, experimental absorption spectra show that *N*-methylpyrrole derivatives (PN/QN) possess the most bathochromically shifted bands. This discrepancy likely arises from the fact that Δ*E*_GAP_ alone does not fully determine the absorption maximum position. The overall electronic conjugation, donor strength, and molecular topology, particularly the enhanced electronic coupling in NMe-containing systems, play a dominant role in stabilizing the excited state and shifting the absorption to longer wavelengths. It should also be emphasized that this difference reflects inherent limitations of the TDDFT methodology. While the PBE0 functional with the applied basis set reproduces general excitation energy trends, it does not always capture the balance between orbital contributions, donor–acceptor interactions, and solvent stabilization effects in highly polarizable charge-transfer systems. Moreover, absorption maxima depend not only on the HOMO–LUMO gap but also on the relative oscillator strengths of the low-energy transitions. In *N*-methylpyrrole derivatives, the most intense transitions involve orbital configurations beyond the simple HOMO→LUMO excitation, which results in the pronounced bathochromic response observed experimentally. Conversely, in thiophene derivatives the HOMO–LUMO transition has low oscillator strength, diminishing its manifestation in the experimental spectra despite the narrow calculated gap. This highlights the importance of considering both transition energies and oscillator strengths when comparing TDDFT predictions with experimental absorption spectra.

Natural Bond Orbital (NBO, Table S4) analysis of the N–I bond confirms the active role of iodine in modulating the molecule’s properties. The iodine contribution to the bonding orbital ranges from 28% to 32%, with a higher contribution and stronger bond polarization observed in *ortho* isomers. For example, for 2-QS in DMSO, iodine contributes 28.10% and nitrogen 71.84%, with hybridization sp^7.04^/sp^14.70^. For 4-QS in DMSO, iodine’s contribution rises to 30.15%, despite a lower charge transfer effect, confirming that the molecular geometry influences the direction and efficiency of charge migration. Moreover, in 1,4-Dx for 2-PN, the polarity of the N–I bond is 69.78%, while for 4-PN it is slightly lower (68.64%). In sulfur-containing systems (2-PS) in the same solvent, higher polarity (70.04%) is observed compared to oxygen analogues (2-PO: 69.95%), which correlates with higher *q*_CT_ values. It is also noteworthy that the *s*-orbital contribution to nitrogen hybridization systematically increases across the solvent series, e.g., for 2-PN: 11.65% in 1,4-Dx, 11.58% in THF, and 11.57% in DMSO, indicating possible changes in bonding character under different environments. The presence of iodine, as a large and highly polarizable atom, facilitates charge localization in its vicinity and increases the system’s electrophilicity, which is evident in the values of the global electrophilicity index *ω* (Table S2).

The solvent environment also significantly affects the molecular properties. A clear trend is observed toward a reduction in the HOMO–LUMO gap with increasing solvent polarity. In many cases, an increase in *q*_CT_ is also observed, indicating a favorable impact of polar environments on the stabilization of excited states. The thermodynamic analysis of solvation processes in the studied systems reveals complex correlations between the solvation free energy (Δ*G*_solv_) and electronic parameters. For 2-PN and 4-PN isomers, the most negative Δ*G*_solv_ values in THF (–20.54 kcal/mol and –21.14 kcal/mol, respectively) correspond to a characteristic set of electronic parameters: Δ*E*_GAP_ of 2.5959 eV and 2.3820 eV, and *q*_CT_ values of 0.539 e and 0.351 e, respectively. This correlation indicates a significant role of specific donor–acceptor interactions between THF and the studied molecules, where the THF oxygen atom forms strong complexes with Lewis centers (N, I) in PN molecules [57], resulting in effective stabilization of the ground state. On the other hand, in DMSO, despite intermediate Δ*G*_solv_ values (–12.66 kcal/mol for 2-PN and –12.31 kcal/mol for 4-PN), the lowest Δ*E*_GAP_ values (2.4367 eV and 2.3665 eV) and high *q*_CT_ values (0.537 e and 0.351 e) are observed. This can be attributed to the strong polarization of bonds by the static dipole moment of DMSO, which efficiently stabilizes transition states and reduces Δ*E*_GAP_. Simultaneously, this effect may be limited by steric constraints due to DMSO’s molecular size, which reduces solvation efficiency. Additionally, strong DMSO–DMSO intermolecular associations may compete with solute–solvent interactions, explaining the less negative Δ*G*_solv_ values. Of particular importance are observations for sulfur-containing systems (PS). For 2-PS in DMSO (Δ*G*_solv_ = –11.44 kcal/mol), an exceptionally high *q*_CT_ value (0.559 e) is observed, whereas the Δ*G*_solv_ difference between DMSO and THF is less pronounced than for oxygen analogues (PO). This effect can be attributed to enhanced charge delocalization via the sulfur atom, as confirmed by NBO calculations showing higher bond polarity in sulfur-containing systems (70.04% for 2-PS and 69.95% for 2-PO in 1,4-Dx). This extended charge delocalization reduces sensitivity to local solvation effects, resulting in smaller differences in Δ*G*_solv_ between solvents while maintaining high charge transfer efficiency.

Comparison of theoretical (*λ*_ABS_, Table S6) and experimental absorption maxima for the studied compounds reveals systematic shifts and varying accuracy across structural series. In general, the TDDFT method slightly underestimates the excitation energy and the mean absolute error between the calculated and measured values (${∆\lambda }_{ABS}^{TDDFT-Exp}$) was estimated at approximately 5.30 nm, with the highest deviations observed for 2-PS and 4-QS compounds in DMSO, reaching up to 14.47 nm and 14.71 nm, respectively. The smallest discrepancies were found for 4-PN in 1,4-DX, where ${∆\lambda }_{ABS}^{TDDFT-Exp}$ = 0.7 nm. Additionally, slightly higher ${∆\lambda }_{ABS}^{TDDFT-Exp}$ values were noted for derivatives with *para* substituents (5.72 nm). These results indicate that the agreement between theoretical calculations and experimental data depends on both solvent and molecular structure, with *ortho*/*para* isomerism also playing a significant role. In terms of absorption band position, PN/QN derivatives containing the –N(CH_3_)– group exhibit the most bathochromically shifted absorption maxima, with wavelengths exceeding 490 nm (491.85 nm and 505.42 nm in DMSO for 2-QN and 4-QN, respectively). In contrast, derivatives with an –O– group (PO/QO) show the most hypsochromic shifts, with maxima in the 370–430 nm range. The PS/QS compounds, incorporating the –S– group, occupy an intermediate position. These trends clearly indicate that the electron-donating strength and polarizability of the substituents are crucial in modulating electronic transitions. For PN/QN derivatives, the stronger donor –N(CH_3_)– more effectively stabilizes the excited state, resulting in bathochromic shifts. Additionally, changing the substituent position from *ortho* to *para* leads to longer wavelength shifts due to increased conjugation and extended electron delocalization in *para* isomers, which favor further excited state stabilization.

The nature and magnitude of absorption band shifts are closely related to dipole moments and excited-state polarity (${\Delta \mu }_{CT-GS}$, Table S7). Compounds showing greater increases in dipole moment upon excitation also exhibit more pronounced bathochromic shifts. For QS in 1,4-Dx, ${\Delta \mu }_{CT-GS}$ increases from 8.00 D (*ortho*) to 13.66 D (*para*), corresponding to a shift from 416.67 nm to 421.98 nm. A similar relationship is observed for QN in DMSO, where ${\Delta \mu }_{CT-GS}$ increases from 12.31 D (*ortho*) to 17.26 D (*para*), with absorption maxima shifting from 491.85 nm to 505.42 nm. Likewise, for PN in 1,4-Dx, ${\Delta \mu }_{CT-GS}$ rises from 8.51 D to 10.87 D, and the absorption maximum shifts from 423.99 nm to 438.70 nm. For PS in THF, the transition from the *ortho*-isomer ${\Delta \mu }_{CT-GS}$ ( = 7.81 D) to *para*-isomer (${\Delta \mu }_{CT-GS}$ = 10.33 D) results in a shift from 425.91 nm to 438.89 nm. These correlations confirm that increasing the polarity of the excited state stabilizes it and leads to red shifts in the absorption bands. This relationship confirms that stronger ICT character results in greater stabilization of the excited state and thus longer wavelength absorption. Moreover, higher dipole moments in both ground and excited states are typically observed for *para* isomers, particularly in polar solvents like DMSO. The polar nature of the excited state enhances interactions with the polar environment, leading to additional stabilization through solute–solvent interactions. This stabilization lowers the excited state energy more significantly than that of the ground state, effectively reducing excitation energy and causing bathochromic shifts in absorption maxima. This phenomenon is particularly evident in QN and QS derivatives, where the combined effects of *para*-substitution, strong donor groups, and polar environments lead to pronounced absorption shifts.

Theoretical absorption spectra obtained via TDDFT calculations (Figure S4) show strong qualitative agreement with experimentally recorded spectra. In both cases, dominant absorption bands corresponding to *π*–π* transitions are observed, with their position depending on molecular structure. An important aspect of the agreement between the two methods is the presence of additional hypsochromically shifted bands relative to the main band. These can be attributed to transitions of lower oscillator strength or mixed *π*–π*/*n*–π* character, as well as possible vibronic states. Although TDDFT is based on a simplified continuum model and does not explicitly account for vibrational coupling, it reflects these transitions as additional excited states with relatively lower oscillator energies, thereby confirming the existence of secondary bands observed experimentally. The consistency of trends between theory and experiment is also reflected in the relative differences in band intensities. In both approaches, the dominant transition corresponding to ICT in QN and QS spectra is clearly more intense than other signals. Additional peaks appear in the 370–420 nm range, especially in structures containing sulfur or oxygen atoms, suggesting the presence of localized transitions on the donor heteroaromatic ring. Theoretical spectra show a similar distribution of excited states, confirming the experimental observations. Moreover, in QO and PO derivatives, weak bands appear in the measured spectra that are also found in theoretical spectra as low-intensity transitions. Their theoretical identification allows them to be assigned to HOMO–1→LUMO or HOMO→LUMO+1 type transitions, often localized and weakly coupled to the global ICT transition.

The spectroscopic properties of the investigated derivatives strongly depend on intramolecular electronic structure, as evidenced by consistent results from HOMO–LUMO gap analysis, Δ*ρ*(r) mapping between ground and excited states, and the N–I bond characterization from NBO analysis. The Δ*E*_GAP_ directly correlates with the position of the absorption maximum and with the intensity and nature of ICT transitions. Compounds with the lowest Δ*E*_GAP_ values, such as QN and QS derivatives in DMSO, also exhibit the largest bathochromic shifts in absorption spectra, indicating strong stabilization of the excited state relative to the ground state. Conversely, Δ*ρ*(r) maps upon excitation reveal clear depletion zones on the donor fragment containing the –N(CH_3_)–, –O–, or –S– groups, and accumulation zones near the iodine and adjacent aromatic ring. This spatial separation confirms the charge-transfer nature of the transition. This promotes increased dipole moments and greater excited-state polarity. Additionally, NBO analysis of the N–I bond confirms iodine’s active role in charge transfer, directly influencing the transition character and absorption band position. In compounds with a strong ICT effect, a greater contribution of iodine to the bonding orbital and higher N–I bond polarity are observed. At the same time, N–I bonds are more polarized in *ortho* systems, leading to stronger local donor–acceptor effects, though not necessarily resulting in lower Δ*E*_GAP_. Thus, spatial charge separation, better achieved in *para* systems, is more significant in determining transition energy. For 4-QN in DMSO, Δ*E*_GAP_ = 2.2564 eV, ${\Delta \mu }_{CT-GS}$ = 17.26 D, and the Δ*ρ*(r) map shows a distinctly localized accumulation zone near iodine, consistent with the bathochromic shift in the absorption maximum. Conversely, PO/QO derivatives exhibit weaker delocalization, lower ${\Delta \mu }_{CT-GS}$, and higher Δ*E*_GAP_, resulting in absorption bands shifted toward shorter wavelengths. Theoretical data show higher *q*_CT_ and *D*_CT_ values for *ortho* isomers compared to their *para*-analogues, which appears inconsistent with the more pronounced charge transfer signatures observed experimentally for *para* compounds. This contradiction can be attributed to differences in the nature of the charge transfer: while *ortho* isomers exhibit stronger localized donor–acceptor interactions, the *para* topology allows for more extended π-conjugation and global charge delocalization across the molecule. Consequently, *para* derivatives benefit from more efficient stabilization of the excited state, which results in experimentally observed bathochromic shifts despite lower local Δ*ρ*(r) values.

## 4. Conclusions

This article discusses the synthesis of twelve push-pull dyes and their spectroscopic properties, obtained based on measurements using steady-state and time-resolved spectroscopic methods. These compounds exhibit a major absorption band in the visible range, which is attributed to π–π* transitions of intramolecular charge transfer (ICT) nature. Its intensity ranges from approximately 2.0 × 10^4^ M^−1^ cm^−1^ to 5.0 × 10^4^ M^−1^ cm^−1^. They are characterized by a large Stokes shift, greater than 2400 cm^−1^, and low fluorescence intensity. The fluorescence quantum yields in most cases do not exceed 1%. Steady-state and time-resolved results indicate the existence of several emitting species.

The position of the absorption and fluorescence spectra depends on the dye structure. Benzannulation of the pyridinium ring to quinoline is found to be an effective way to modulate the optical properties of the dyes, allowing for control of the absorption and fluorescence band position by enhancing the charge shift effect and extending the conjugation length within the molecule.

With respect to electron donors in the studied compounds, the observed changes in the positions of absorption and fluorescence bands result from a combination of resonance and inductive effects, controlled by the electronic properties of the five-membered heterocyclic subunits. Stronger electron donors (lower σ+, lower electronegativity) lead to a stronger push effect and a shift in the maxima toward longer wavelengths, which is consistent with the repulsive-attractive nature of the dyes.

The topology of the donor-acceptor interface (2 vs. 4) is also crucial for charge transfer efficiency in the push-pull dyes. The 4-position promotes better electronic coupling and shifts the absorption and fluorescence band toward longer wavelengths, making it more favorable for designing materials with specific optoelectronic properties.

The positions of the absorption and fluorescence bands also depend on the solvent polarity. Analysis of the effect of various solvent properties on the Stokes shift using one- and multiparameter scales shows that solvents with higher polarity stabilize the excited state to a greater extent than the ground state, leading to a lower emission energy and thus a larger Stokes shift. Furthermore, the dipolarity component provides an adequate description of the Stokes shift, with some contribution from solvent acidity.

The experimental results are supplemented with theoretical calculations. The quantitative parameters, *q*_CT_ and *D*_CT_, clearly demonstrate that *para* connectivity provides more efficient long-range charge transfer. Natural Bond Orbital analysis of the N–I bond confirms the active role of iodine in modulating the molecule’s properties. The iodine contribution to the bonding orbital ranges from 28% to 32%. Furthermore, a clear trend is observed toward a reduction in the HOMO–LUMO gap with increasing solvent polarity. In many cases, an increase in *q*_CT_ is observed, indicating a favorable impact of polar environments on the stabilization of excited states. The nature and magnitude of absorption band shifts are closely related to dipole moments and excited-state polarity. Compounds showing greater increases in dipole moment upon excitation also exhibit more pronounced bathochromic shifts.

The obtained results indicate that by appropriately modifying the chemical structure of an organic compound, we can modulate its physicochemical and optical properties for specific applications. We have demonstrated that heteroatoms in heterocyclic electron donors, elongation of the conjugated system through benzannulation, and the position of the donor moiety relative to the acceptor moiety have a synergistic effect on the spectroscopic properties of the push-pull dyes.

## Figures and Tables

**Table 1:** Photophysical properties of the tested dyes.

No	Structural Formula	Solvent	$\lambda_{max}^{ABS}$ (nm)	ε (M^−1^ cm^−1^)	$\lambda_{max}^{FL}$ (nm)	*Φ*^FL^ (%)	Δν^SS^ (cm^−1^)
2-PN	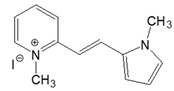	DMSO	427	31,000	519	0.19	4151
		DMF	427	34,300	518	0.16	4114
		MeAc	427	35,400	516	0.19	4039
		EtOA	422	–	506	0.12	3934
		THF	428	–	517	0.41	4022
		1,4-Dx	419	–	498	0.97	3786
4-PN	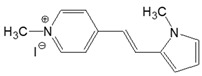	DMSO	438	37,700	534	1.22	4104
		DMF	437	36,300	531	0.87	4051
		MeAc	437	42,500	529	0.74	3980
		EtOAc	435	–	521	0.24	3795
		THF	439	–	527	0.41	3804
		1,4-Dx	435	–	518	0.57	3683
2-QN	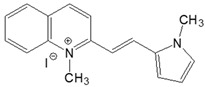	DMSO	482	50,800	560	0.40	2890
		DMF	480	49,800	556	0.25	2848
		MeAc	478	46,400	552	0.44	2805
		EtOAc	479	–	547	0.23	2595
		THF	483	–	549	1.60	2489
		1,4-Dx	484	–	548	1.87	2413
4-QN	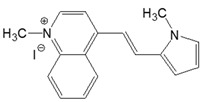	DMSO	502	37,600	597	1.51	3170
		DMF	500	32,000	594	1.02	3165
		MeAc	498	37,700	588	0.46	3074
		EtOAc	496	–	582	0.22	2979
		THF	499	–	586	0.71	2975
		1,4-Dx	497	–	582	1.51	2939
2-PO	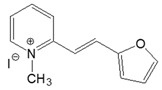	DMSO	374	24,600	466	0.03	5279
		DMF	372	26,100	459	0.02	5095
		MeAc	371	22,400	456	0.03	5024
		EtOAc	371	–	448	0.01	4633
		THF	372	–	448	0.11	4560
		1,4-Dx	371	–	446	0.12	4533
4-PO	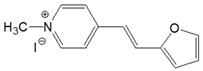	DMSO	381	31,200	488	0.23	5755
		DMF	380	29,200	481	0.17	5526
		MeAc	380	33,600	477	0.17	5351
		EtOAc	376	–	460	0.04	4857
		THF	379	–	466	0.08	4926
		1,4-Dx	375	–	455	0.41	4689
2-QO	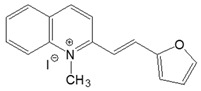	DMSO	414	28,900	522	0.32	4998
		DMF	413	33,300	518	0.22	4908
		MeAc	412	29,300	509	0.16	4625
		EtOAc	412	–	499	0.05	4237
		THF	411	–	499	0.22	4291
		1,4-Dx	413	–	497	0.44	4092
4-QO	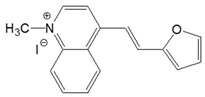	DMSO	425	26,100	551	0.40	5381
		DMF	422	38,600	544	0.37	5314
		MeAc	420	27,700	538	0.29	5222
		EtOAc	417	–	530	0.06	5113
		THF	420	–	533	0.27	5048
		1,4-Dx	418	–	529	0.70	5020
2-PS	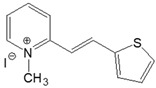	DMSO	372	20,700	478	0.02	5981
		DMF	372	20,500	477	0.01	5917
		MeAc	370	23,800	472	0.01	5841
		EtOAc	365	–	454	0.01	5371
		THF	370	–	459	0.03	5241
		1,4-Dx	366	–	458	0.13	5488
4-PS	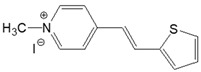	DMSO	381	21,700	492	0.08	5922
		DMF	379	25,800	489	0.05	5935
		MeAc	378	21,600	480	0.04	5622
		EtOAc	373	–	457	0.02	4928
		THF	379	–	474	0.05	5288
		1,4-Dx	373	–	458	0.32	4976
2-QS	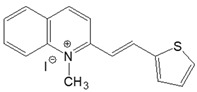	DMSO	414	38,100	515	0.10	4737
		DMF	413	40,500	512	0.08	4682
		MeAc	410	40,000	508	0.07	4705
		EtOAc	410	–	498	0.03	4310
		THF	410	–	506	0.05	4627
		1,4-Dx	412	–	480	0.04	3439
4-QS	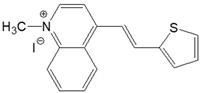	DMSO	424	29,100	546	0.32	5270
		DMF	424	30,400	543	0.20	5169
		MeAc	420	28,100	535	0.13	5118
		EtOAc	414	–	528	0.05	4927
		THF	422	–	530	0.12	4829
		1,4-Dx	413	–	510	0.10	4605

**Table 2:** The frontier orbital energies and energy gap (Δ*E*_GAP_) calculated for DMSO by DFT/PBE0 with the 6-311++G(d,p)/LANL2DZ basis set.

Dye	E_HOMO_	E_LUMO_	Δ*E*_GAP_	Dye	E_HOMO_	E_LUMO_	Δ*E*_GAP_
2-PN	−4.5314	−2.0946	2.4367	4-PN	−4.5700	−2.2035	2.3665
2-QN	−4.5414	−2.2324	2.3091	4-QN	−4.5442	−2.3232	2.2209
2-PO	−4.5586	−2.7129	1.8456	4-PO	−4.4522	−2.7233	1.7289
2-QO	−4.4220	−2.7810	1.6410	4-QO	−4.6979	−2.4707	2.2272
2-PS	−4.5746	−2.7304	1.8443	4-PS	−4.4707	−2.7361	1.7346
2-QS	−4.4421	−2.8008	1.6413	4-QS	−4.7055	−2.5099	2.1956

**Table 3:** Fluorescence lifetime (τ; ns), their amplitudes (α; %) and correlation coefficients (χ^2^), radiative (^av^*k_r_*; 10^6^ s^−1^) and non-radiative (^av^*k_nr_*; 10^9^ s^−1^) rate constant for the tested dyes in DMSO.

No	*τ_1_*	*α_1_*	*τ_2_*	*α_2_*	*τ_3_*	*α_3_*	*τ_av_*	χ^2^	^av^ *k_r_*	^av^ *k_nr_*	*k_nr_/k_r_*
2-PN	0.07	89.0	1.47	11.0			0.224	1.43	8.48	4.46	525.3
4-PN	0.08	97.5	1.69	2.5			0.120	1.47	101.0	8.21	81.0
2-QN	0.03	92.0	6.6	8.0			0.556	1.52	7.20	1.79	249.0
4-QN	0.08	99.5	3.32	0.5			0.096	1.57	157.0	10.2	65.2
2-PO	0.04	62.2	0.98	21.2	5.33	16.6	1.117	1.11	0.27	0.90	3332.3
4-PO	0.06	97.2	1.77	2.8			0.108	1.55	21.3	9.25	433.8
2-QO	0.06	91.5	1.9	8.5			0.216	1.22	14.8	4.61	311.5
4-QO	0.12	62.2	2.02	37.8			0.838	1.30	4.77	1.19	249.0
2-PS	0.05	66.2	0.83	17.1	4.23	16.7	0.881	1.02	0.23	1.13	4999.0
4-PS	0.04	87.0	0.89	6.3	4.34	6.7	0.382	1.02	2.10	2.62	1249.0
2-QS	0.07	77.9	1.09	11.0	4.04	11.1	0.623	1.08	1.61	1.60	999.0
4-QS	0.06	85.0	0.84	7.7	4.41	7.3	0.438	1.15	7.31	2.28	311.5

## Data Availability

The original contributions presented in this study are included in the article/Supplementary Material. Further inquiries can be directed to the corresponding author.

## Supplementary Materials

The following supporting information can be downloaded at: https://www.mdpi.com/article/10.3390/ma18204676/s1, description of synthesis; IR and NMR spectra; HPLC; Figure S1: Electronic absorption spectra of the tested dyes in solvents of different polarity; Figure S2: Fluorescence spectra of the tested dyes in solvents of different polarity; Figure S3: The HOMO/LUMO plots; Figure S4: The density difference plot; Figure S5: Graphical representation of theoretically determined absorption spectra; Table S1: Data from the Catalán solvent scale analysis; Tables S2: The frontier orbital energies; Table S3: CT parameters for the bright low-lying excited state; Table S4: Occupancy, energy and polarity of natural bond orbitals (NBOs) and hybrids calculated for investigated compounds; Table S5: Solvation free energy values; Table S6: Theoretical maxima of absorption bands; Table S7: Calculated values of dipole moments for the ground and CT excited state. References [24,25,38,46,58,59,60,61,62] are cited in the Supplementary Materials.
