# Supplementary material for: Both Benzannulation and Heteroatom-Controlled Photophysical Properties in Donor–π–Acceptor Ionic Dyes: A Combined Experimental and Theoretical Study
Source: Materials (Basel). 2025 Oct 12;18(20):4676. doi: 10.3390/ma18204676 (PMC12565802; doi:10.3390/ma18204676)
Supplement: Supplementary file 1 [file materials-18-04676-s001.zip › materials-3903653-supplementary.pdf]

ELECTRONIC SUPPORTING INFORMATION

# Both Benzannulation and Heteroatom-Controlled Photophysical Properties in Donor-π-Acceptor Ionic Dyes: A Combined Experimental and Theoretical Study

Przemysław Krawczyk[1] and Beata Jędrzejewska[2,*]

[1] Nicolaus Copernicus University, Collegium Medicum, Faculty of Pharmacy, Kurpińskiego 5, 85-096 Bydgoszcz, Poland; przemekk@cm.umk.pl
[2] Bydgoszcz University of Science and Technology, Faculty of Chemical Technology and Engineering, Seminaryjna 3, 85-326 Bydgoszcz, Poland; beata@pbs.edu.pl
* Correspondence: beata@pbs.edu.pl; Tel.: +48 52 374 90 46, B.J.

Table of contents

Content	page
Synthesis	S2-S6
^{1}H and ^{13}C NMR spectra	S7-S18
IR spectra	S19-S24
HPLC chromatograms	S25-S31
Electronic absorption spectra in solvents of different polarity – **Figs S1**	S32
Fluorescence spectra in solvents of different polarity – **Fig. S2**	S33
Data from the Catalán four-parameter solvent scale analysis – **Table S1**	S34
The HOMO/LUMO plots – **Fig. S3**	S35-S36
The density difference plot – **Fig. S4**	S37
Theoretical absorption spectra – **Fig. S5**	S38
The frontier orbital energies – **Table S2**	S39
CT parameters for the bright low-lying excited state – **Table S3**	S40
Occupancy, energy and polarity of natural bond orbitals (NBOs) and hybrids calculated for investigated compounds – **Table S4**	S40-S42
Solvation free energy values – **Table S5**	S43
Theoretical maxima of absorption bands – **Table S6**	S43
Calculated values of dipole moments for the ground and CT excited state – **Table S7**	S44

Synthesis

In a 50 ml flask equipped with a magnetic stirrer and a reflux condenser, 20 mL of anhydrous methanol, 4 mmol of the appropriate heterocyclic aldehyde, 4 mmol of the quaternary salt of pyridine or quinoline and a catalytic amount of piperidine were placed and stirred for 24 h at 25-30 °C. In the case of *N*-Methyl-2-pyrrolecarboxaldehyde and thiophene-2-carboxaldehyde, after the reagents were dissolved, the mixture was heated at reflux for 4 h. It was then cooled to room temperature and frozen in a freezer. The precipitate was filtered off and washed several times with diethyl ether, dried, and then recrystallized from ethanol.

***2-(2-furan-2-yl-vinyl)-1-methyl-pyridinium iodide* (2-PO)**

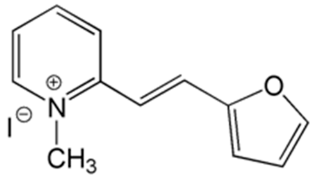

The compound was obtained as yellow crystals; $C_{12}H_{12}INO$; yield: 47.5 %; 313.13 g/mol; mp 203-205 °C lit. 204-206 °C [1] and 218-219 °C [2]

^{1}H NMR (400 MHz, DMSO-d_6) δ (ppm): 8.92-8.90 (d, *J*=8.0 Hz, 1H), 8.49 (m, 2H), 8.00-7.99 (d, *J*=4.0 Hz, 1H), 7.89-7.85 (d, *J*=16.0 Hz, 1H), 7.87 (m, 1H), 7.25-7.21 (d, *J*=16.0 Hz, 1H), 7.08-7.07 (d, *J*=4.0 Hz, 1H), 6.75 (dd, *J*=4.0 Hz, 1H), 4.34 (s, 3H, CH_3);

$^{13}C\{^{1}H\}$ NMR (100 MHz, DMSO-d_6) δ (ppm): 152.4 (C), 151.5 (C), 147.1 (CH), 146.5 (CH), 144.6 (CH), 129.7 (CH), 125.3 (CH), 125.0 (CH), 117.1 (CH), 114.8 (CH), 113.8 (CH), 46.4 (CH_3);

IR (KBr) $\tilde{\nu}$ (cm^{-1}): 3142 (-CH in furan), 1541, 1033, 759 (vibrations of the furan ring), 3062, 1607, 1204, 1090, 1032, 897 (-CH in pyridine), 1483 (vibrations of the pyridine ring), 1658 (C=N), 2903 (NCH_3), 982 (=CH).

***4-(2-furan-2-yl-vinyl)-1-methyl-pyridinium iodide* (4-PO)**

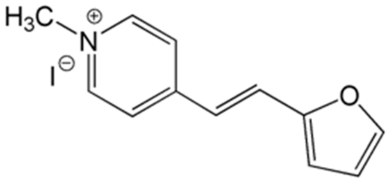

The compound was obtained as yellow crystals; $C_{12}H_{12}INO$; yield: 25 %; 313.13 g/mol; mp 186-188 °C lit 202-203 °C [3]

^{1}H NMR (400 MHz, DMSO-d_6) δ (ppm): 8.83-8.81 (d, *J*=8.0 Hz, 2H), 8.21-8.19 (d, *J*=8.0 Hz, 2H), 7.95-7.94 (d, *J*=4.0 Hz, 1H), 7.90-7.86 (d, *J*=16.0 Hz, 1H), 7.20-7.16 (d, *J*=16.0 Hz, 1H), 6.95-6.94 (d, *J*=4.0 Hz, 1H), 6.71 (m, 1H), 4.23 (s, 3H, CH_3);

$^{13}C\{^{1}H\}$ NMR (100 MHz, DMSO-d_6) δ (ppm): 152.6 (C), 151.8 (C), 146.8 (CH), 145.4 (CH), 127.9 (CH), 123.7 (CH), 120.9 (CH), 116.3 (CH), 113.7 (CH), 47.3 (CH_3);

IR (KBr) $\tilde{\nu}$ (cm^{-1}): 3127 (-CH in furan), 1543, 1035, 754 (vibrations of the furan ring), 3066, 1596, 1204, 1090, 1035, 849 (-CH in pyridine), 1497 (vibrations of the pyridine ring), 1666 (C=N), 2905 (NCH_3), 982 (=CH).

***1-methyl-2-(2-thiophen-2-yl-vinyl)-pyridinium iodide* (2-PS)**

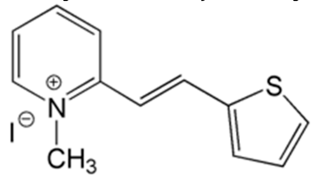

The compound was obtained as yellow crystalline powder; $C_{12}H_{12}INS$; yield: 79 %; 329.2 g/mol; mp 228-230 °C lit. 231-232 °C [4] and 225-229 °C [2]

^{1}H NMR (400 MHz, DMSO-d_6) δ (ppm): 8.89-8.88 (d, *J*=4.0 Hz, 1H), 8.48 (m, 2H), 8.20-8.16 (d, *J*=16.0 Hz, 1H), 7.89 (m, 1H), 7.87-7.86 (d, *J*=4.0 Hz, 1H), 7.68-7.67 (d, *J*=4.0 Hz, 1H), 7.28-7.24 (d, *J*=16.0 Hz, 1H), 7.26 (m, 1H), 4.35 (s, 3H, CH_3);

^{13}C{^{1}H} NMR (100 MHz, DMSO-d_6) δ (ppm): 152.5 (C), 146.4 (CH), 144.6 (C), 140.3 (CH), 136.2 (CH), 132.4 (CH), 131.5 (CH), 129.3 (CH), 125.3 (CH), 125.1 (CH), 116.2 (CH), 46.4 (CH_3);

IR (KBr) $\tilde{\nu}$ (cm^{-1}): 3088 (-CH in thiophene), 1540, 1036, 723 (vibrations of the thiophene ring), 3057, 1591, 1193, 1091, 1065, 840, 791 (-CH in pyridine), 1631, 1481 (vibrations of the pyridine ring), 1656 (C=N), 2905 (NCH_3), 964 (=CH).

***1-methyl-4-(2-thiophen-2-yl-vinyl)-pyridinium iodide* (4-PS)**

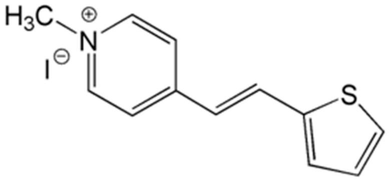

The compound was obtained as dark yellow crystals; $C_{12}H_{12}INS$; yield: 55 %; 329.2 g/mol; mp 235-237 °C lit. 232-233 °C [3]

^{1}H NMR (400 MHz, DMSO-d_6) δ (ppm): 8.84-8.84 (d, *J*=8.0 Hz, 2H), 8.27-8.23 (d, *J*=16.0 Hz, 1H), 8.21-8.19 (d, *J*=8.0 Hz, 2H), 7.53-7.52 (d, *J*=4.0 Hz, 1H), 7.22-7.20 (d, *J*=8.0 Hz, 1H),7.21 (t, 1H), 7.21-9.17 (d, *J*=16.0 Hz, 1H), 4.24 (s, 3H, CH_3);

^{13}C{^{1}H} NMR (100 MHz, DMSO-d_6) δ (ppm): 152.7 (C), 145.4 (CH), 140.9 (C), 134.2 (CH), 132.1 (CH), 130.8 (CH), 129.3 (CH), 123.6 (CH), 122.3 (CH), 47.3 (CH_3);

IR (KBr) $\tilde{\nu}$ (cm^{-1}): 3066 (-CH in thiophene), 1544, 1062, 714 (vibrations of the thiophene ring), 2978, 1594, 1207, 1092, 856 (-CH in pyridine), 1636, 1497 (vibrations of the pyridine ring), 1667 (C=N), 2905 (NCH_3), 980 (=CH).

***1-methyl-2-[2-(1-methyl-1H-pyrrol-2-yl)vinyl]pyridinium iodide* (2-PN)**

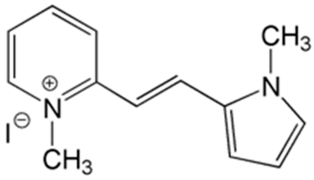

The compound was obtained as dark yellow crystals; $C_{12}H_{15}IN_2$; yield: 87 %; 326.2 g/mol; mp 249-251°C lit. 226-228 °C [2] and 187-188 °C as $CF_3SO_3^-$ [5]

^{1}H NMR (400 MHz, DMSO-d_6) δ (ppm): 8.76-8.75 (d, *J*=4.0 Hz, 1H), 8.59-8.57 (d, *J*=8.0 Hz, 1H), 8.36 (t, 1H), 7.92-7.88 (d, *J*=16.0 Hz, 1H), 7.71 (m, 1H), 7.14-7.10 (d, *J*=16.0 Hz, 1H), 7.15 (m, 2H), 4.27 (s, 3H, CH_3), 3.83 (s, 3H, CH_3);

^{13}C{^{1}H} NMR (100 MHz, DMSO-d_6) δ (ppm): 153.5 (C), 145.8 (CH), 143.6 (CH), 132.0 (CH), 130.8 (C), 129.6 (CH), 124.2 (CH), 123.5 (CH), 113.9 (CH), 111.1 (CH), 110.4 (CH), 46.1 (CH_3), 34.5 (CH_3);

IR (KBr) $\tilde{\nu}$ (cm^{-1}): 3299 (-CH in *N*-methylpyrrole), 1632, 1533, 1507 (vibrations of the *N*-methylpyrrole ring), 3075, 1591, 1204, 1110, 1078, 887, 850, 773 (-CH in pyridine), 1479 (vibrations of the pyridine ring), 1658 (C=N), 2977 (NCH_3), 959 (=CH).

1-methyl-4-[2-(1-methyl-1H-pyrrol-2-yl)vinyl]pyridinium iodide (**4-PN**)

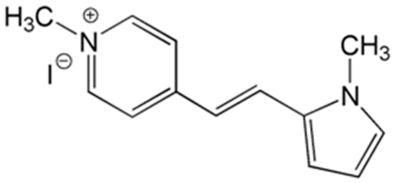

The compound was obtained as dark yellow crystals; $C_{12}H_{15}IN_2$; yield: 65 %; 326.2 g/mol; mp 248-250 ℃ lit. 252-254 ℃ [6]

^{1}H NMR (400 MHz, DMSO-*d*$_6$) δ (ppm): 8.71-8.69 (d, *J*=8.0 Hz, 2H), 8.12-8.10 (d, *J*=8.0 Hz, 1H), 7.93-7.89 (d, *J*=16.0 Hz, 2H), 7.10 (m, 1H), 7.09-7.05 (d, *J*=16.0 Hz, 1H), 6.88 (t, 1H), 6.21 (m, 1H), 4.18 (s, 3H, CH_3), 3.81 (s, 3H, CH_3);

^{13}C{^{1}H} NMR (100 MHz, DMSO-*d*$_6$) δ (ppm): 153.7 (C), 144.7 (CH), 130.9 (C), 129.9 (CH), 129.1 (CH), 122.6 (CH), 117.9 (CH), 112.8 (CH), 110.4 (CH), 46.9 (CH_3), 34.6 (CH_3);

IR (KBr) $\tilde{\nu}$ (cm^{-1}): 3249 (-CH in *N*-methylpyrrole), 1630, 1536, 1506 (vibrations of the *N*-methylpyrrole ring), 3090, 1586, 1213, 1122, 1084, 882, 822, 747 (-CH in pyridine), 1456 (vibrations of the pyridine ring), 1667 (C=N), 2989 (NCH_3), 972 (=CH).

2-(2-furan-2-yl-vinyl)-1-methyl-quinolinium iodide (**2-QO**)

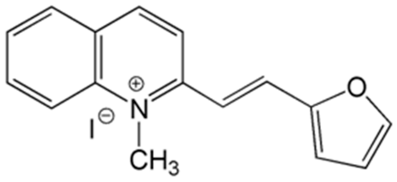

The crude product was purified by silica gel column chromatography eluted with dichloromethane/methanol/acetone (10:5:2 V/V/V). The compound was obtained as dark orange fine crystalline powder; $C_{16}H_{14}INO$; 363.2 g/mol; yield: 29.5 %; mp 230-232 ℃ lit. 226.2 ℃ [7]

^{1}H NMR (400 MHz, DMSO-*d*$_6$) δ (ppm): 9.05-9.03 (d, *J*=8.0 Hz, 1H), 8.55 (m, 2H), 8.36-8.34 (d, *J*=8.0 Hz, 1H), 8.18 (m, 2H), 8.13-8.09 (d, *J*=16.0 Hz, 1H), 7.95 (t, 2H), 7.59-7.55 (d, *J*=16.0 Hz, 1H), 7.26-7.25 (d, *J*=4.0 Hz, 1H), 6.83-6.82 (d, *J*=4.0 Hz, 1H), 4.52 (s, 3H, CH_3);

^{13}C{^{1}H} NMR (100 MHz, DMSO-*d*$_6$) δ (ppm): 156.1.0 (C), 151.8 (CH), 148.1 (CH), 144.3 (C), 139.7 (C), 135.4 (CH), 133.3 (CH), 130.5 (CH), 129.4 (CH), 128.1 (C), 121.2 (CH), 119.7 (CH), 118.9 (CH), 116.6 (CH), 114.3 (CH), 40.5 (CH_3);

IR (KBr) $\tilde{\nu}$ (cm^{-1}): 3120 (-CH in furan), 1547, 767 (vibrations of the furan ring), 3065, 1602, 1228, 1189, 1087, 1034, 894, 848 (-CH in quinoline), 1483 (vibrations of the quinoline ring), 2904 (NCH_3), 972 (=CH).

4-(2-furan-2-yl-vinyl)-1-methyl-quinolinium iodide (**4-QO**)

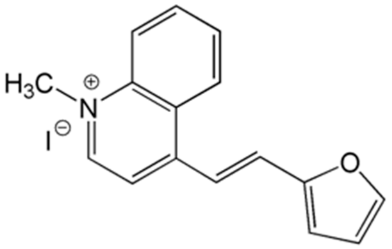

The crude product was purified by column chromatography on silica gel eluting with dichloromethane/methanol/acetone (10:5:2, V/V). The compound was obtained as dark yellow powder; $C_{16}H_{14}INO$; 363.2 g/mol; yield: 14 %; mp 238-240 °C.

^1H NMR (400 MHz, DMSO-d_6) δ (ppm): 9.33-9.31 (d, *J*=8.0 Hz, 1H), 8.89-8.87 (d, *J*=8.0 Hz, 1H), 8.45 (t, 2H), 8.27 (t, 1H), 8.11-8.07 (d, *J*=16.0 Hz, 1H), 8.03 (m, 2H), 8.00-7.96 (d, *J*=16.0 Hz, 1H), 7.20-7.19 (d, *J*=4.0 Hz, 1H), 6.79-6.78 (d, *J*=4.0 Hz, 1H), 4.53 (s, 3H, CH_3);

$^{13}C\{^1H\}$ NMR (100 MHz, DMSO-d_6) δ (ppm): 152.4 (C), 152.3 (C), 148.4 (CH), 147.2 (CH), 139.2 (C), 135.5 (CH), 129.9 (C), 129.8 (CH), 126.5 (CH), 119.9 (CH), 117.4 (CH), 117.0 (CH), 116.3 (CH), 114.0 (CH), 45.1 (CH_3);

IR (KBr) $\tilde{\nu}$ (cm^{-1}): 3127 (-CH in furan), 1544, 1034, 757 (vibrations of the furan ring), 3069, 1595, 1212, 1095, 1034, 894 (-CH in quinoline), 1493 (vibrations of the quinoline ring), 1646 (C=N), 2910 (NCH_3), 993 (=CH).

***1-methyl-2-(2-thiophen-2-yl-vinyl)-qinolinium iodide* (2-QS)**

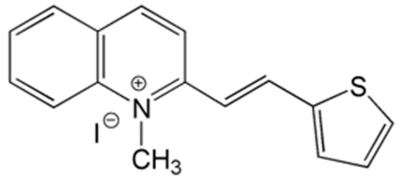

The compound was obtained as dark orange needles; $C_{16}H_{14}INS$; 379.3 g/mol; yield: 48.5 %; mp 236-237 °C.

^1H NMR (400 MHz, DMSO-d_6) δ (ppm): 9.06-9.04 (d, *J*=8.0 Hz, 1H), 8.57-8.55 (d, *J*=8.0 Hz, 2H), 8.49-8.45 (d, *J*=16.0 Hz, 1H), 8.36-8.34 (dd, *J*=8.0 Hz, 1H), 8.18 (tt, 1H), 7.98 (m, 1H), 7.95-7.93 (d, *J*=8.0 Hz, 1H), 7.85-7.84 (d, *J*=4.0 Hz, 1H), 7.64-7.60 (d, *J*=16.0 Hz, 1H), 7.31 (t, 1H), 4.53 (s, 3H, CH_3);

$^{13}C\{^1H\}$ NMR (100 MHz, DMSO-d_6) δ (ppm): 156.2 (C), 144.3 (CH), 140.6 (C), 140.1 (CH), 139.7 (C), 135.3 (CH), 133.7 (CH), 133.2 (CH), 130.5 (CH), 129.6 (CH), 129.4 (CH), 128.1 (C), 121.3 (CH), 119.8 (CH), 117.9 (CH), 40.5 (CH_3);

IR (KBr) $\tilde{\nu}$ (cm^{-1}): 3103 (-CH in thiophene), 1543, 1030, 713 (vibrations of the thiophene ring), 3047, 1596, 1183, 1068, 856, 784, 713 (-CH in quinoline), 1624, 1498 (vibrations of the quinoline ring), 982 (=CH).

***1-methyl-4-(2-thiophen-2-yl-vinyl)-quinolinium iodide* (4-QS)**

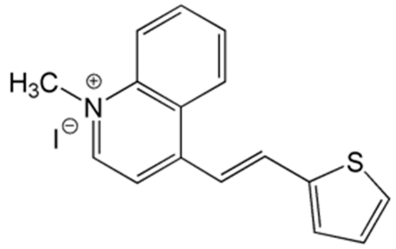

The compound was obtained as dark yellow powder; $C_{16}H_{14}INS$; 379.3 g/mol; yield: 57 %; mp 268-270 °C lit. 248-251 °C [8]

^1H NMR (400 MHz, DMSO-d_6) δ (ppm): 9.34-9.32 (d, *J*=8.0 Hz, 1H), 8.96-8.94 (d, *J*=8.0 Hz, 2H), 8.47-8.45 (d, *J*=8.0 Hz, 2H), 8.43-8.39 (d, *J*=16.0 Hz, 1H), 8.27 (tt, 1H), 7.98 (m, 1H), 8.06-8.04 (dd, *J*=8.0 Hz, 1H), 8.03-7.99 (d, *J*=16.0 Hz, 1H), 7.91-7.89 (d, *J*=8.0 Hz, 1H), 7.80-7.79 (d, *J*=4.0 Hz, 1H), 7.28 (tt, 1H), 4.54 (s, 3H, CH_3);

$^{13}C\{^1H\}$ NMR (100 MHz, DMSO-d_6) δ (ppm): 152.6 (C), 148.4 (C), 140.2 (CH), 139.2 (CH), 136.2 (C), 135.6 (CH), 132.4 (C), 131.8 (CH), 129.7 (CH), 129.4 (C), 126.8 (CH), 126.5 (CH), 119.8 (CH), 118.7 (CH), 116.3 (CH), 45.1 (CH_3);

IR (KBr) ṽ (cm^{-1}): 3099 (-CH in thiophene), 1560, 1063, 751 (vibrations of the thiophene ring), 3044, 1599, 1198, 1110, 839, 792 (-CH in quinoline), 1623, 1445 (vibrations of the quinoline ring), 2988 (NCH_3), 985 (=CH).

1-methyl-2-[2-(1-methyl-1H-pyrrol-2-yl)vinyl]-quinolinium iodide (**2-QN**)

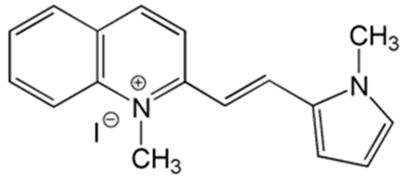

The compound was obtained as red fine crystalline powder; $C_{17}H_{17}IN_2$; 376.2 g/mol; yield: 58 %; mp 264-266 °C.
^{1}H NMR (400 MHz, DMSO-d_6) δ (ppm): 8.84-8.82 (d, *J*=8.0 Hz, 1H), 8.66-8.64 (d, *J*=8.0 Hz, 1H), 8.44-8.42 (d, *J*=8.0 Hz, 1H), 8.26-8.24 (d, *J*=8.0 Hz, 1H), 8.21-8.17 (dd, *J*=8.0 Hz, 1H), 8.09 (tt, 1H), 7.85 (t, 1H), 7.49-7.45 (d, *J*=16.0 Hz, 1H), 7.40 (m, 1H), 7.30 (m, 1H), 6.34 (m, 1H), 4.43 (s, 3H, CH_3), 3.90 (s, 3H, CH_3);
^{13}C{^{1}H} NMR (100 MHz, DMSO-d_6) δ (ppm): 156.6 (C), 142.2 (CH), 139.7 (C), 136.1 (CH), 134.6 (CH), 131.9 (C), 131.6 (CH), 130.2 (CH), 128.5 (CH), 127.3 (CH), 120.8 (CH), 119.3 (CH), 116.4 (C), 112.4 (CH), 111.5 (CH), 39.5 (CH_3), 34.7 (CH_3);
IR (KBr) ṽ (cm^{-1}): 3123 (-CH in *N*-methylpyrrole), 1617, 1543, 1511 (vibrations of the *N*-methylpyrrole ring), 3058, 1596, 1204, 1114, 1081, 887, 850, 780 (-CH in quinoline), 1491 (vibrations of the quinoline ring), 1664 (C=N), 2979 (NCH_3), 963 (=CH).

1-methyl-4-[2-(1-methyl-1H-pyrrol-2-yl)vinyl]qiunolinium iodide (**4-QN**)

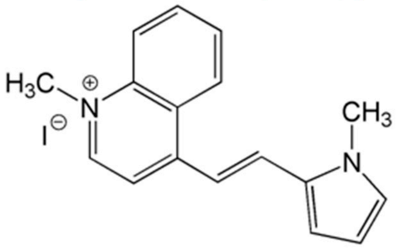

The compound was obtained as dark red fine crystalline powder; $C_{17}H_{17}IN_2$; 376.2 g/mol; yield: 63 %; mp 260-262 °C lit. 215-217°C [9]
^{1}H NMR (400 MHz, DMSO-d_6) δ (ppm): 9.13-9.11 (d, *J*=8.0 Hz, 1H), 8.97-8.95 (d, *J*=8.0 Hz, 1H), 8.49-8.47 (d, *J*=8.0 Hz, 1H), 8.36-8.34 (d, *J*=8.0 Hz, 1H), 8.21 (tt, 1H), 8.18-8.14 (d, *J*=16.0 Hz, 1H), 7.99 (t, 1H), 7.94-7.90 (d, *J*=16.0 Hz, 1H), 7.36 (m, 1H), 7.21 (m, 1H), 6.31 (m, 1H), 4.45 (s, 3H, CH_3), 3.88 (s, 3H, CH_3);
^{13}C{^{1}H} NMR (100 MHz, DMSO-d_6) δ (ppm): 153.4 (C), 147.1 (CH), 139.2 (CH), 135.1 (CH), 132.2 (C), 130.9 (CH), 130.4 (C), 129.0 (CH), 126.6 (CH), 126.1 (CH), 119.6 (CH), 114.9 (CH), 114.5 (C), 113.9 (CH), 111.0 (CH), 44.5 (CH_3), 34.5 (CH_3);
IR (KBr) ṽ (cm^{-1}): 3117 (-CH in *N*-methylpyrrole), 1617, 1552, 1507 (vibrations of the *N*-methylpyrrole ring), 3071, 1590, 1222, 1168, 1084, 876, 842, 784 (-CH in quinoline), 1466 (vibrations of the quinoline ring), 1662 (C=N), 2981 (NCH_3), 968 (=CH).

^{1}H NMR spectrum of the **2-PN**

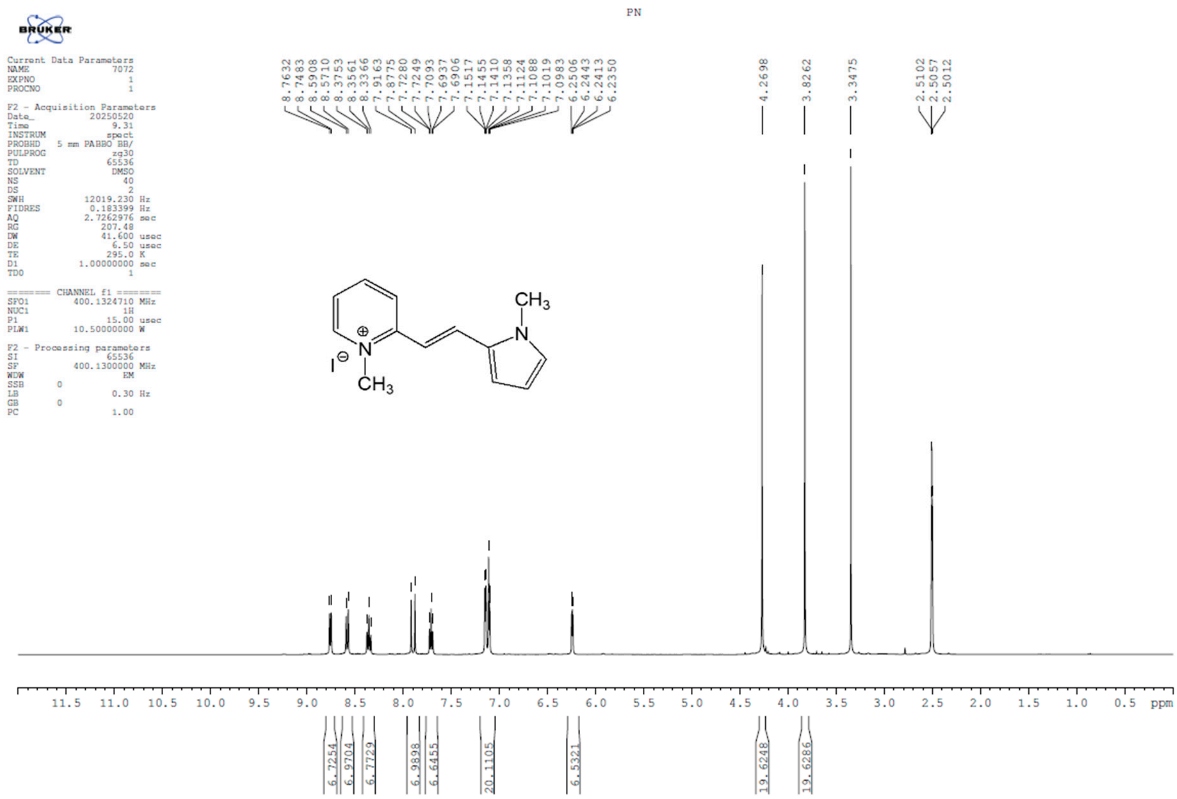

^{1}H NMR spectrum of the **4-PN**

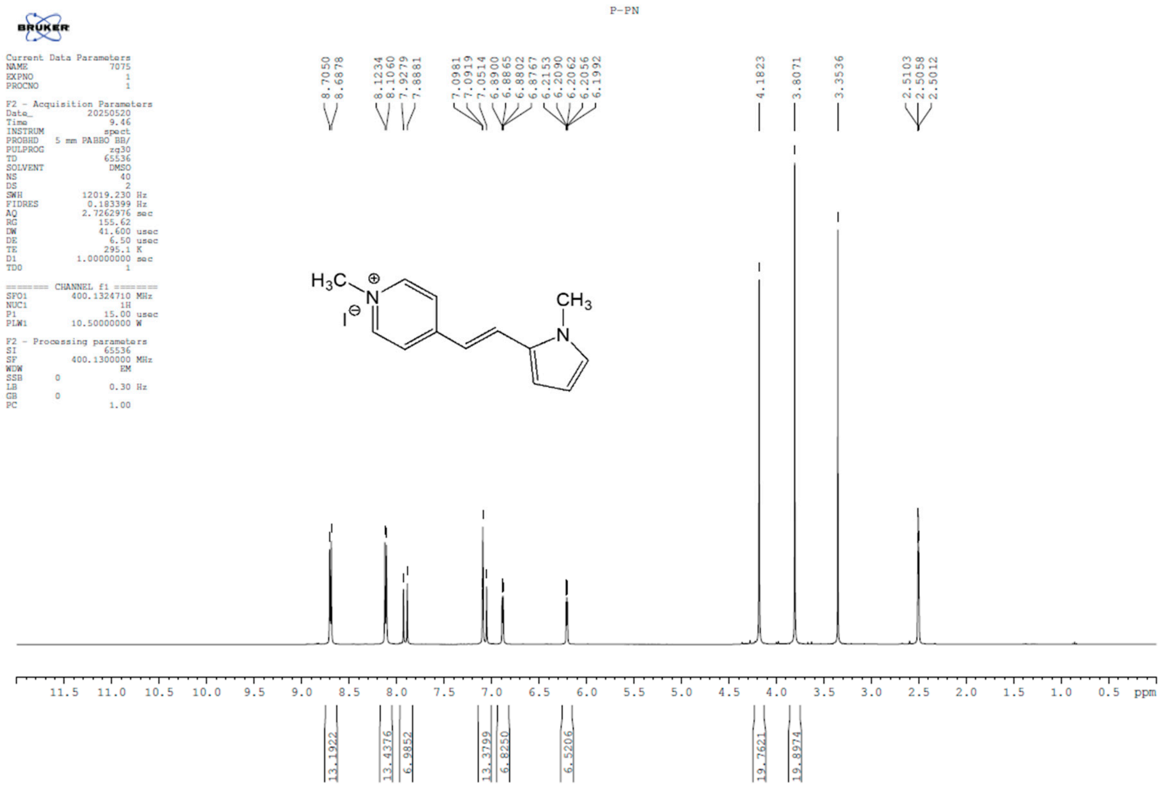

^{1}H NMR spectrum of the **2-QN**

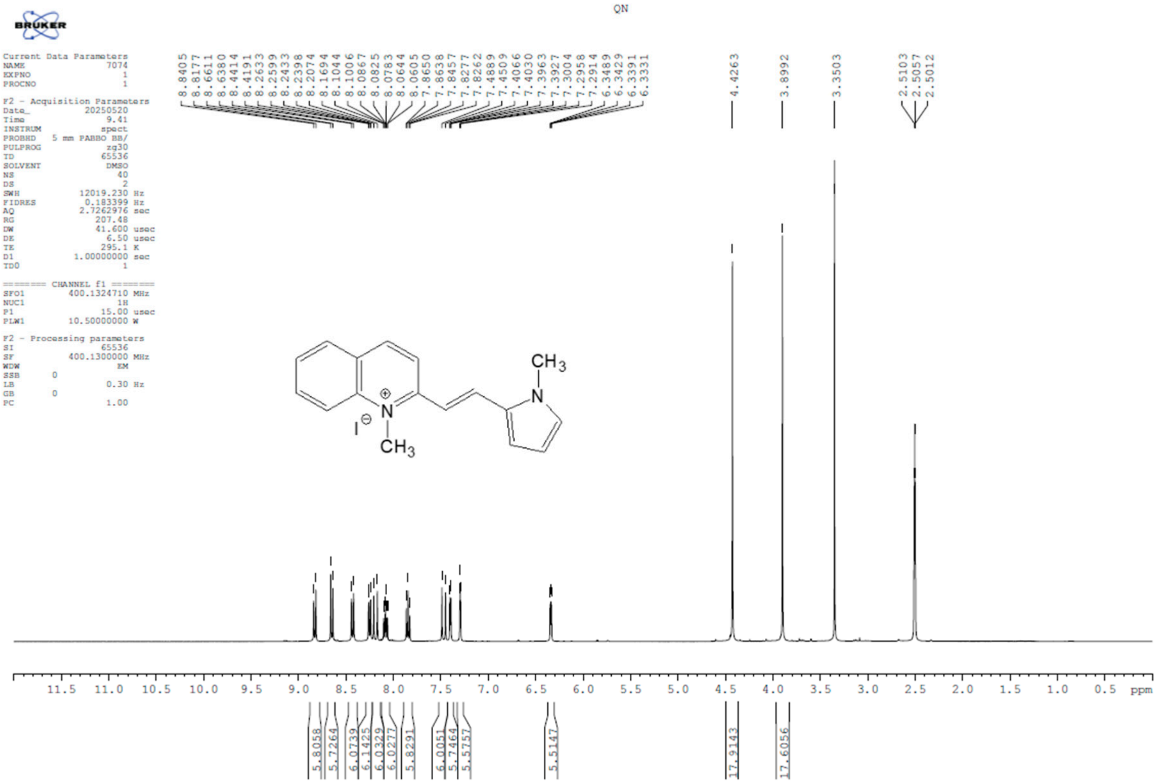

^{1}H NMR spectrum of the **4-QN**

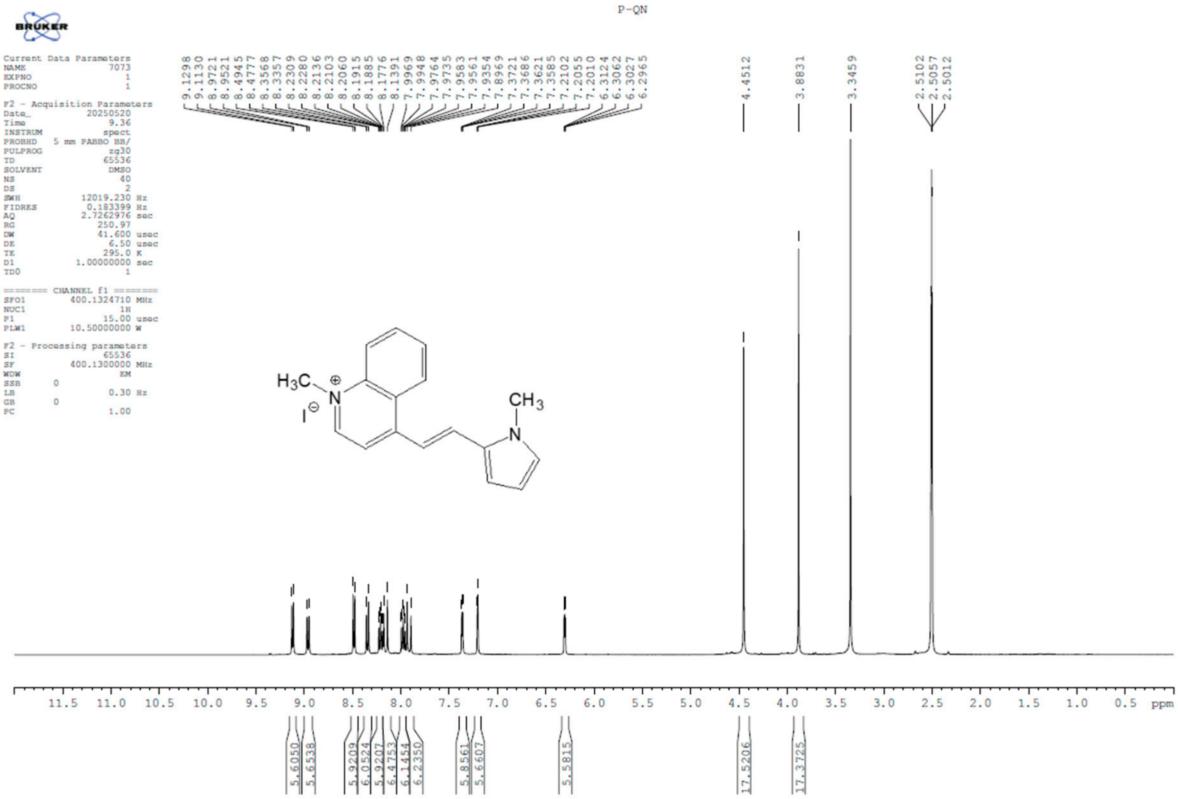

^{1}H NMR spectrum of the **2-PS**

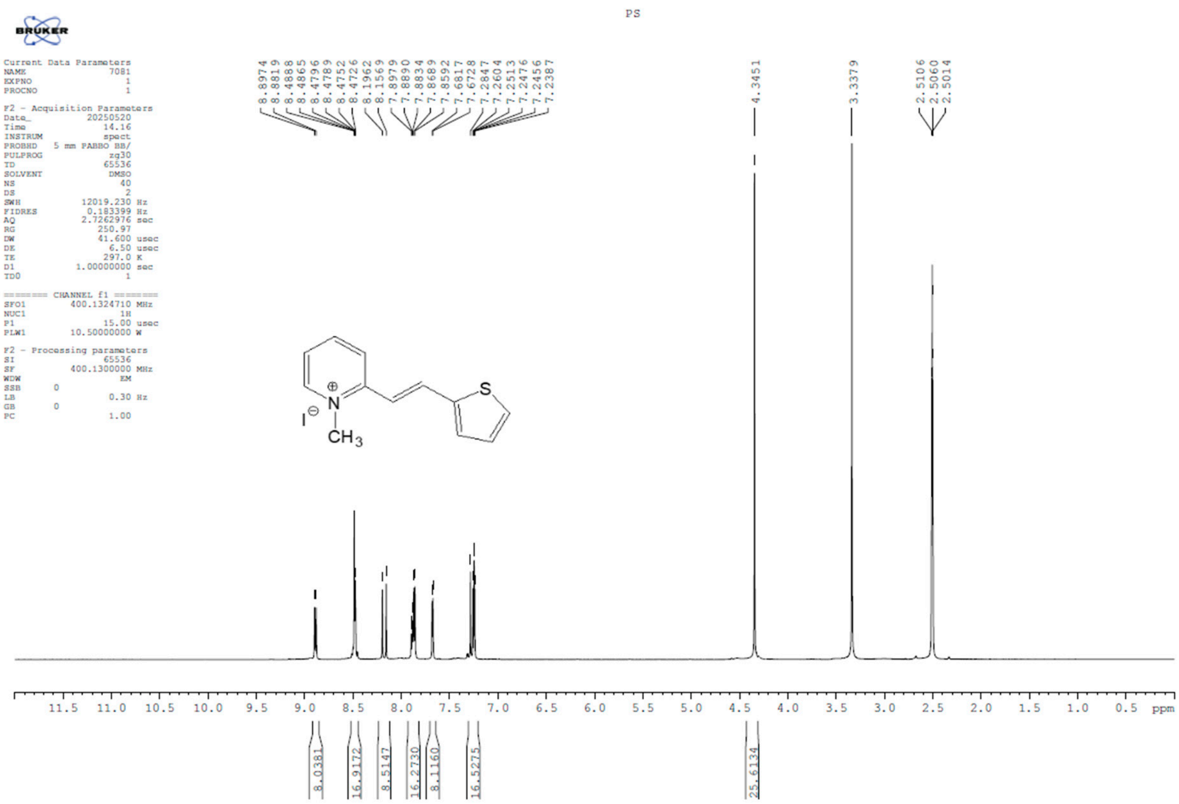

^{1}H NMR spectrum of the **4-PS**

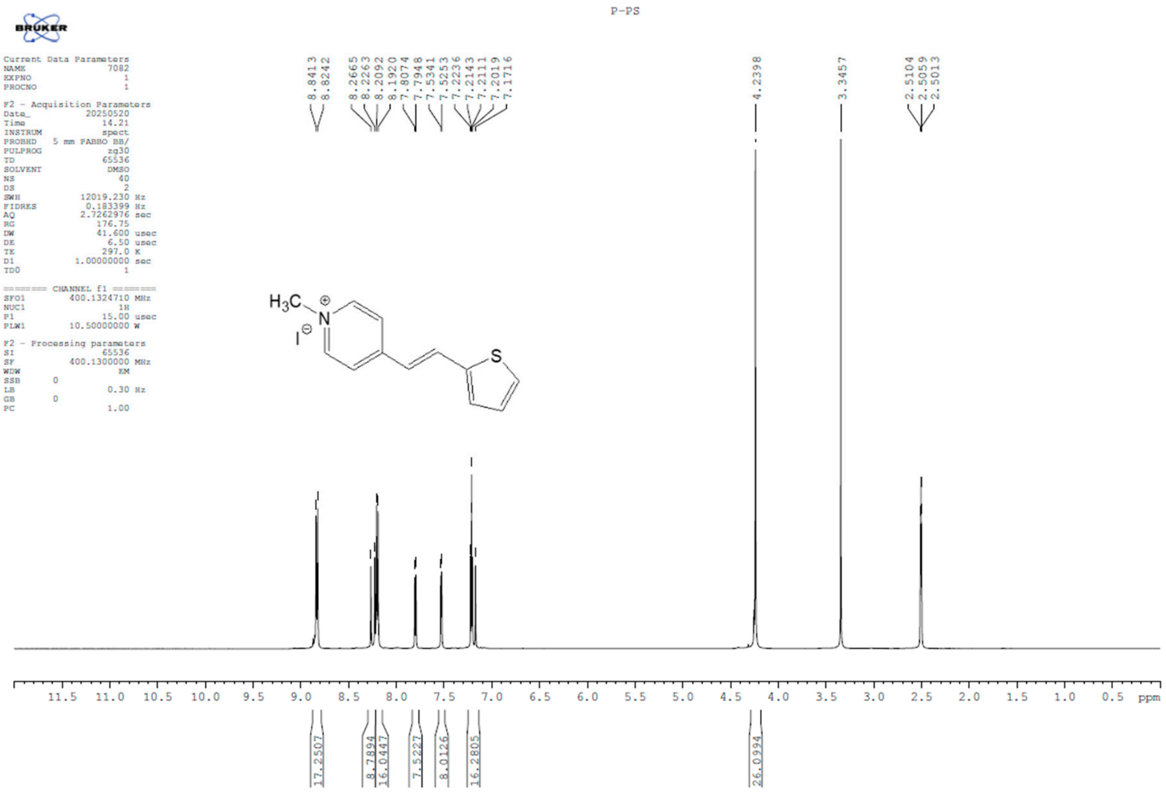

^{1}H NMR spectrum of the **2-QS**

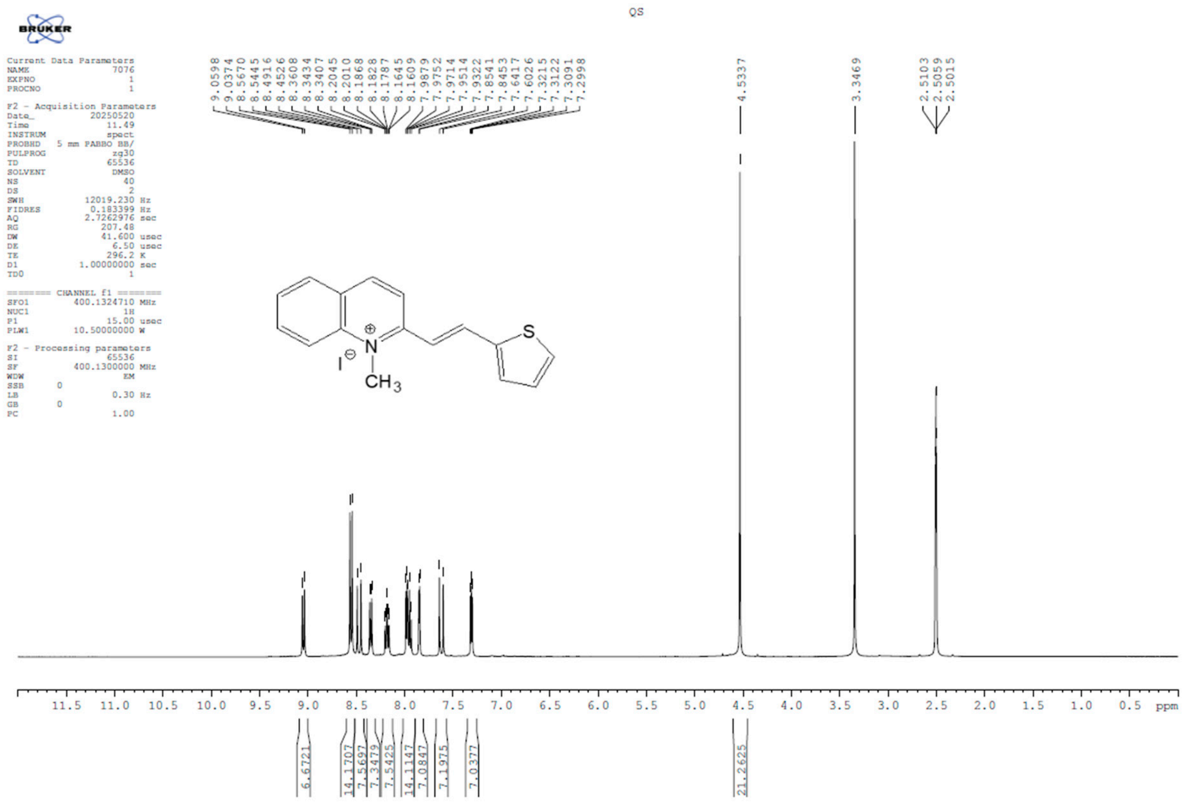

^{1}H NMR spectrum of the **4-QS**

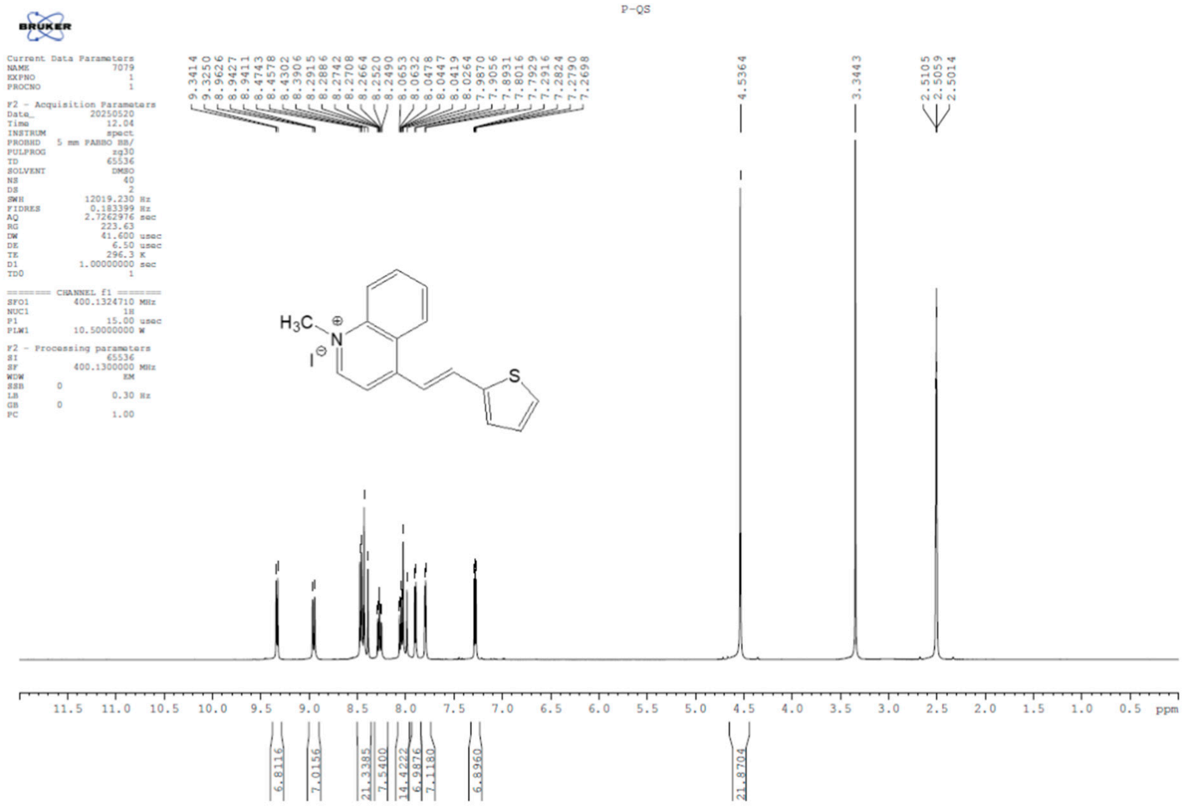

^{1}H NMR spectrum of the **2-PO**

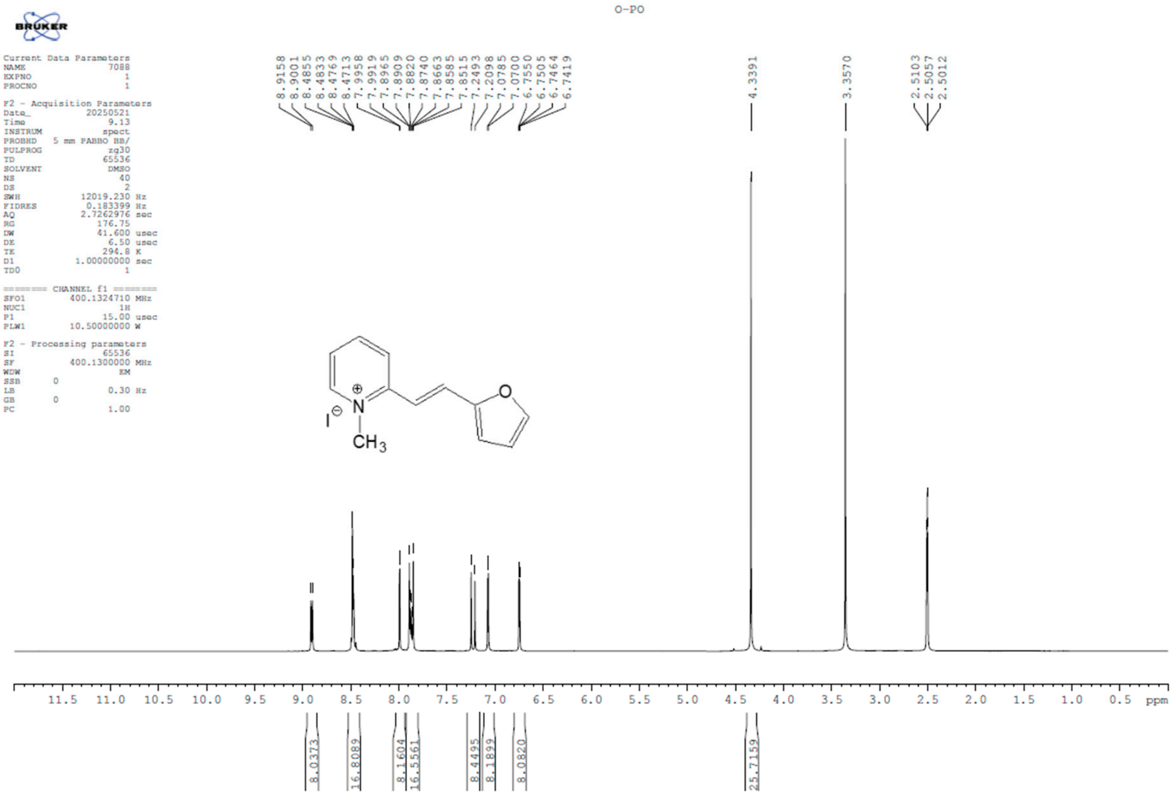

^{1}H NMR spectrum of the **4-PO**

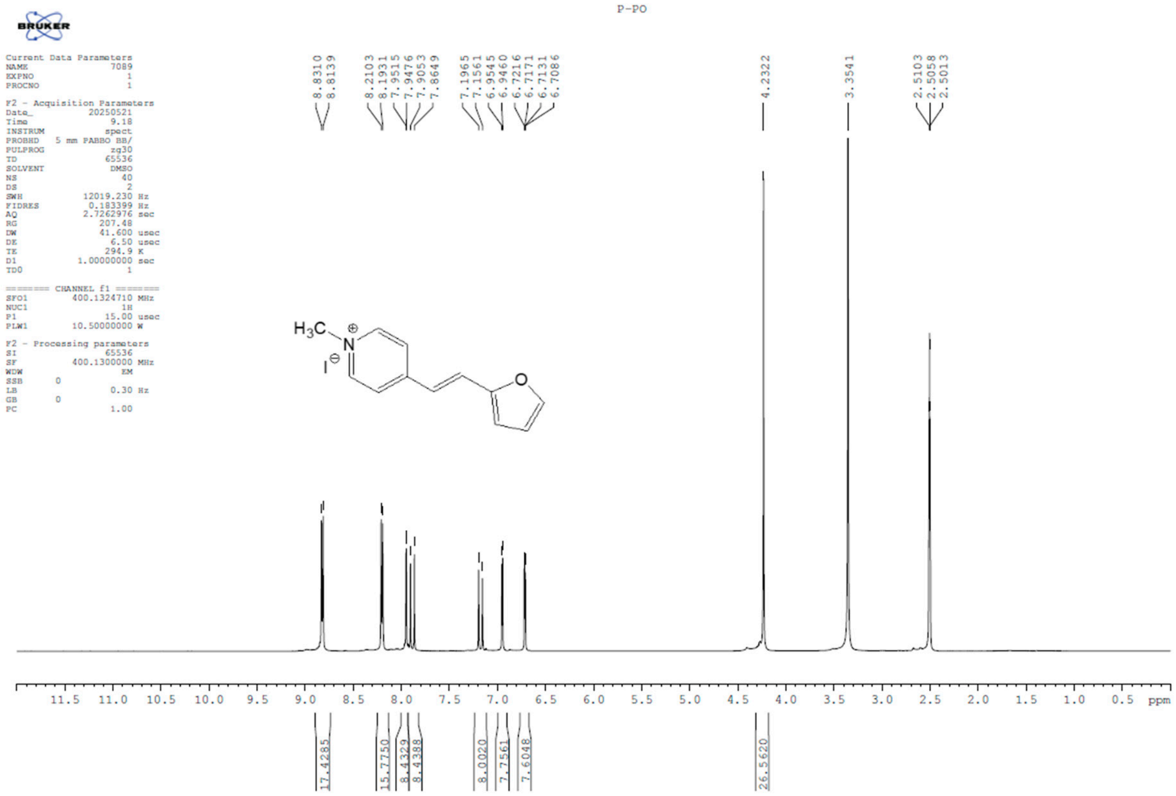

^{1}H NMR spectrum of the **2-QO**

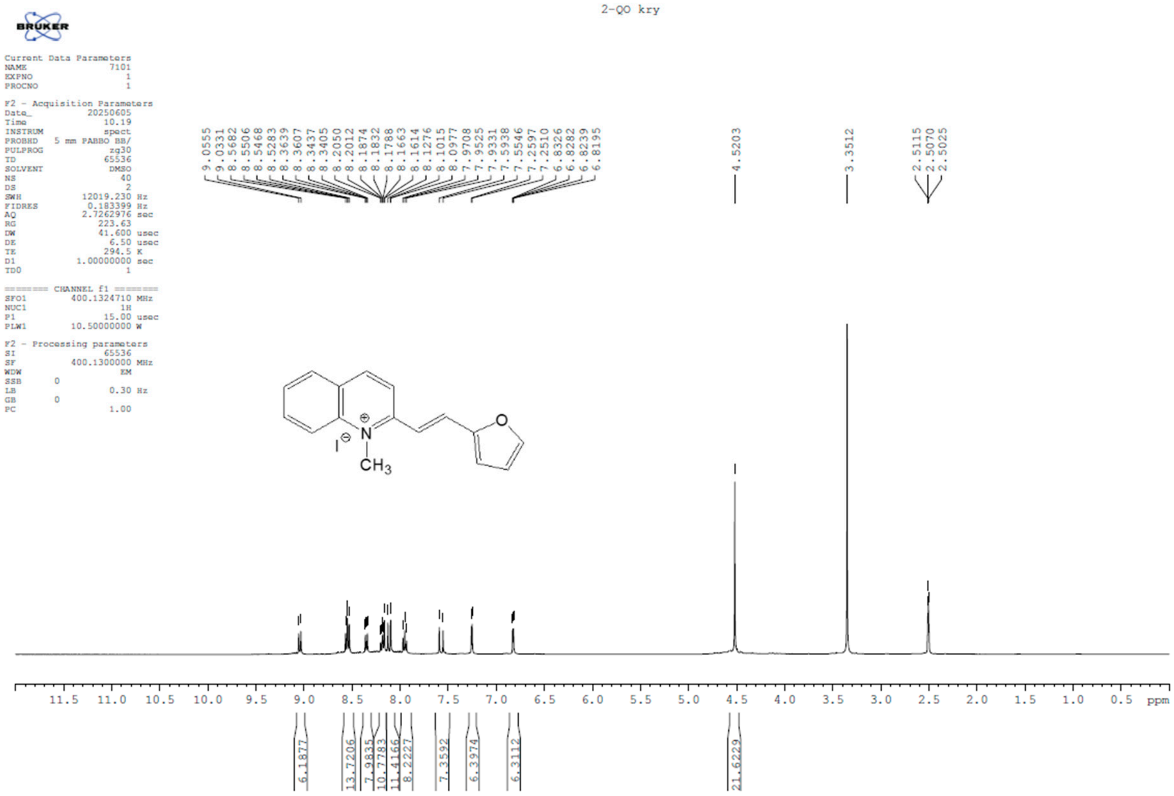

^{1}H NMR spectrum of the **4-QO**

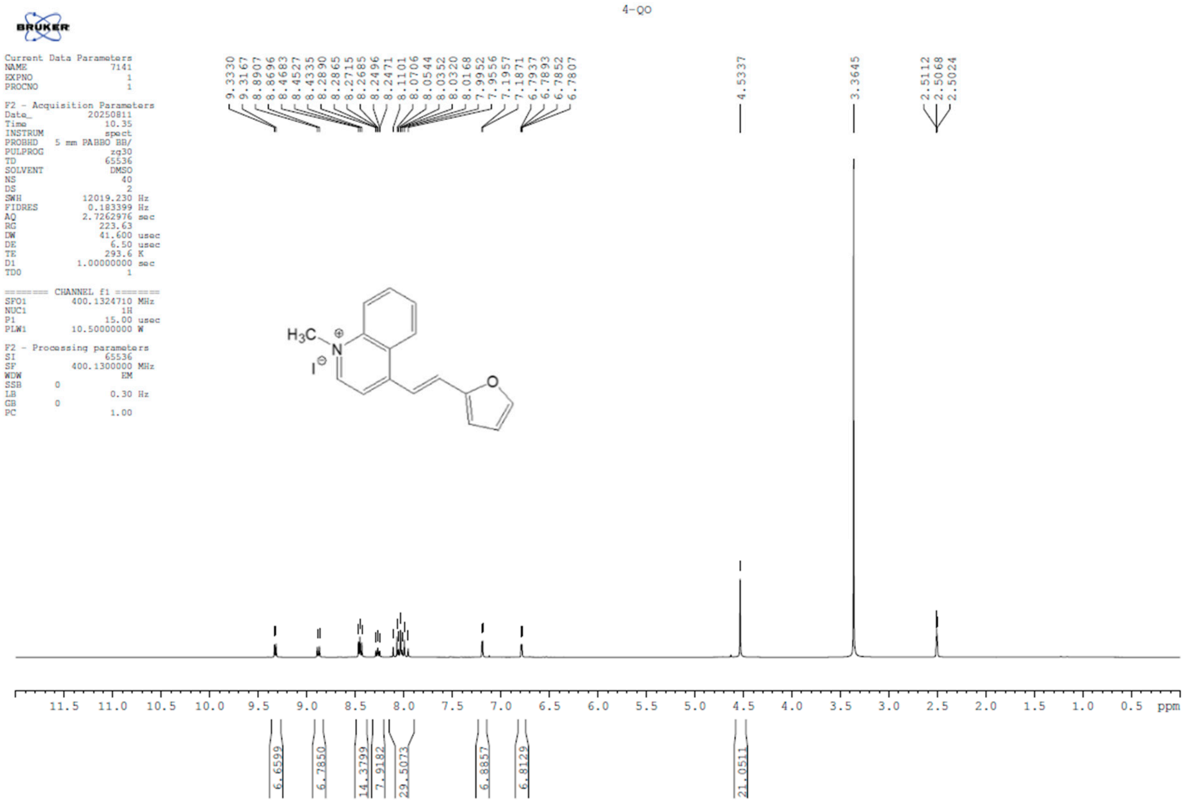

^{13}C NMR spectrum of the **2-PN**

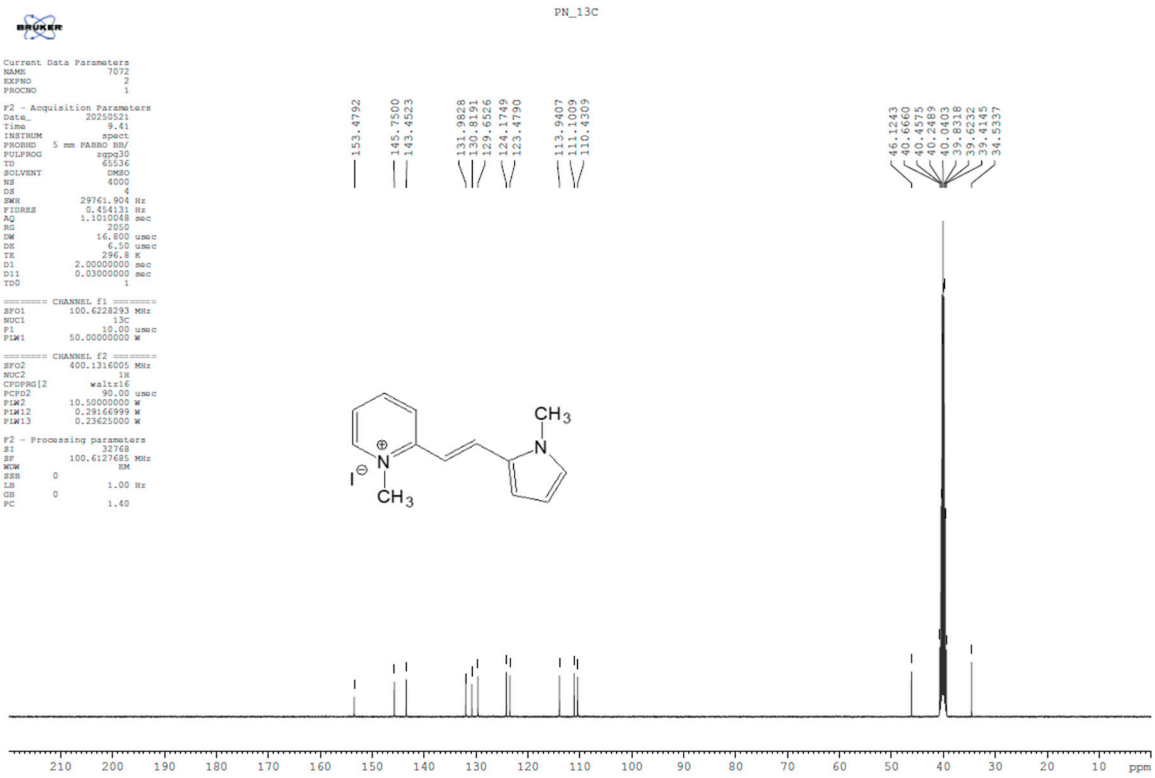

^{13}C NMR spectrum of the **4-PN**

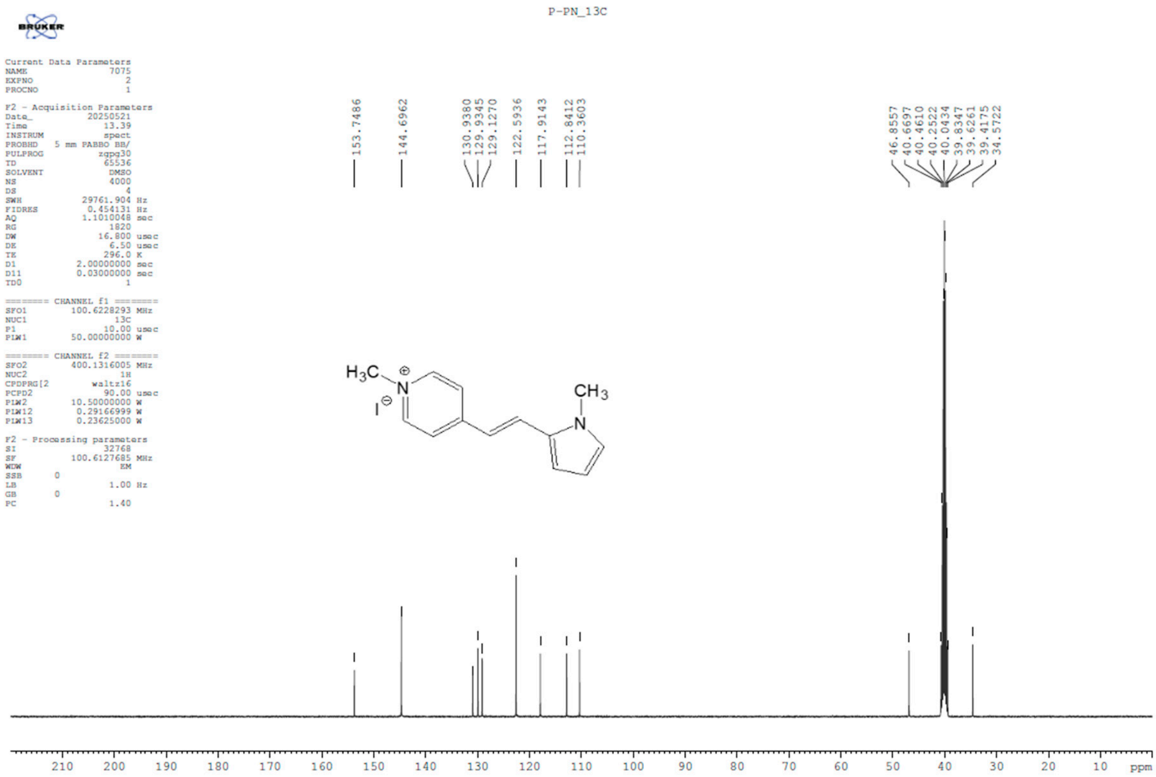

^{13}C NMR spectrum of the **2-QN**

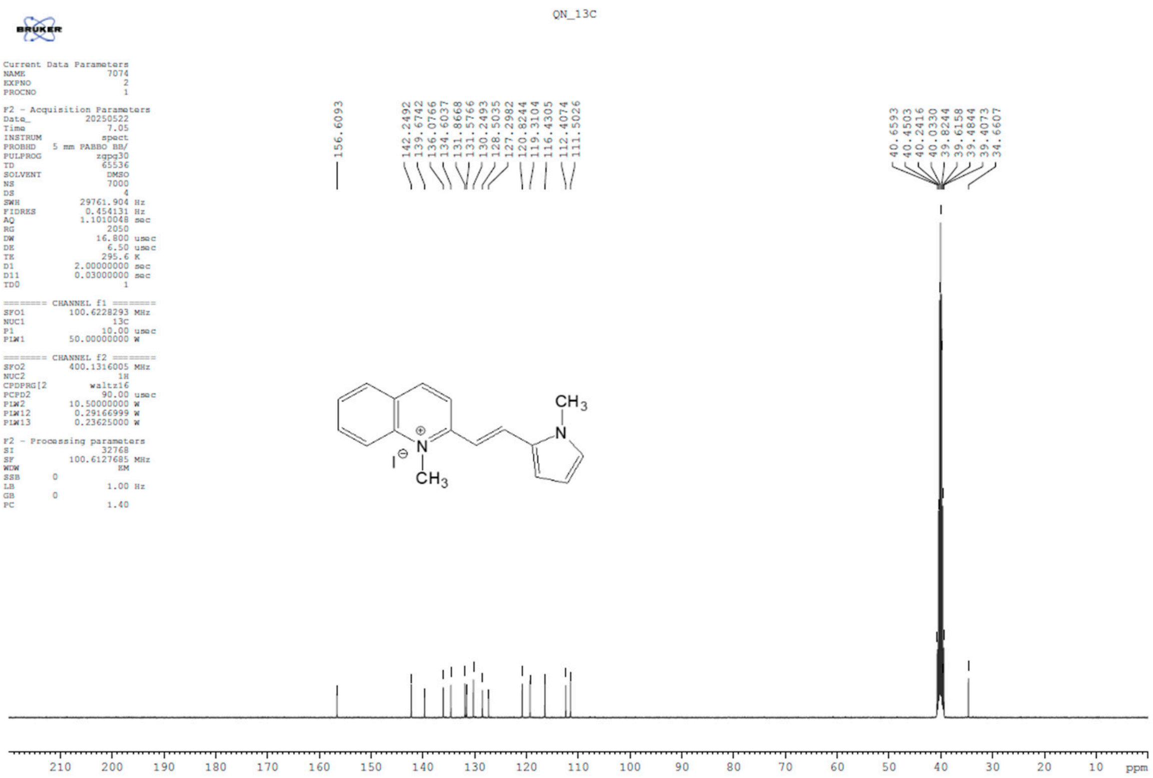

^{13}C NMR spectrum of the **4-QN**

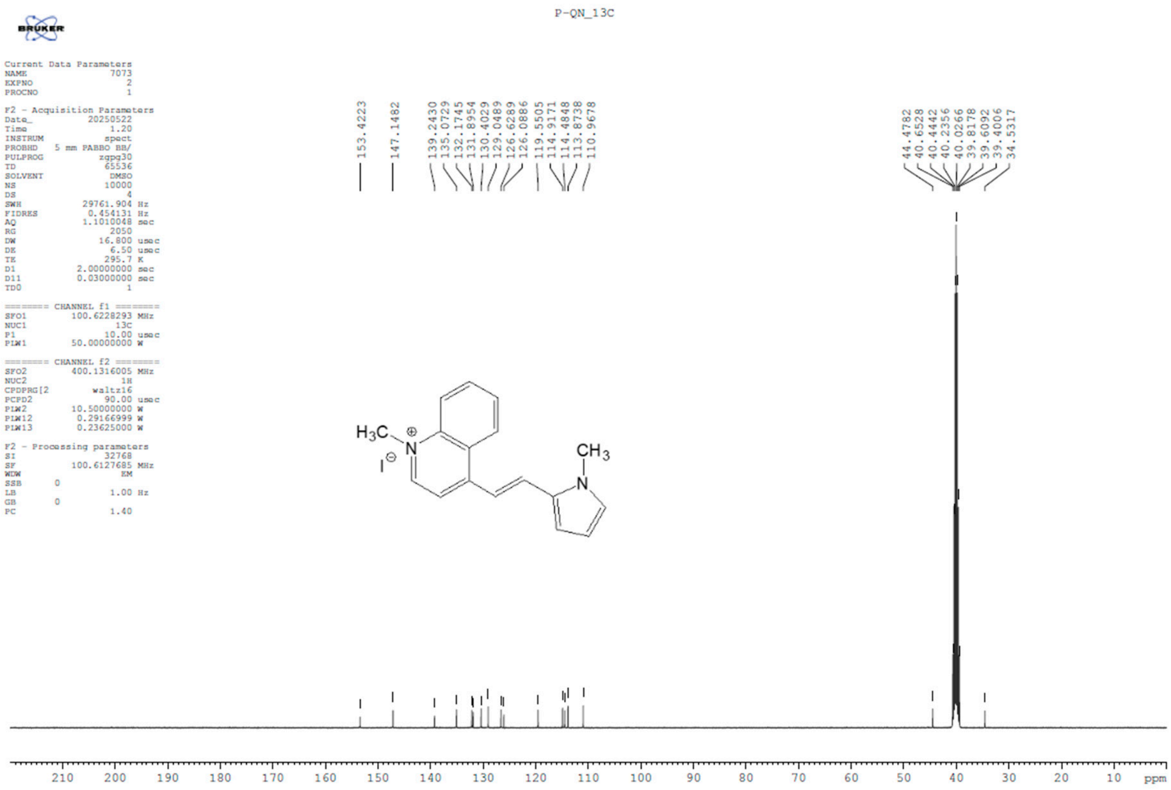

^{13}C NMR spectrum of the **2-PS**

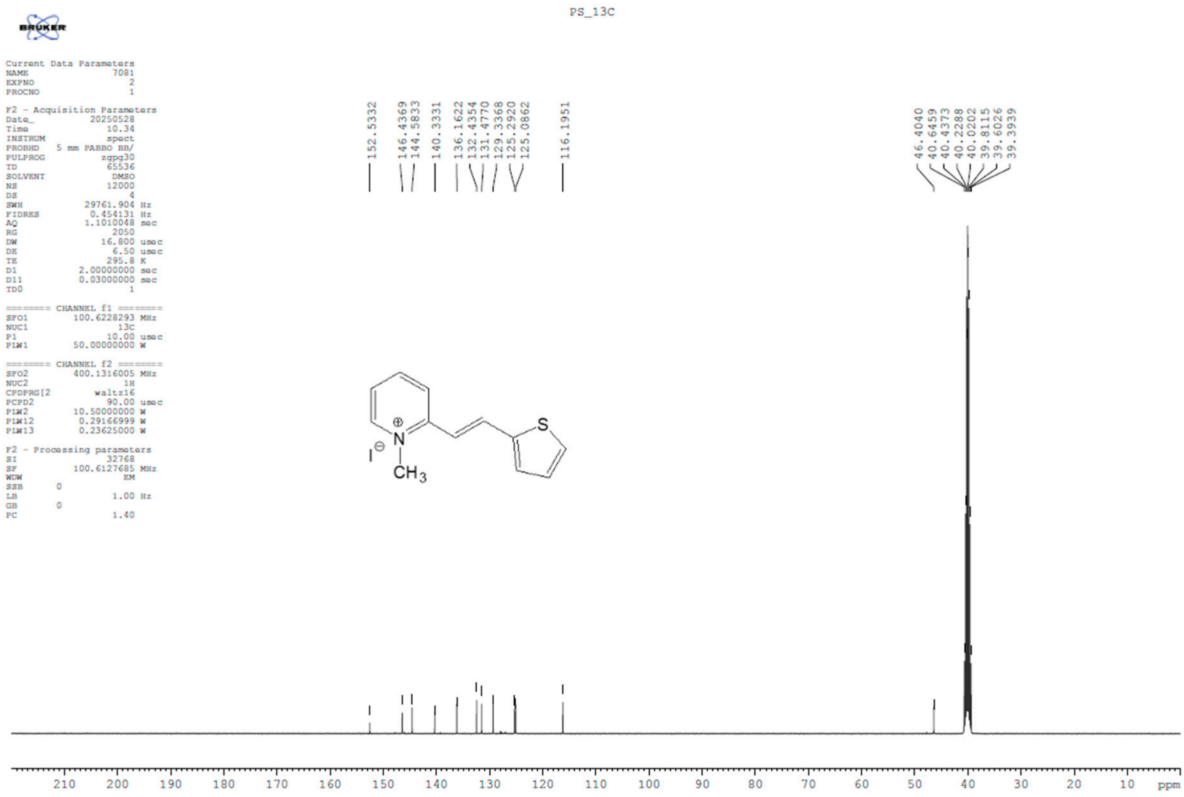

^{13}C NMR spectrum of the **4-PS**

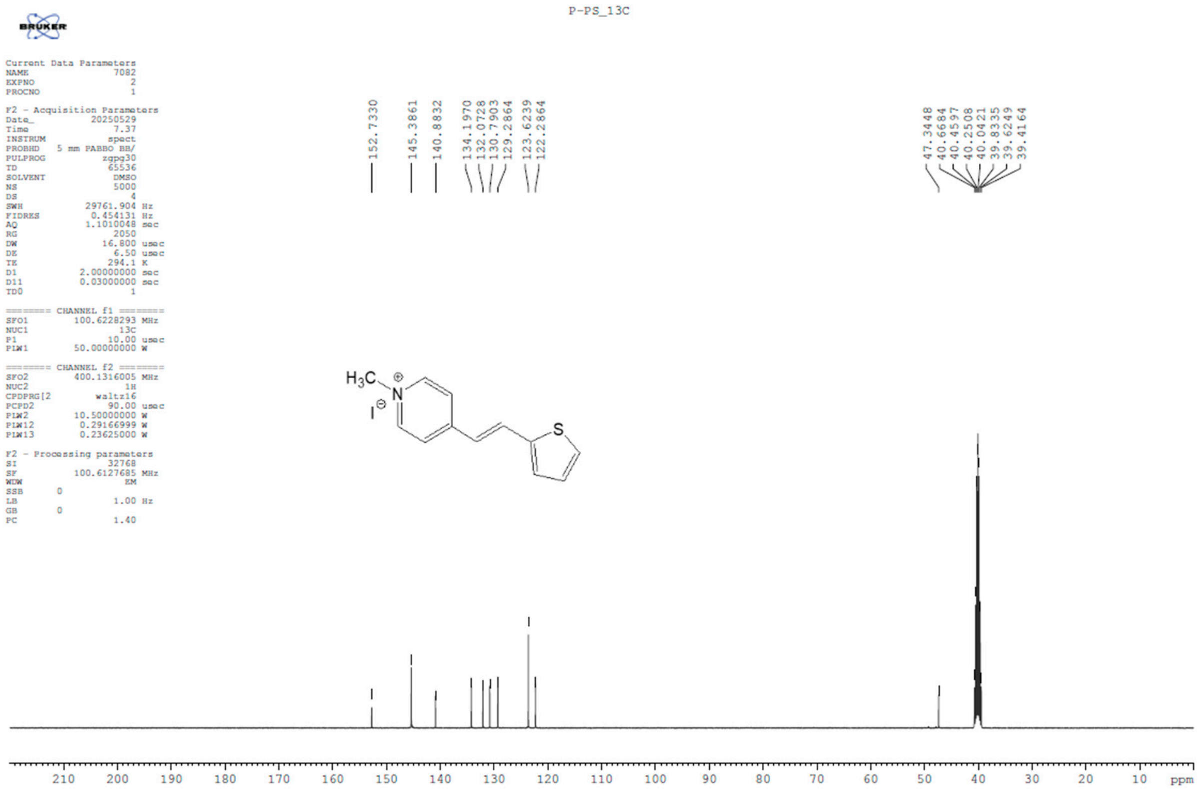

^{13}C NMR spectrum of the **2-QS**

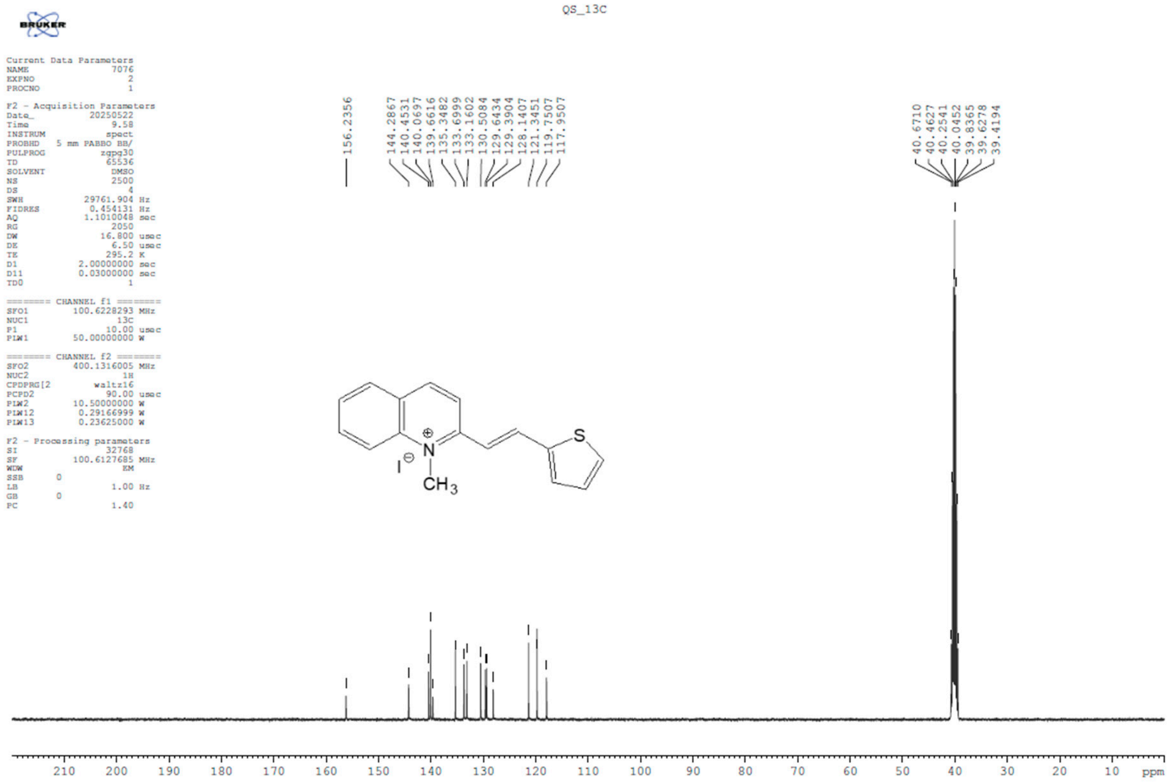

^{13}C NMR spectrum of the **4-QS**

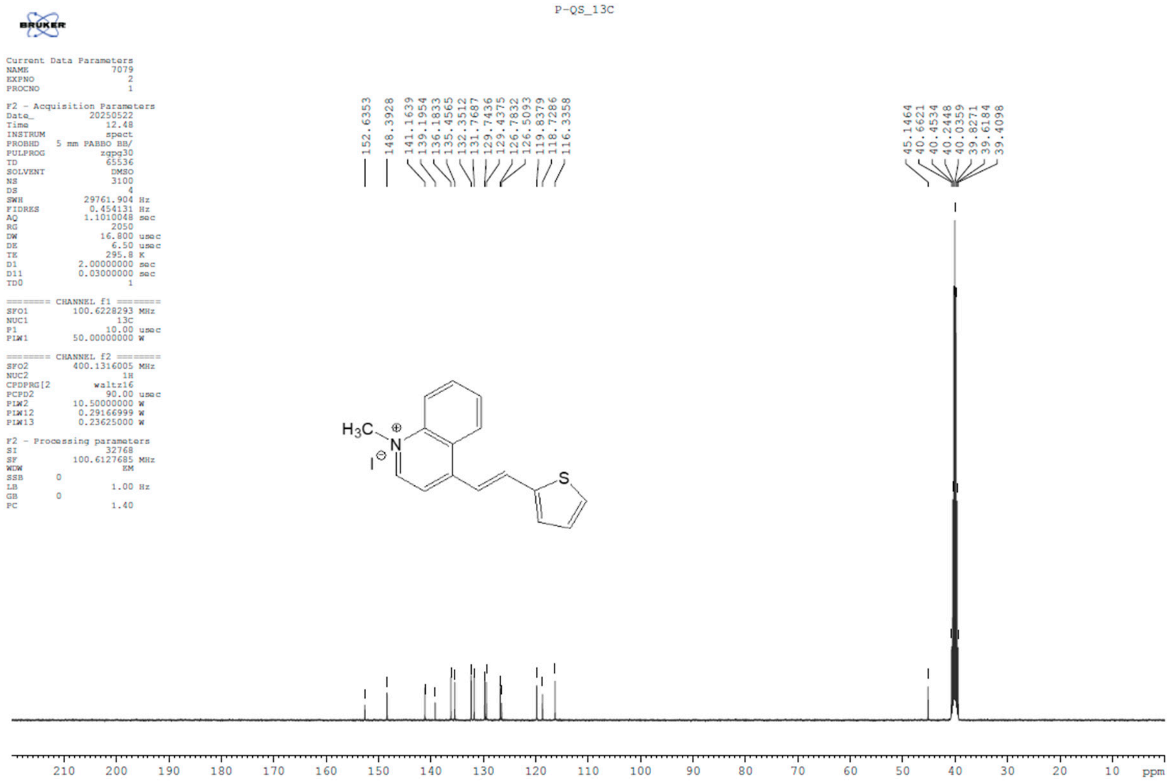

^{13}C NMR spectrum of the **2-PO**

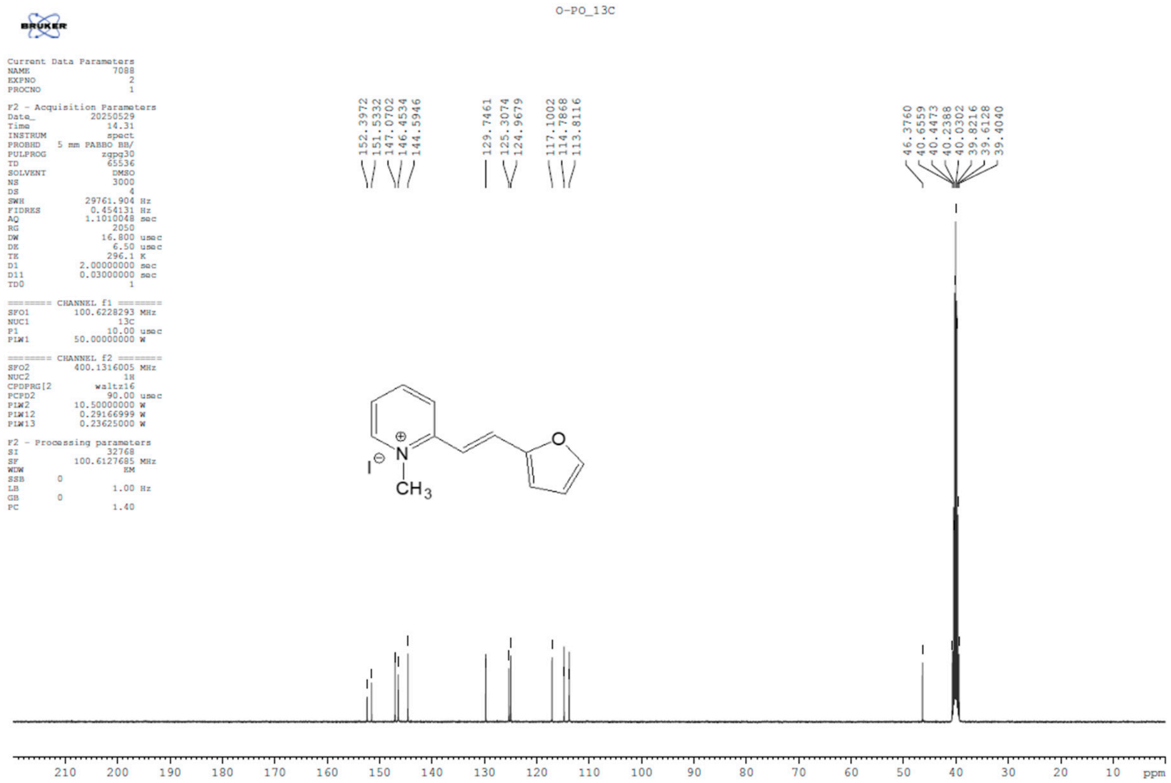

^{13}C NMR spectrum of the **4-PO**

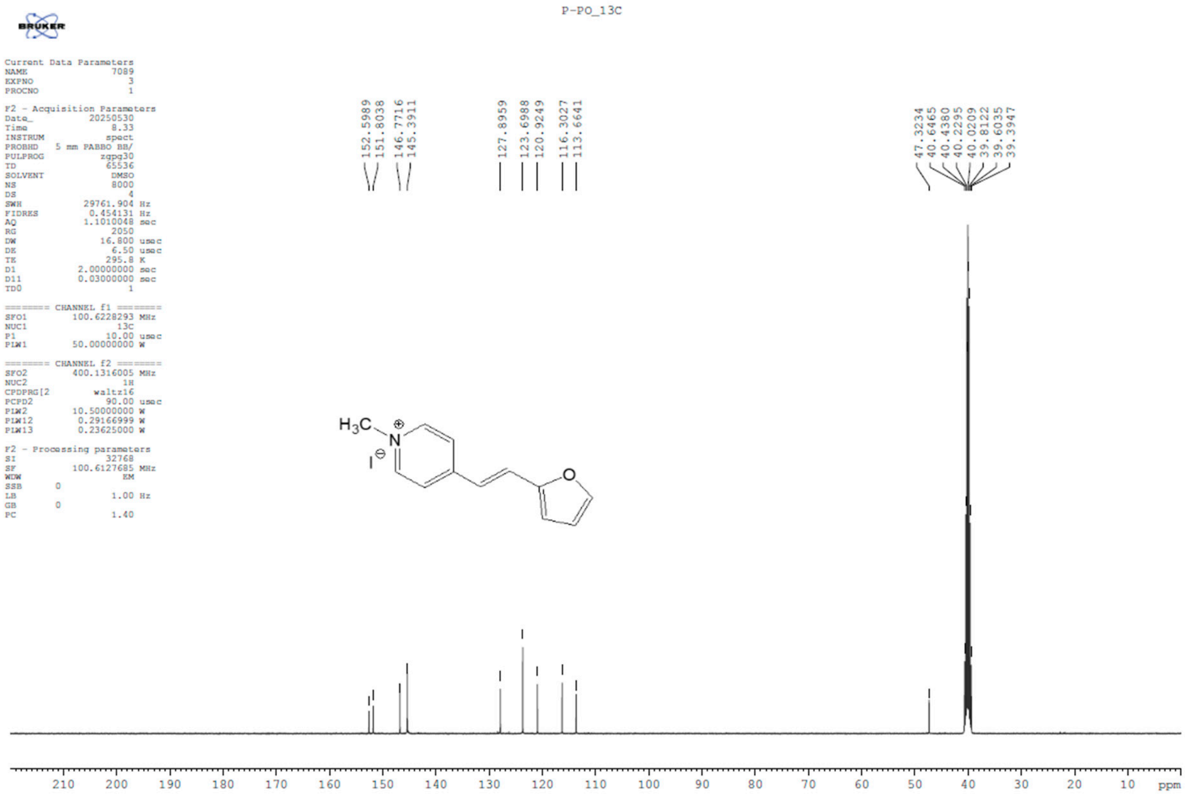

^{13}C NMR spectrum of the **2-QO**

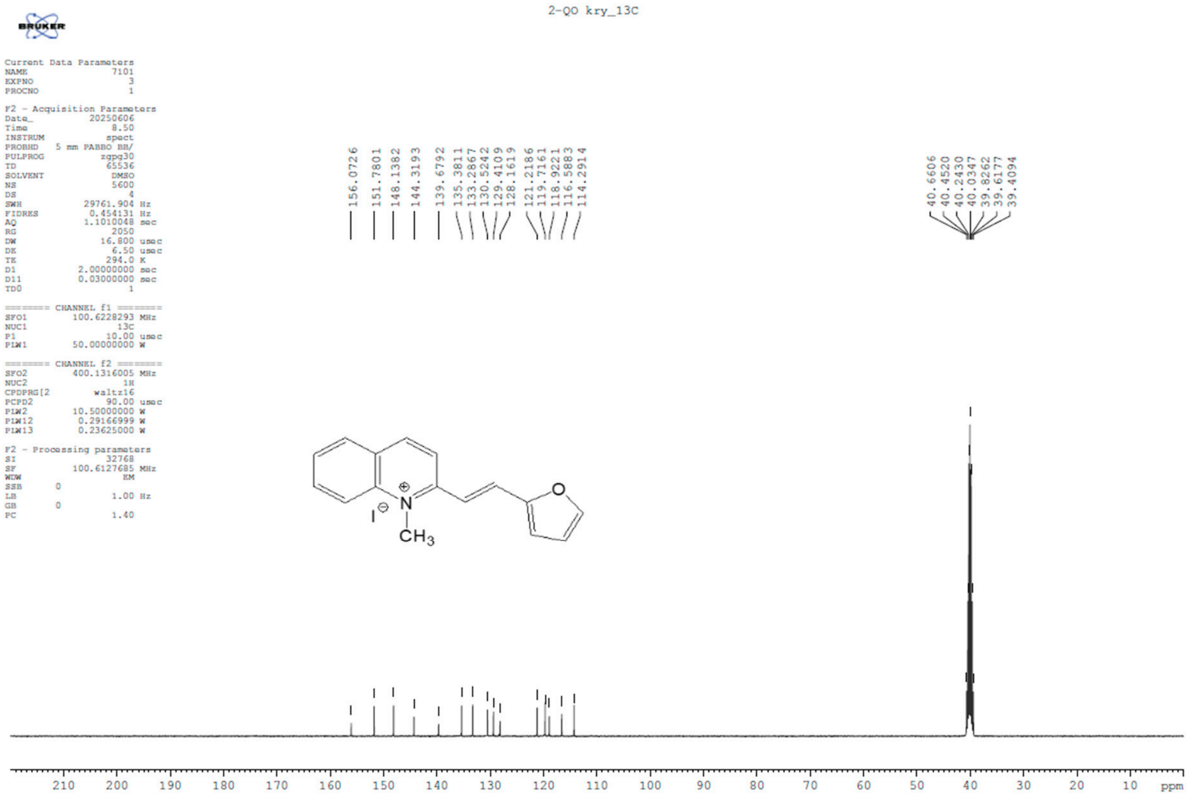

^{13}C NMR spectrum of the **4-QO**

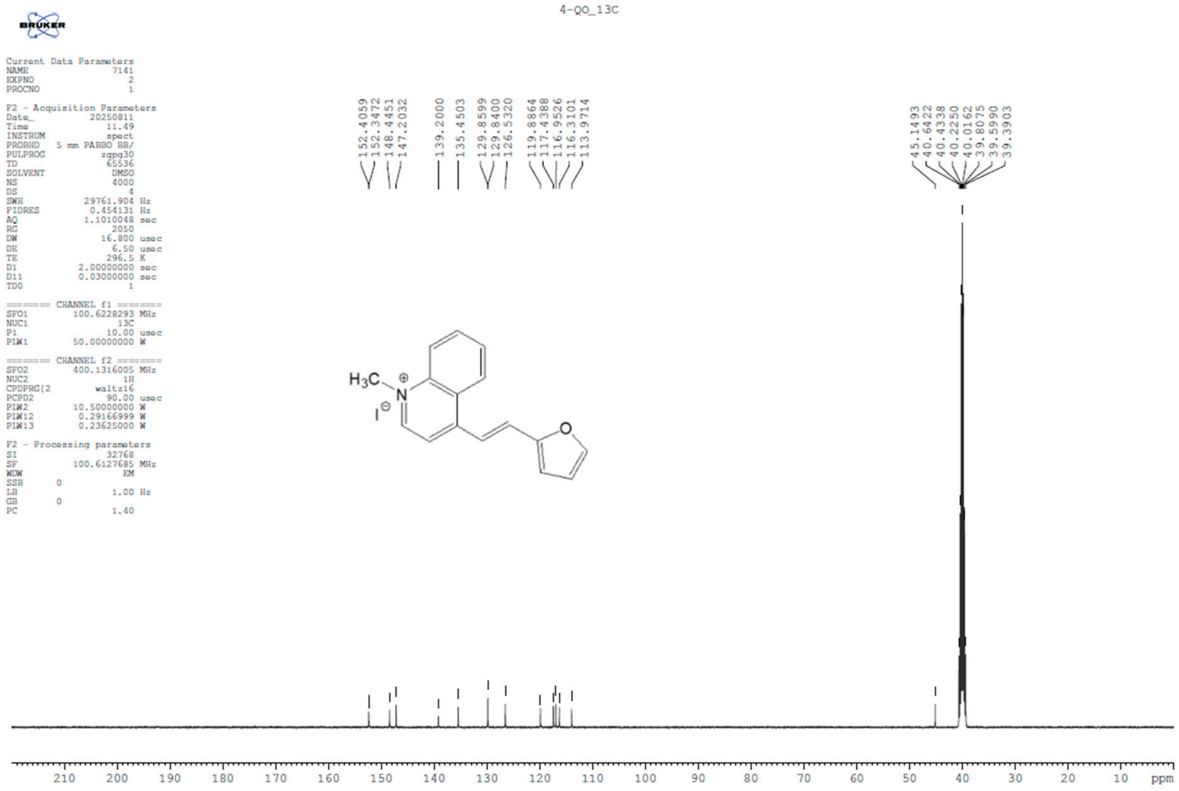

IR spectrum of the **2-PS**

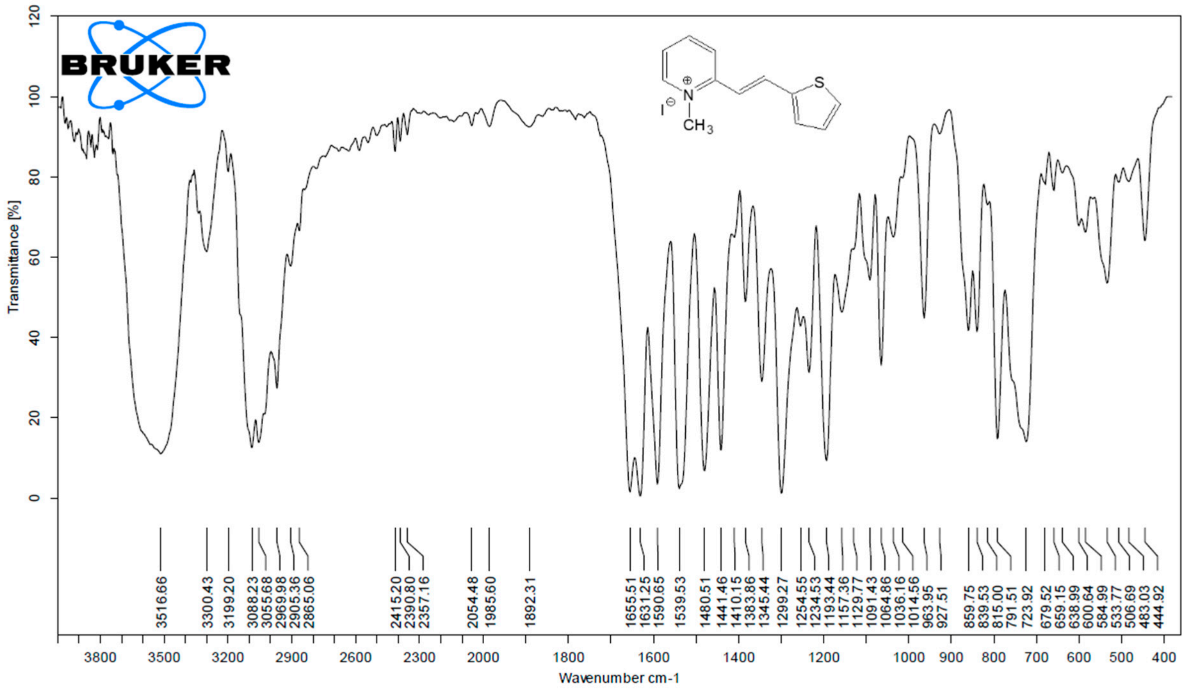

IR spectrum of the **4-PS**

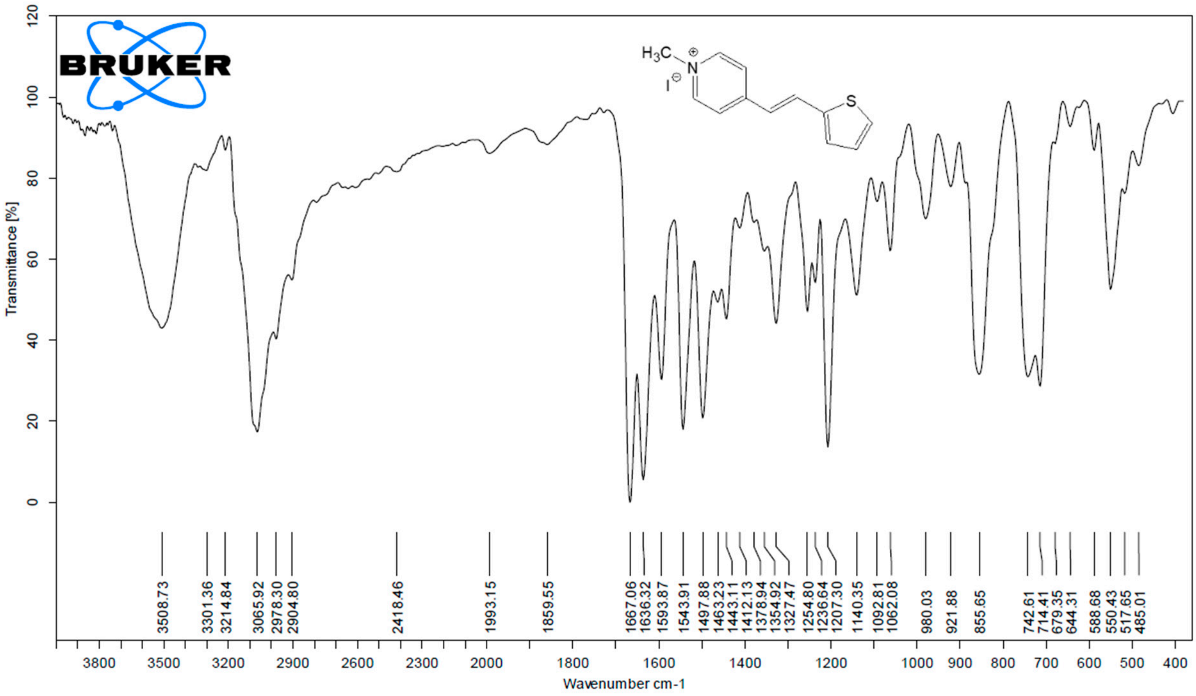

IR spectrum of the **2-QS**

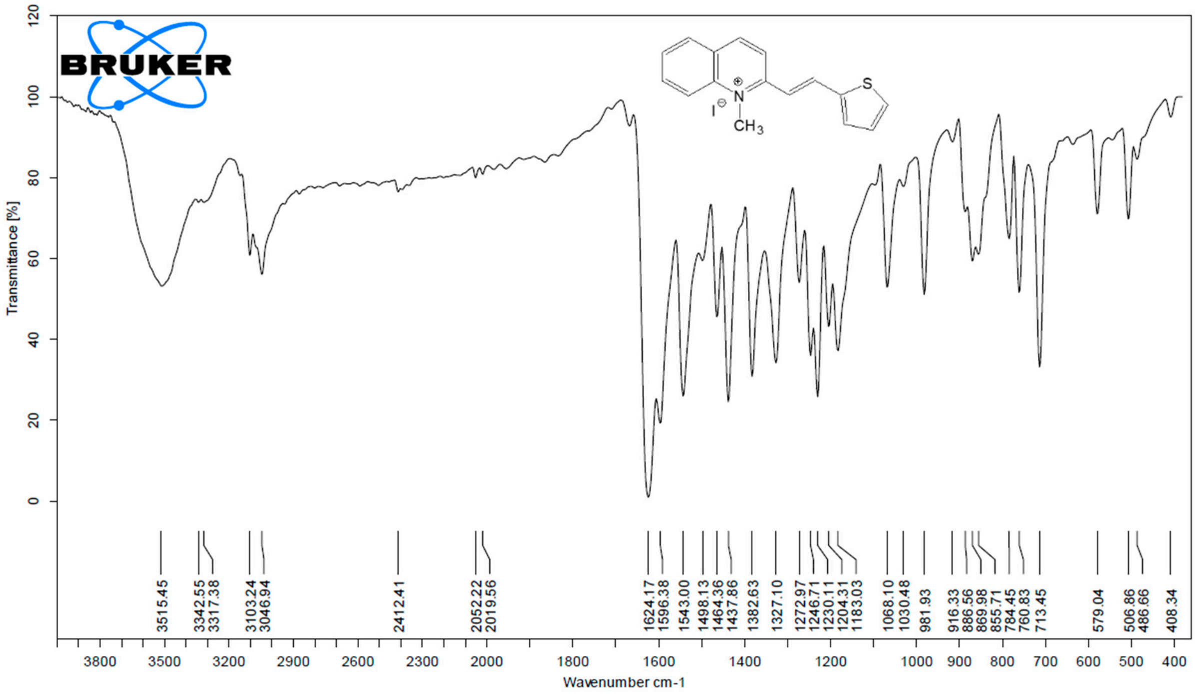

IR spectrum of the **4-QS**

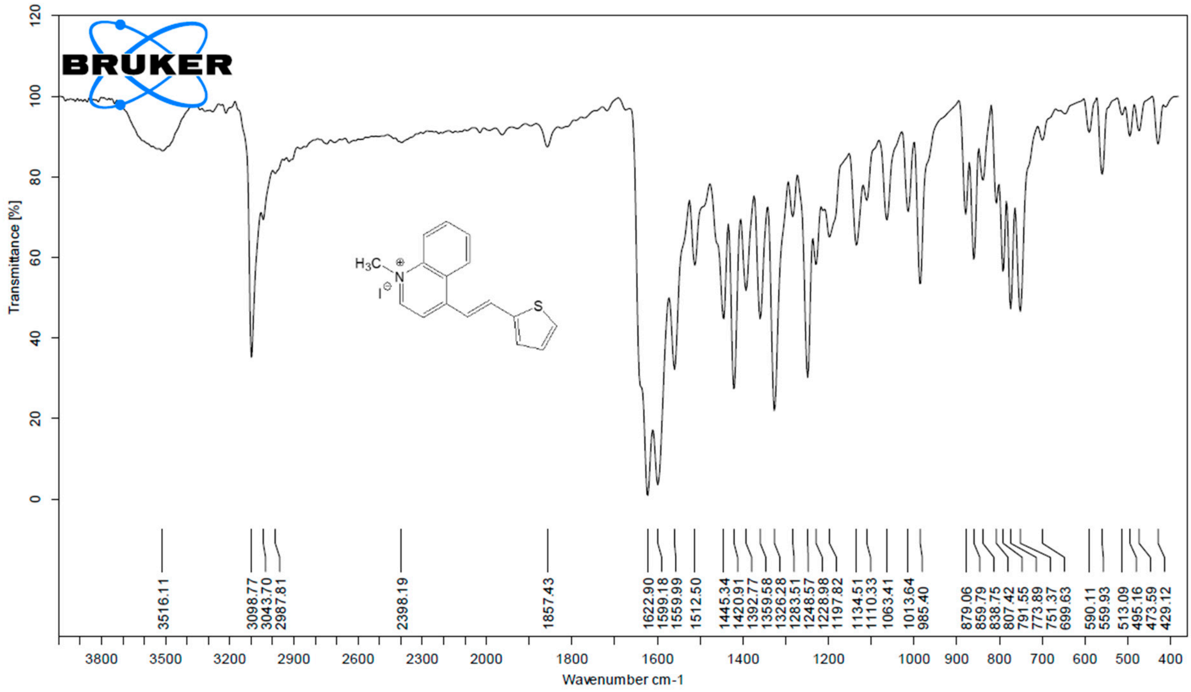

IR spectrum of the **2-PO**

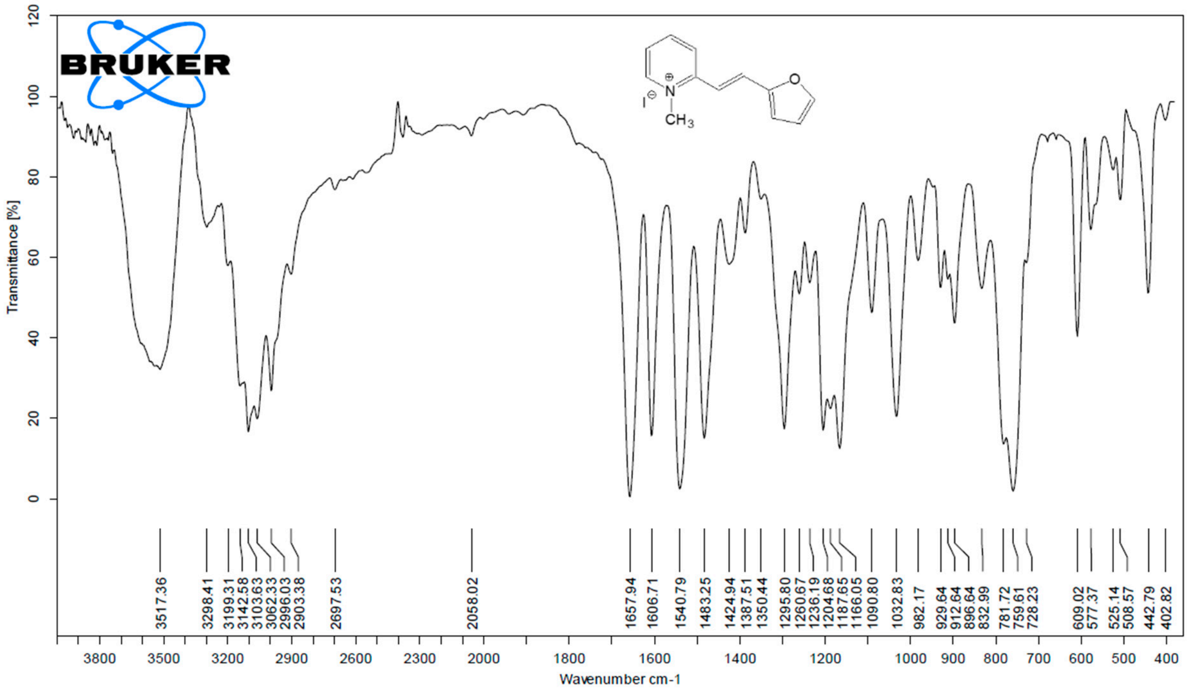

IR spectrum of the **4-PO**

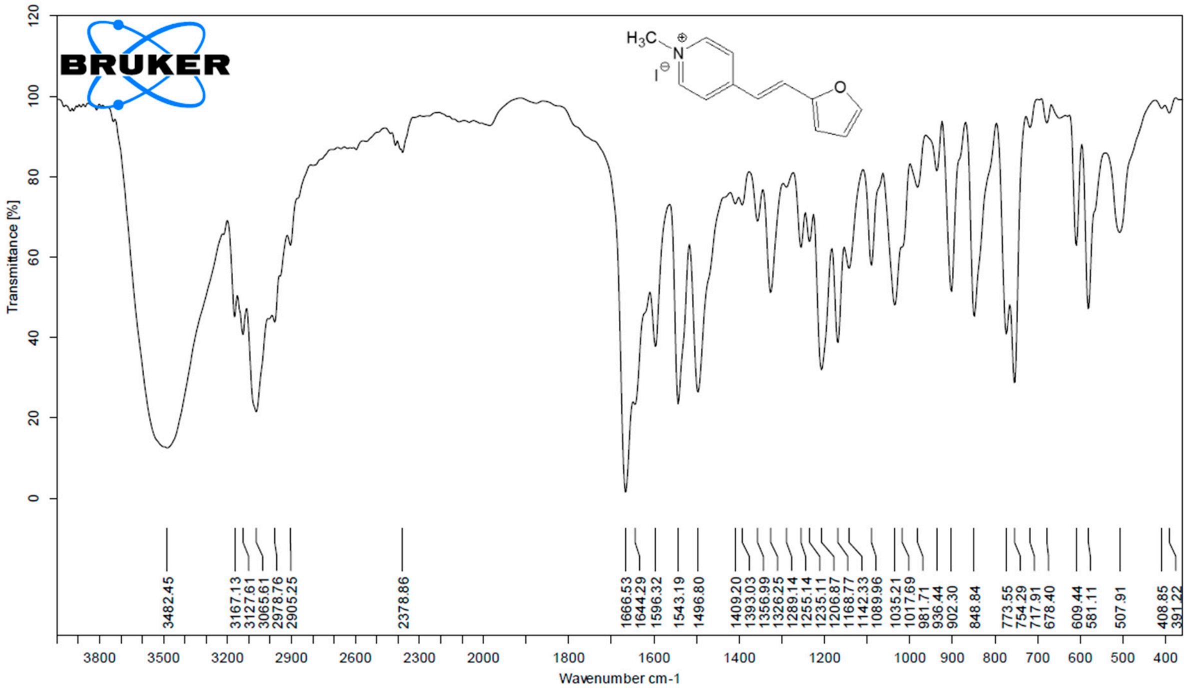

IR spectrum of the **2-QO**

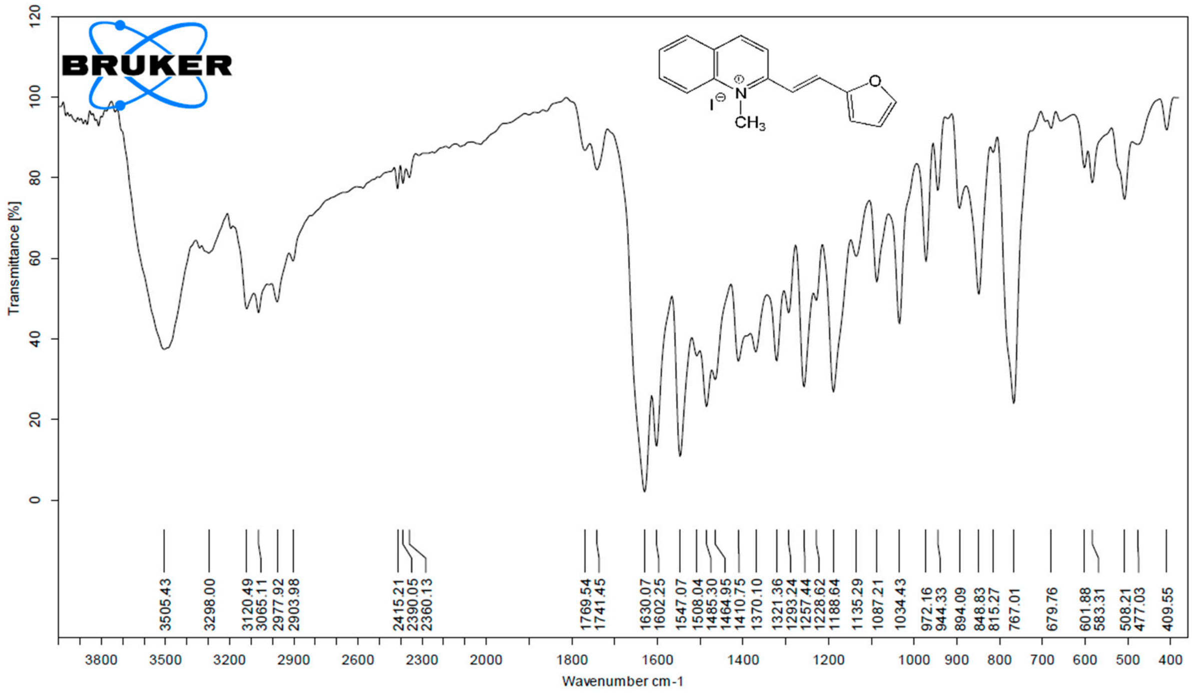

IR spectrum of the **4-QO**

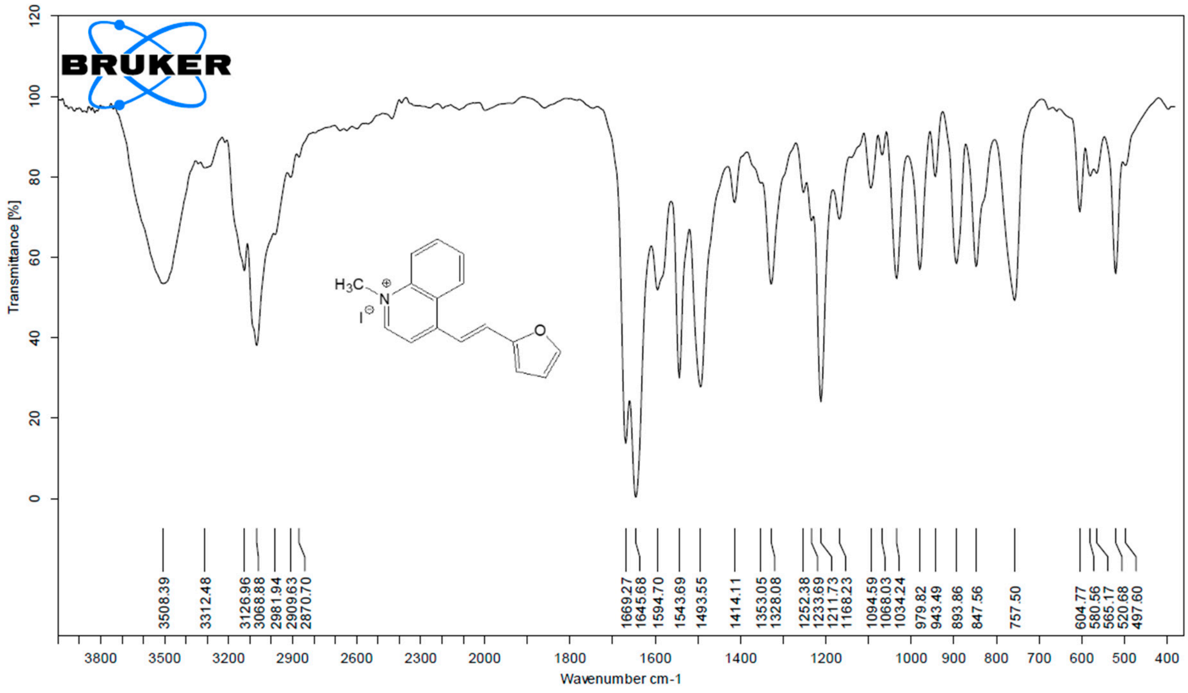

IR spectrum of the **2-PN**

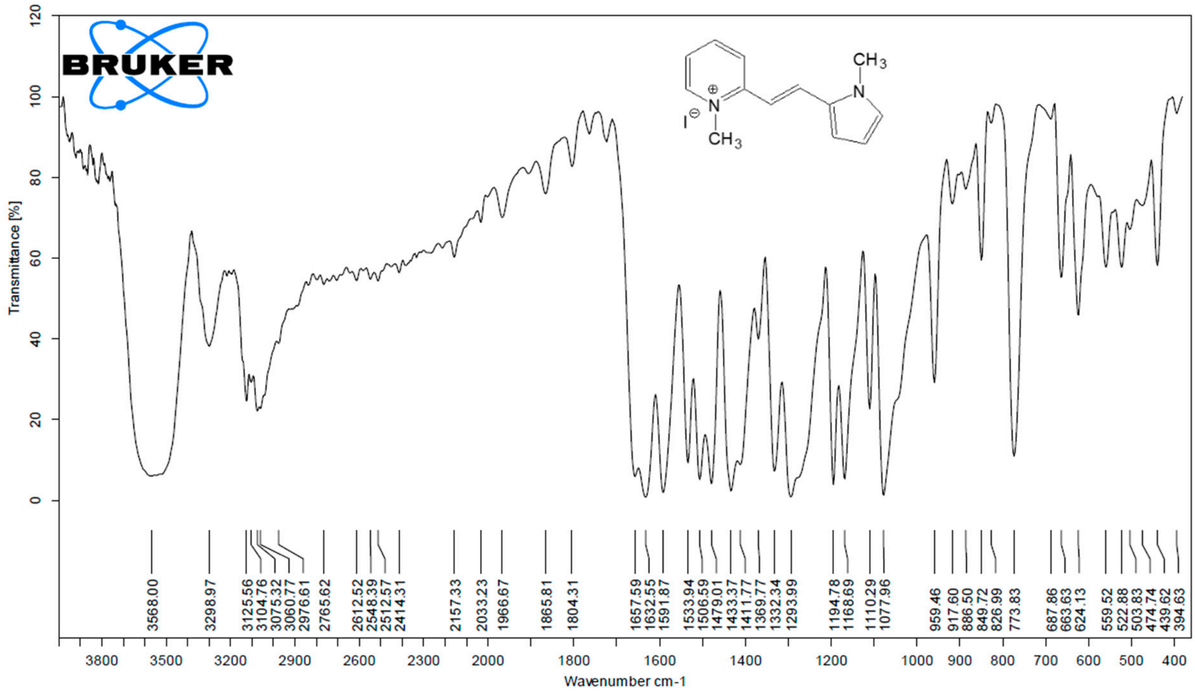

IR spectrum of the **4-PN**

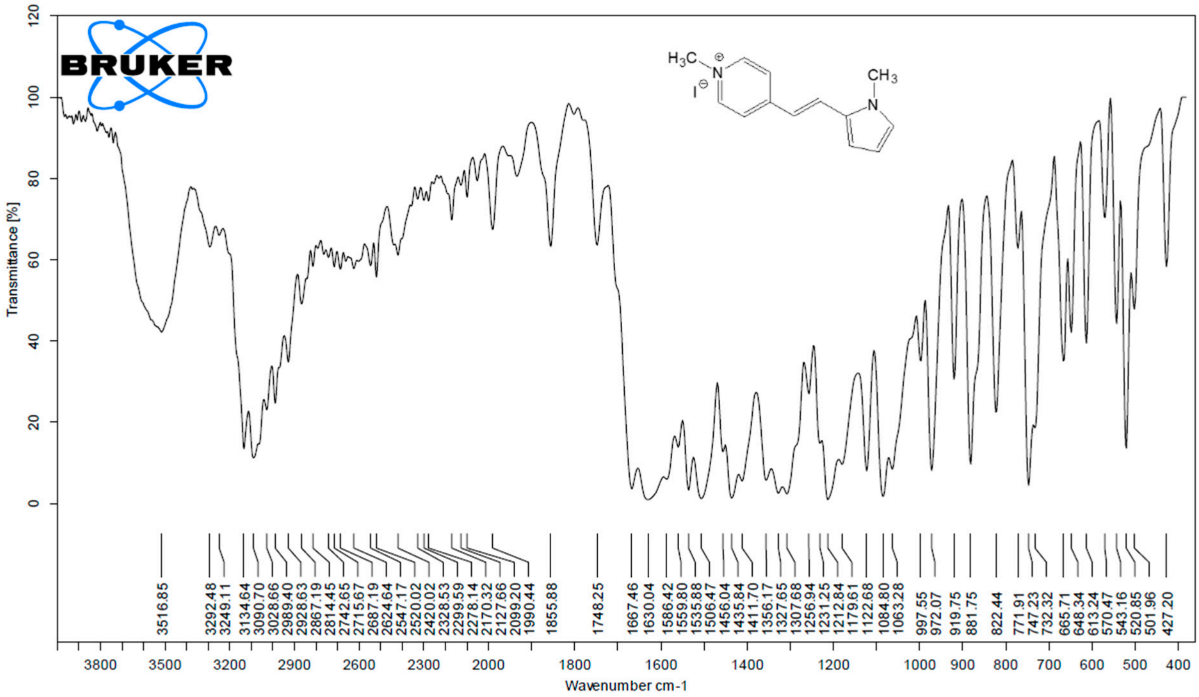

IR spectrum of the **2-QN**

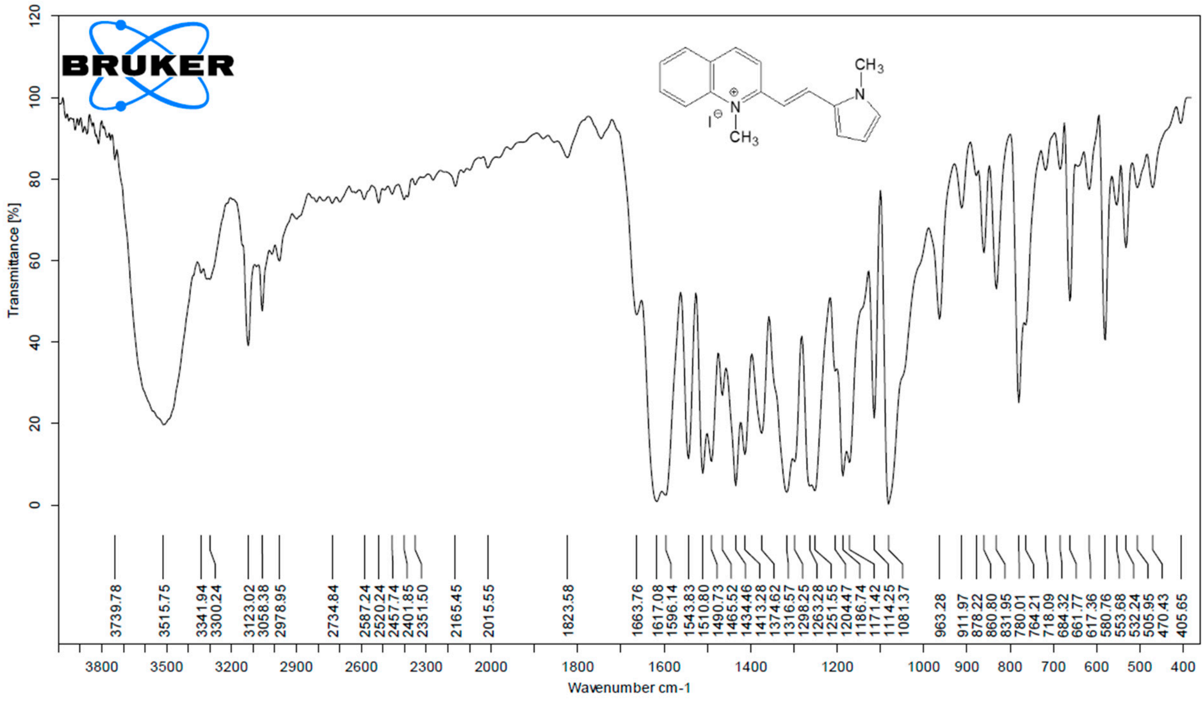

IR spectrum of the **4-QN**

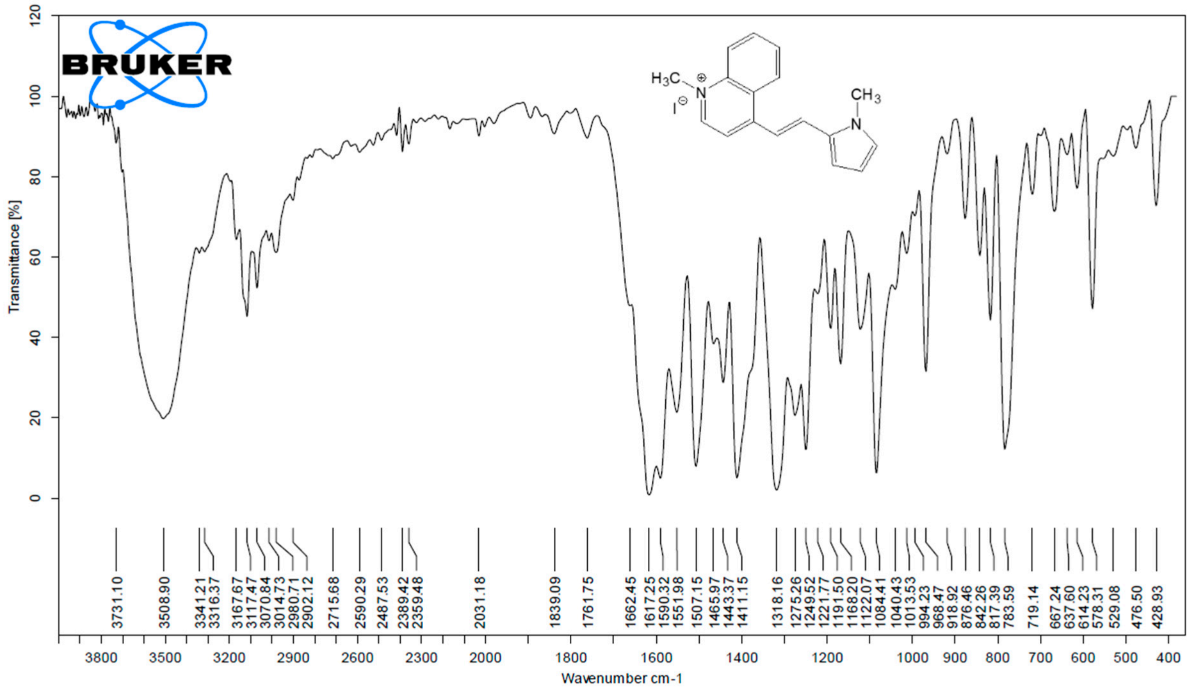

HPLC analyses were done by HPLC systems equipped with UV-Vis detector (detection wavelength was set at 366 nm, 420 nm or 490 nm, respectively), Binary HPLC Pump and a Symmetry C18 column (3.5 µm, 4.6×75 mm). Separation was conducted under isocratic conditions with 1.0 mL/min flow rate at r.t., 10 µL injection volume and HPLC grade methanol with 0.05% TFA as a mobile phase.

HPLC chromatogram of the **2-PS**

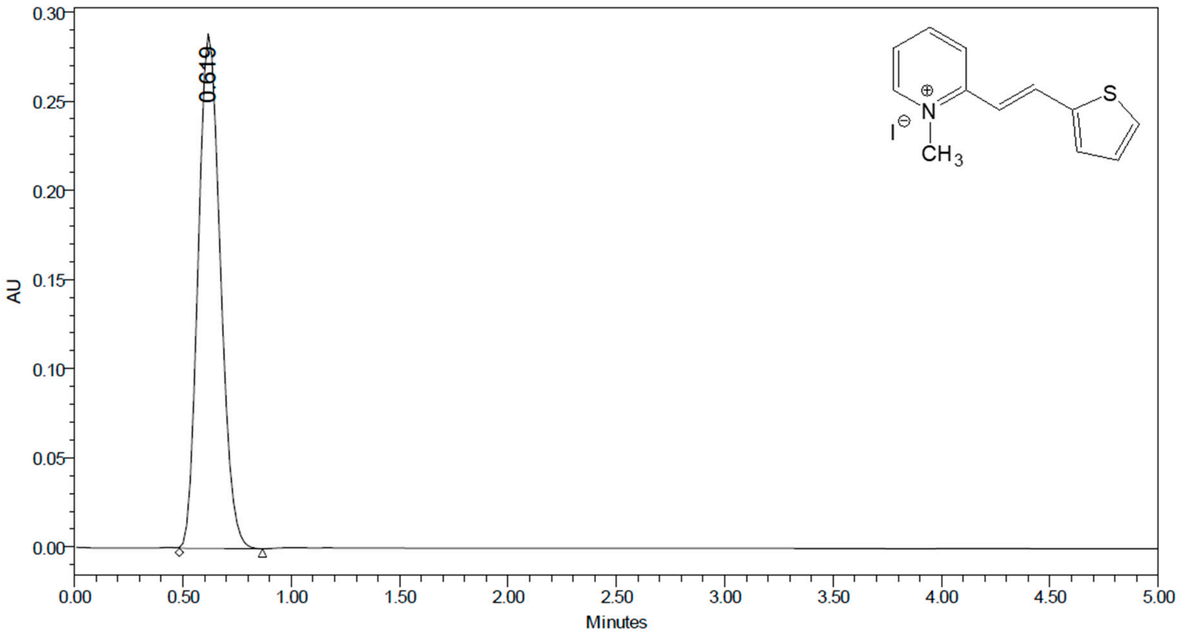

	RT (min)	Peak Type	Area (µV*sec)	% Area	Height (µV)	% Height	Integration Type	Points Across Peak	Start Time (min)	End Time (min)
1	0.619	Unknown	2052691	100.00	288122	100.00	VB	23	0.483	0.867

HPLC chromatogram of the **4-PS**

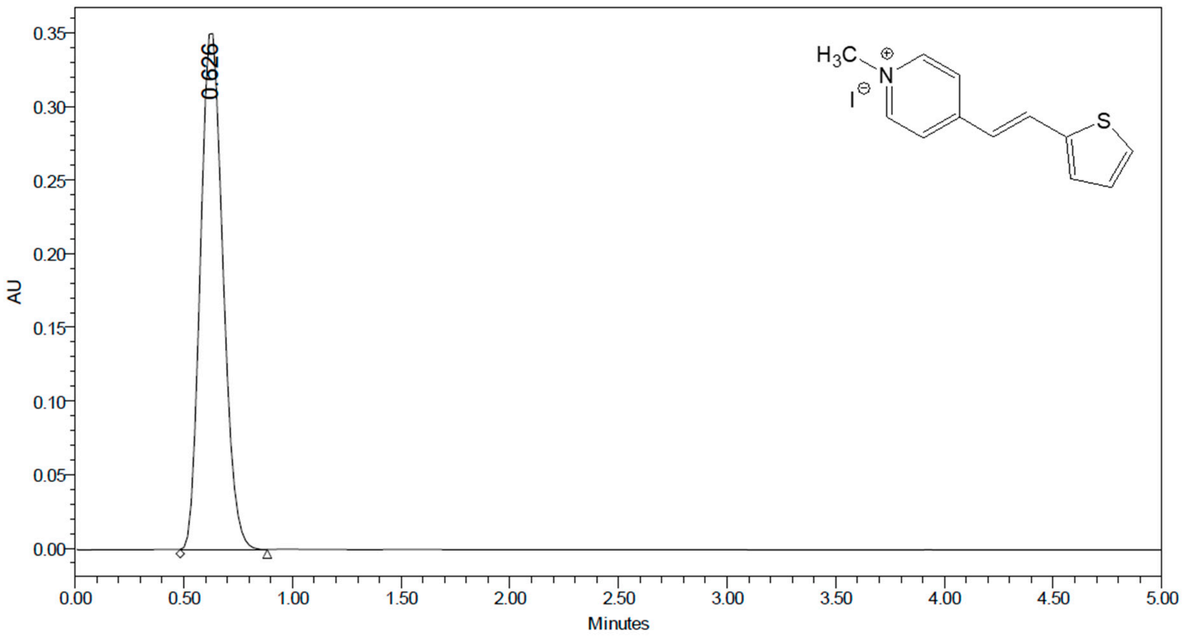

	RT (min)	Peak Type	Area (µV*sec)	% Area	Height (µV)	% Height	Integration Type	Points Across Peak	Start Time (min)	End Time (min)
1	0.626	Unknown	2529666	100.00	352711	100.00	VB	24	0.483	0.883

HPLC chromatogram of the **2-QS**

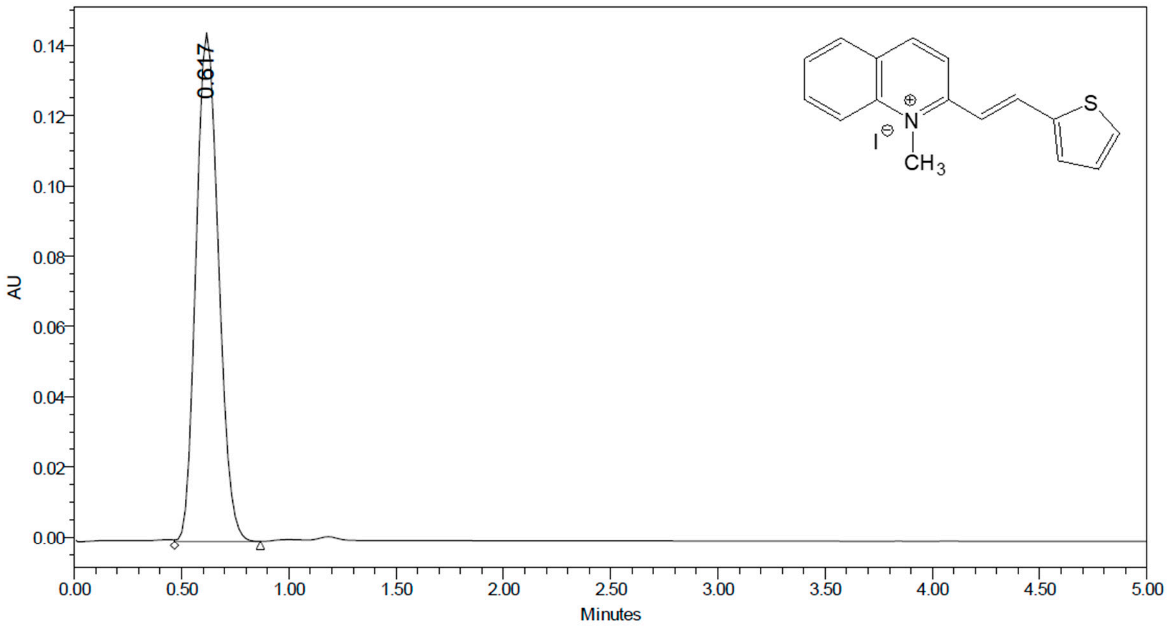

	RT (min)	Peak Type	Area (µV*sec)	% Area	Height (µV)	% Height	Integration Type	Points Across Peak	Start Time (min)	End Time (min)
1	0.617	Unknown	1056069	100.00	144380	100.00	VB	24	0.467	0.867

HPLC chromatogram of the **4-QS**

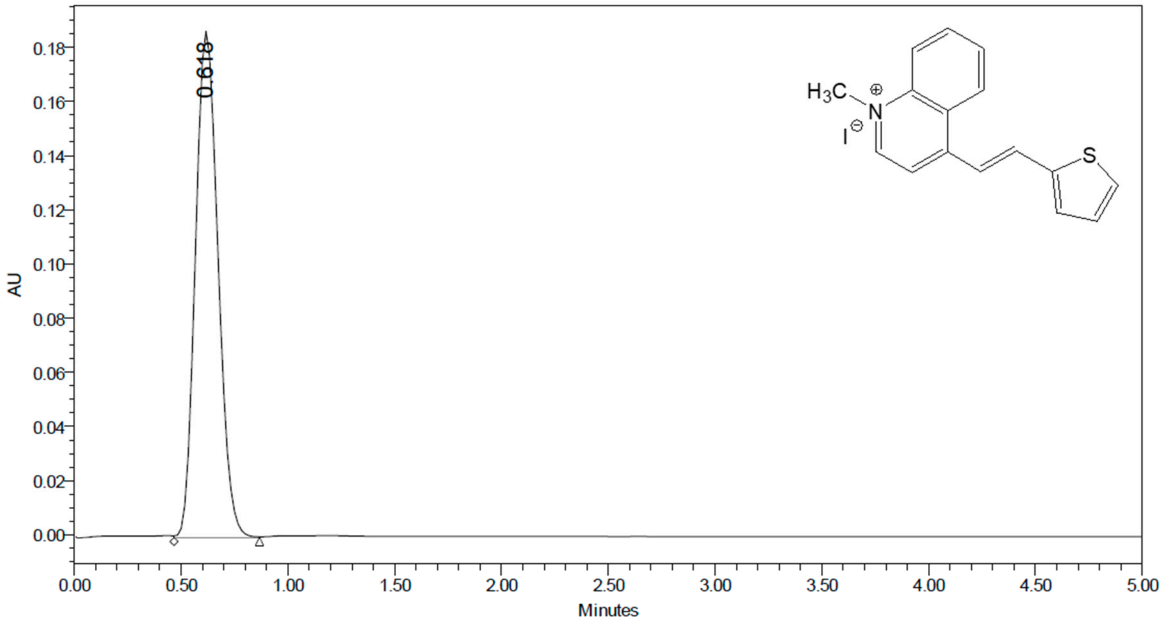

	RT (min)	Peak Type	Area (µV*sec)	% Area	Height (µV)	% Height	Integration Type	Points Across Peak	Start Time (min)	End Time (min)
1	0.618	Unknown	1379088	100.00	186624	100.00	VB	24	0.467	0.867

HPLC chromatogram of the **2-PO**

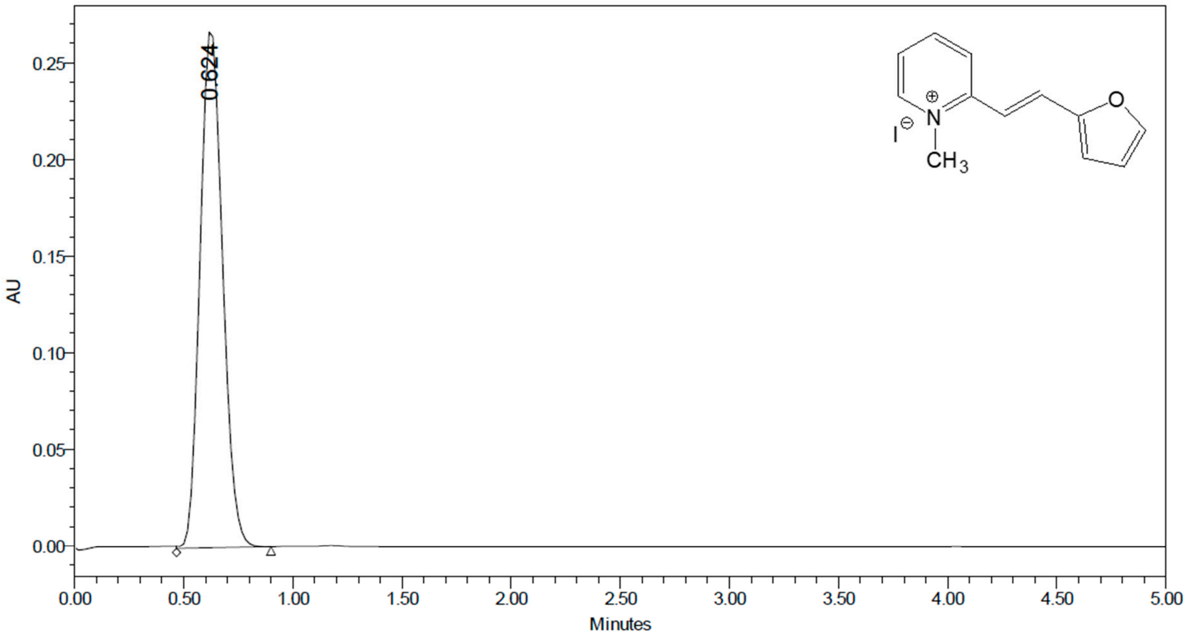

	RT (min)	Peak Type	Area (µV*sec)	% Area	Height (µV)	% Height	Integration Type	Points Across Peak	Start Time (min)	End Time (min)
1	0.624	Unknown	1925536	100.00	268933	100.00	VB	26	0.467	0.900

HPLC chromatogram of the **4-PO**

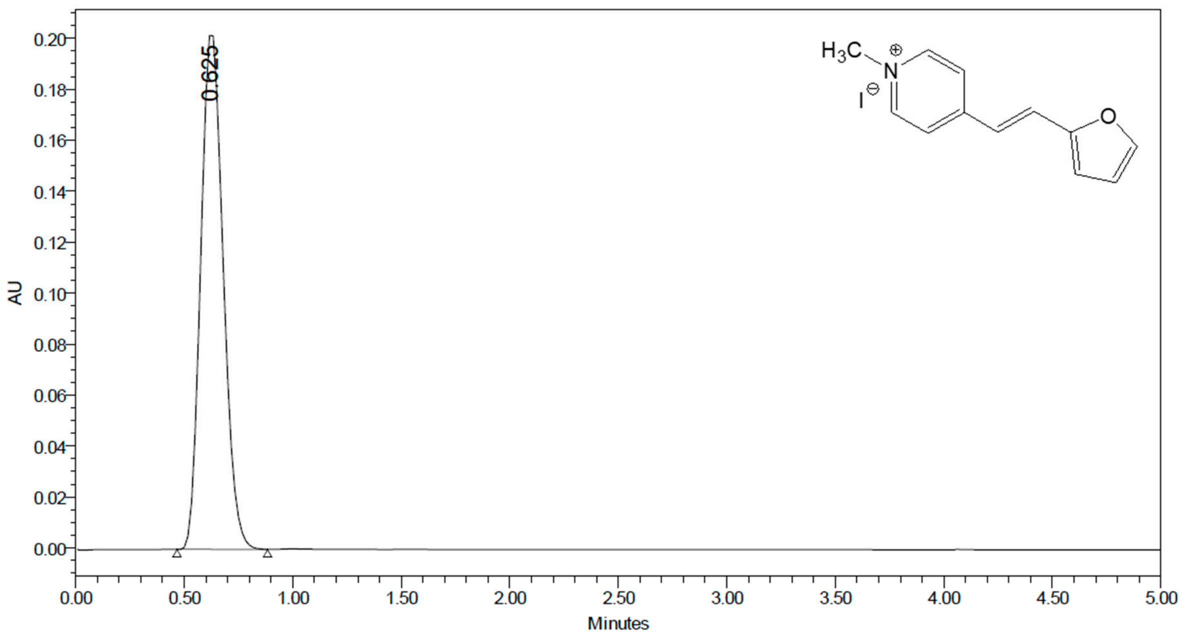

	RT (min)	Peak Type	Area (µV*sec)	% Area	Height (µV)	% Height	Integration Type	Points Across Peak	Start Time (min)	End Time (min)
1	0.625	Unknown	1453919	100.00	203794	100.00	BB	25	0.467	0.883

HPLC chromatogram of the **2-QO**

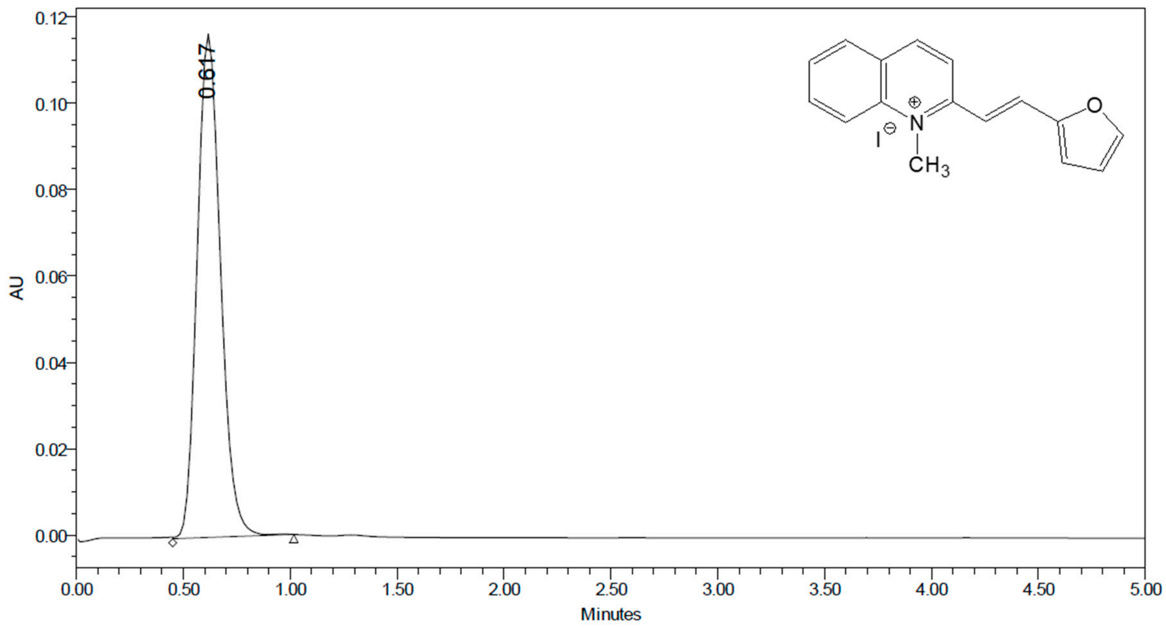

	RT (min)	Peak Type	Area (µV*sec)	% Area	Height (µV)	% Height	Integration Type	Points Across Peak	Start Time (min)	End Time (min)
1	0.617	Unknown	885134	100.00	116285	100.00	VB	34	0.450	1.017

HPLC chromatogram of the **4-QO**

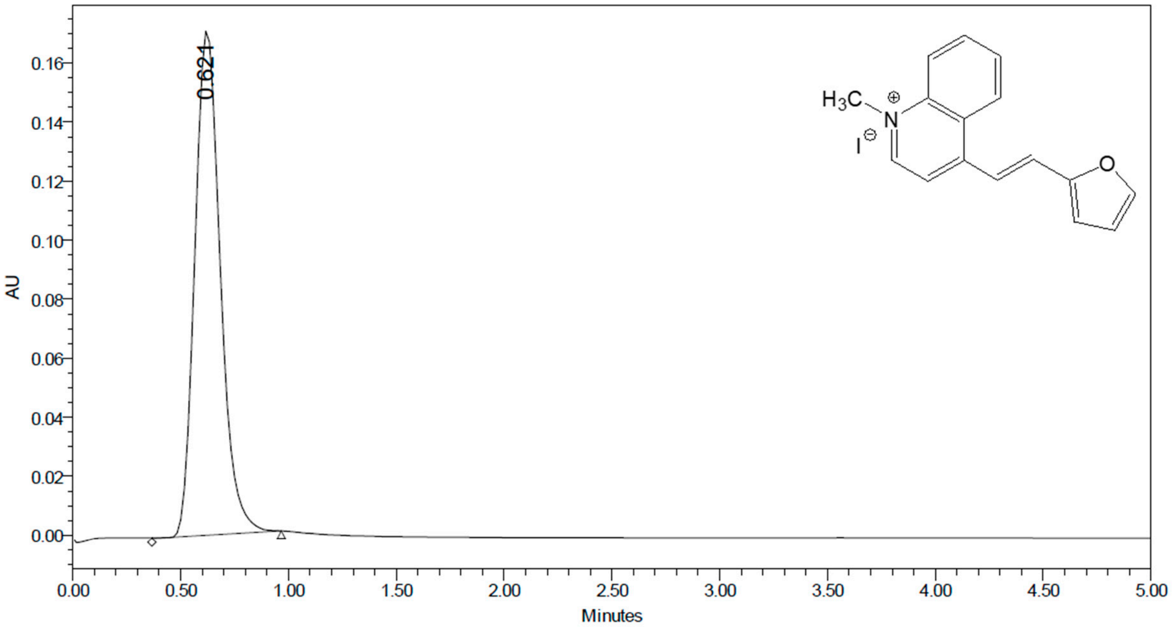

	RT (min)	Peak Type	Area (µV*sec)	% Area	Height (µV)	% Height	Integration Type	Points Across Peak	Start Time (min)	End Time (min)
1	0.621	Unknown	1405332	100.00	170964	100.00	Vb	36	0.367	0.967

HPLC chromatogram of the **2-PN**

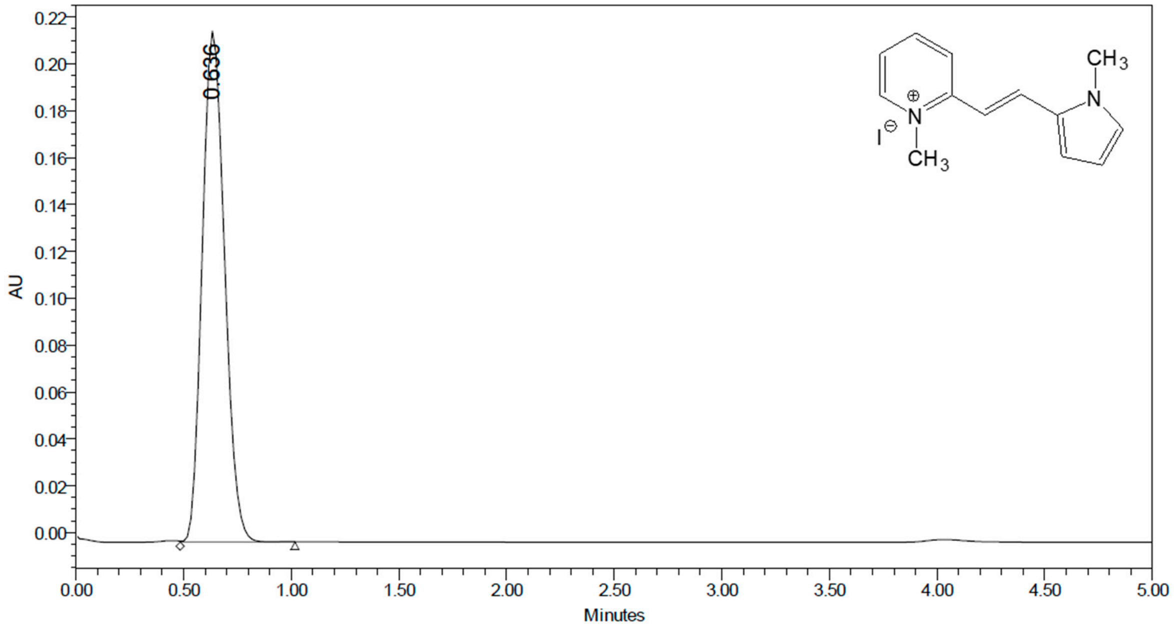

	RT (min)	Peak Type	Area (µV*sec)	% Area	Height (µV)	% Height	Integration Type	Points Across Peak	Start Time (min)	End Time (min)
1	0.636	Unknown	1569192	100.00	217634	100.00	VB	32	0.483	1.017

HPLC chromatogram of the **4-PN**

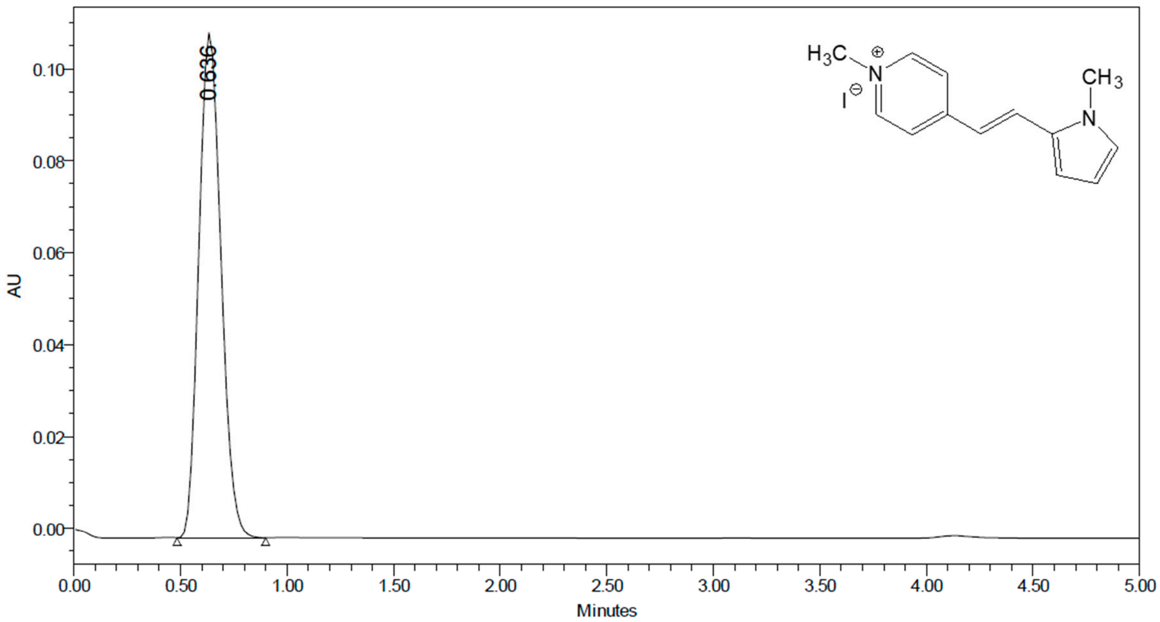

	RT (min)	Peak Type	Area (μV*sec)	% Area	Height (μV)	% Height	Integration Type	Points Across Peak	Start Time (min)	End Time (min)
1	0.636	Unknown	795011	100.00	109799	100.00	BB	25	0.483	0.900

HPLC chromatogram of the **2-QN**

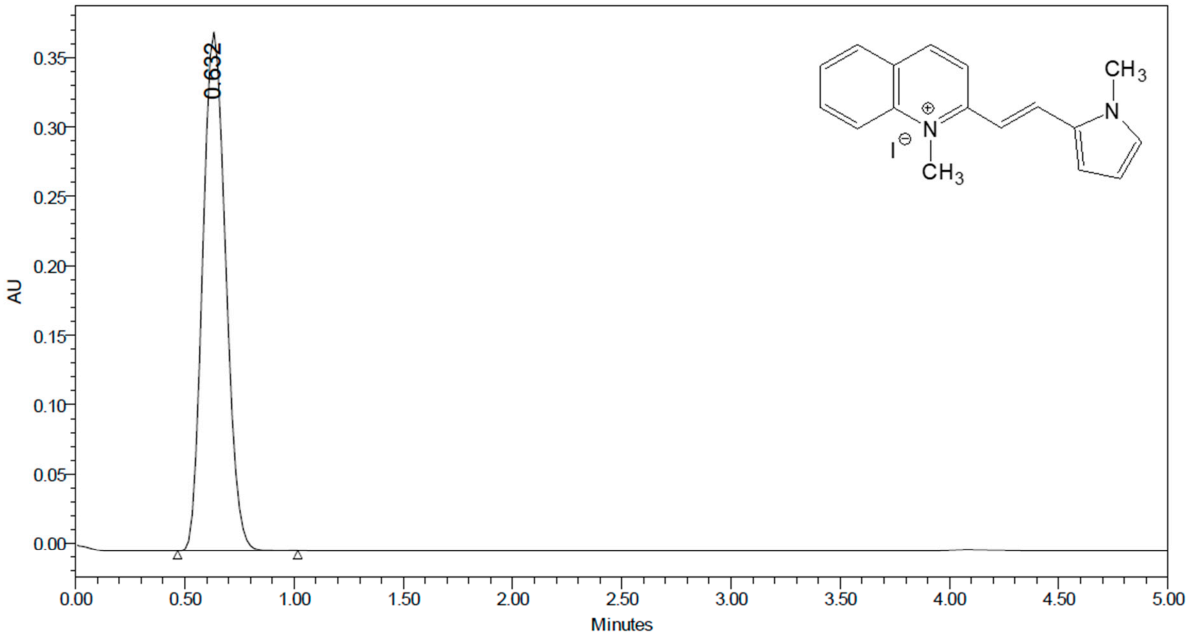

	RT (min)	Peak Type	Area (μV*sec)	% Area	Height (μV)	% Height	Integration Type	Points Across Peak	Start Time (min)	End Time (min)
1	0.632	Unknown	2727544	100.00	372911	100.00	BB	33	0.467	1.017

HPLC chromatogram of the **4-QN**

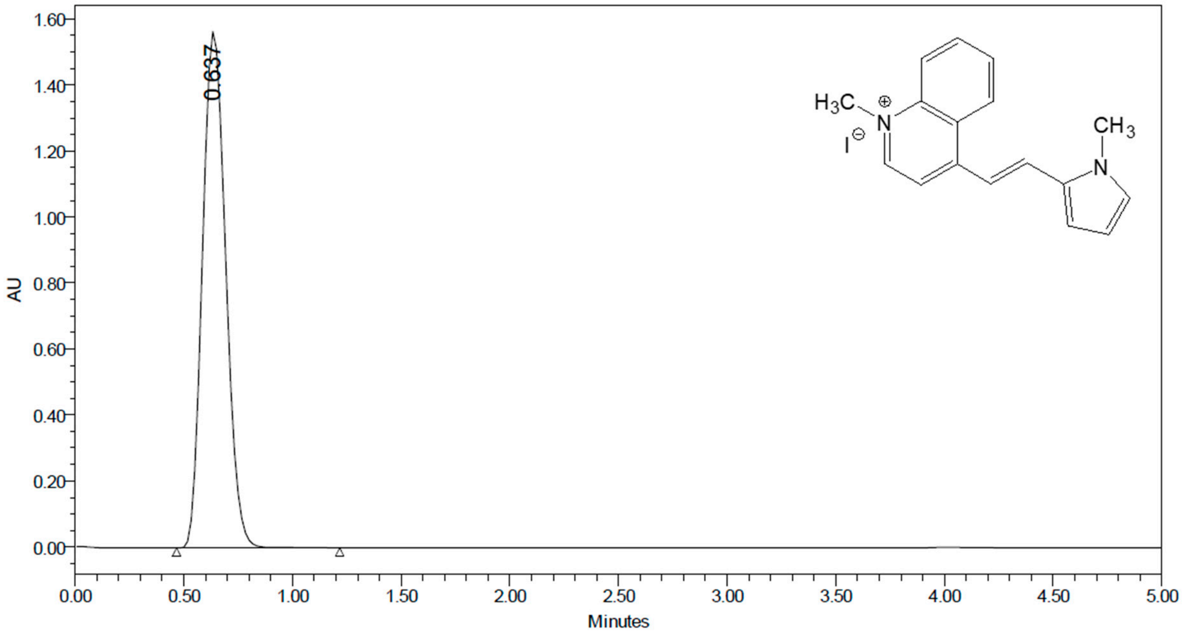

	RT (min)	Peak Type	Area (µV*sec)	% Area	Height (µV)	% Height	Integration Type	Points Across Peak	Start Time (min)	End Time (min)
1	0.637	Unknown	11599800	100.00	1564421	100.00	BB	45	0.467	1.217

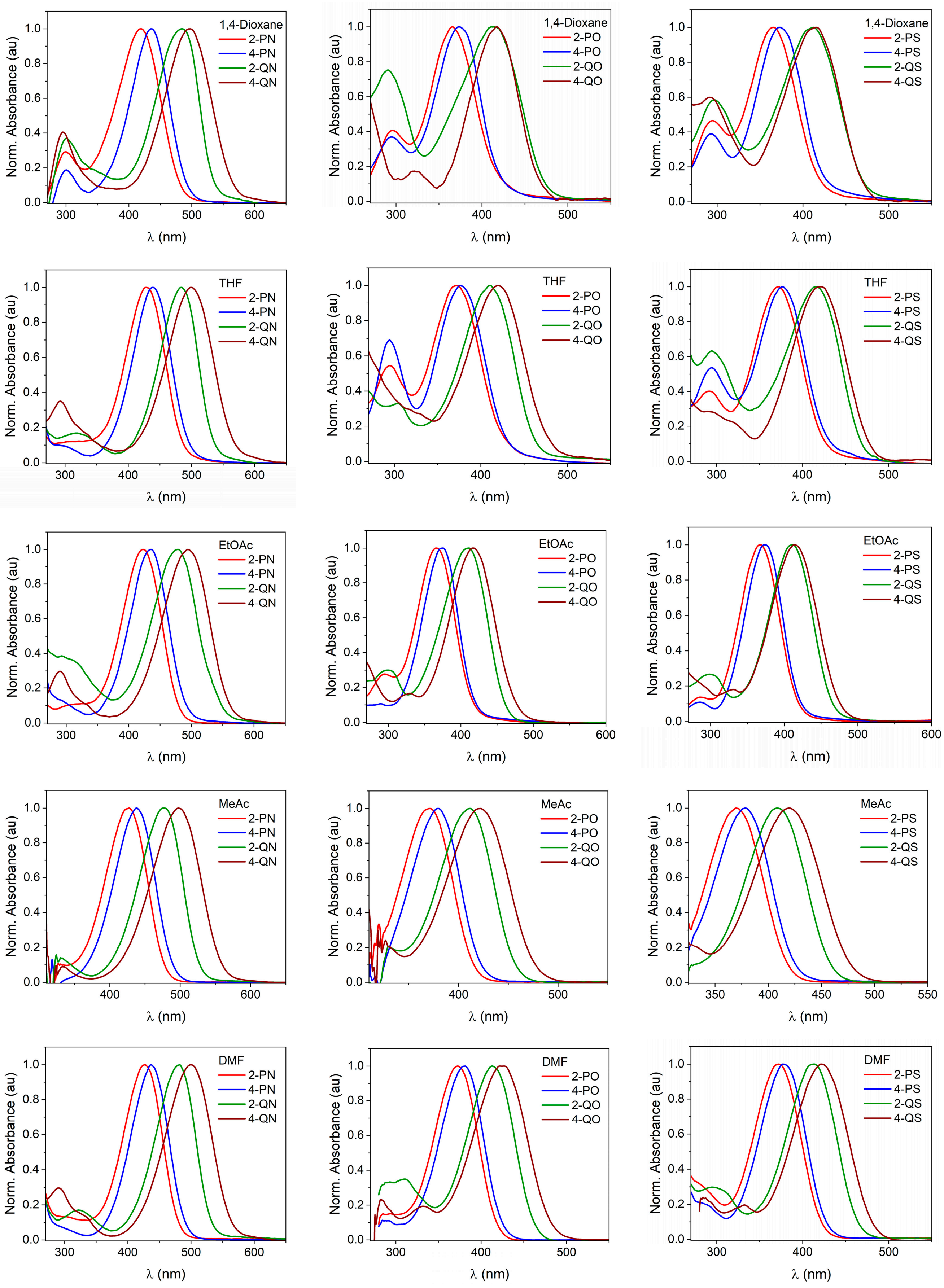

Figure S1. Electronic absorption spectra of the tested dyes in solvents of different polarity.

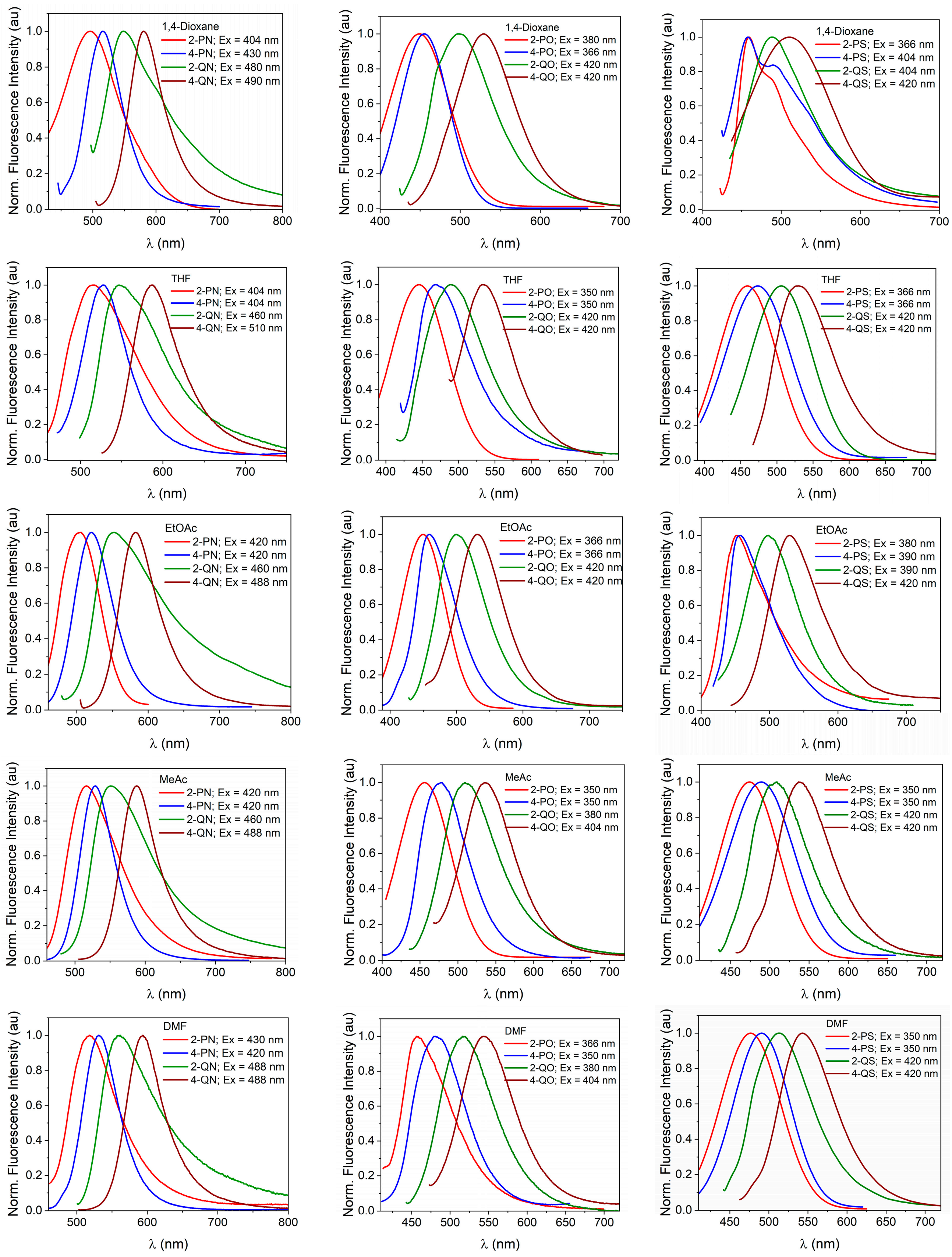

Figure S2. Fluorescence spectra of the tested dyes in solvents of different polarity.

Table S1. Estimated from Eq. (1), coefficients (y_0, a_{SP}, b_{SdP}, c_{SA}, and d_{SB}), their standard errors and correlation coefficients (R^2) for the multiple linear regression analysis of $\Delta\nu^{SS}$ of the tested dyes in 6 solvents as a function of the Catalán four-parameter solvent scale

No	y_0	a_{SP}	b_{SdP}	c_{SA}	d_{SB}	R^2
2-PN	3592±18	-(70±27)	395±6	623±67	276±16	1.000
4-PN	3369±94	336±144	555±31	853±357	-(242±85)	0.997
2-QN	2865±481	-(619±736)	581±159	3335±1824	-(412±435)	0.950
4-QN	2599±418	443±639	323±138	407±1585	-(210±378)	0.849
2-PO	4436±200	527±305	929±66	4639±756	-(1316±180)	0.997
4-PO	3515±180	1608±275	1358±59	2394±682	-(975±163)	0.998
2-QO	2610±774	1929±1184	1255±256	738±2934	-(764±700)	0.963
4-QO	5100±407	-(158±622)	317±135	2872±1543	-(152±368)	0.932
2-PS	5128±1403	1795±2145	1054±464	3376±5318	-(2944±1269)	0.817
4-PS	1061±511	5717±782	2157±169	-(8322±1938)	-(2204±462)	0.989
2-QS	4904±224	-(2215±343)	1466±74	6971±850	-(272±203)	0.998
4-QS	5277±40	-(1387±61)	593±13	4331±152	371±36	1.000

	2-PN	4-PN
LUMO	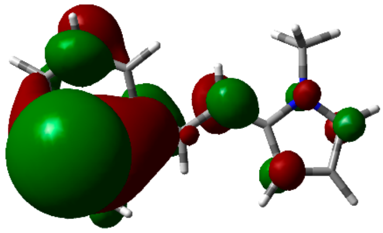	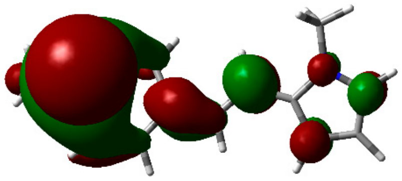
HOMO	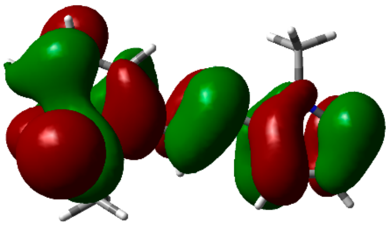	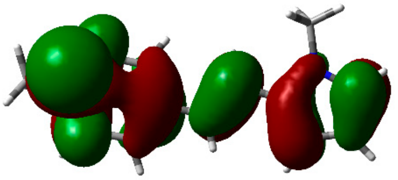
	2-PO	4-PO
LUMO	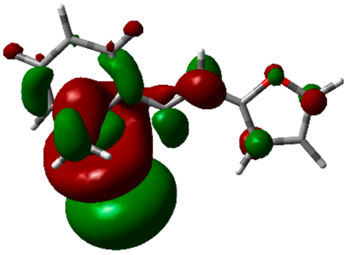	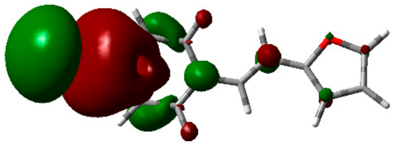
HOMO	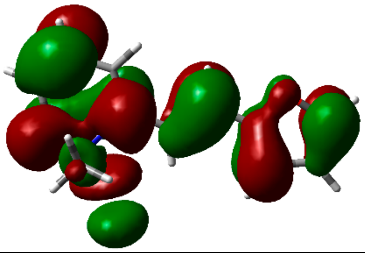	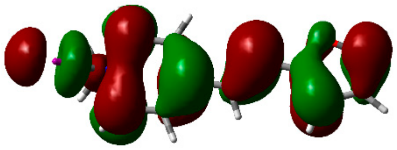
	2-PS	4-PS
LUMO	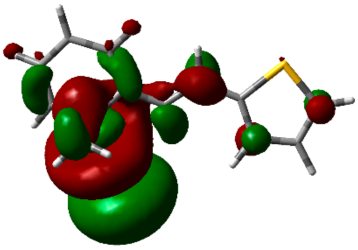	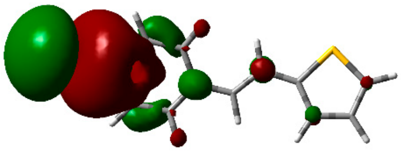
HOMO	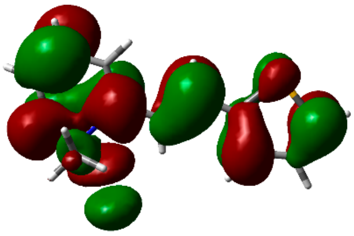	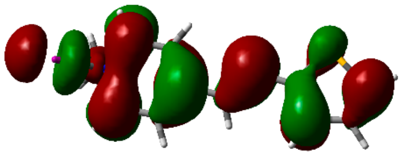

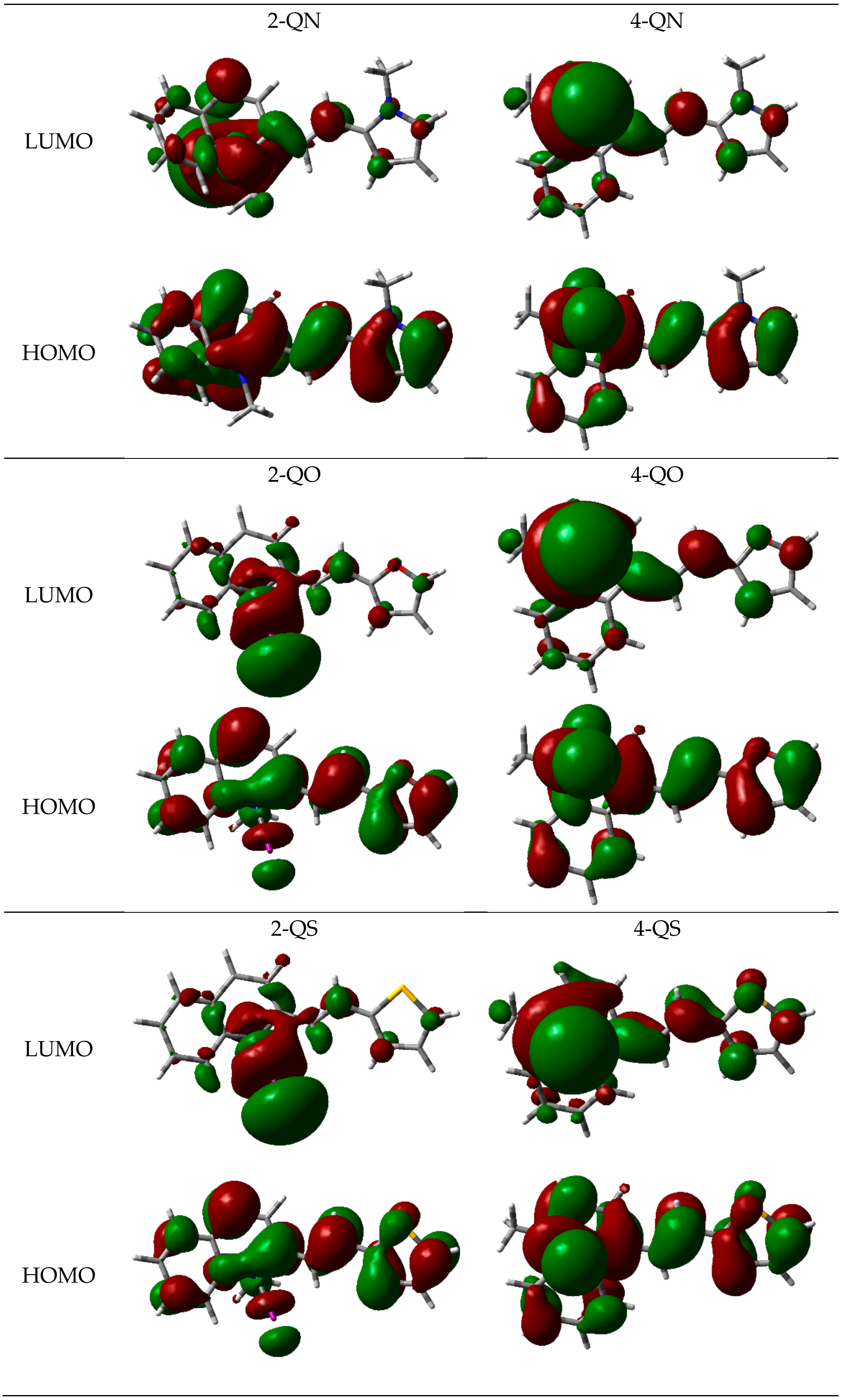

Figure S3. The HOMO/LUMO plots.

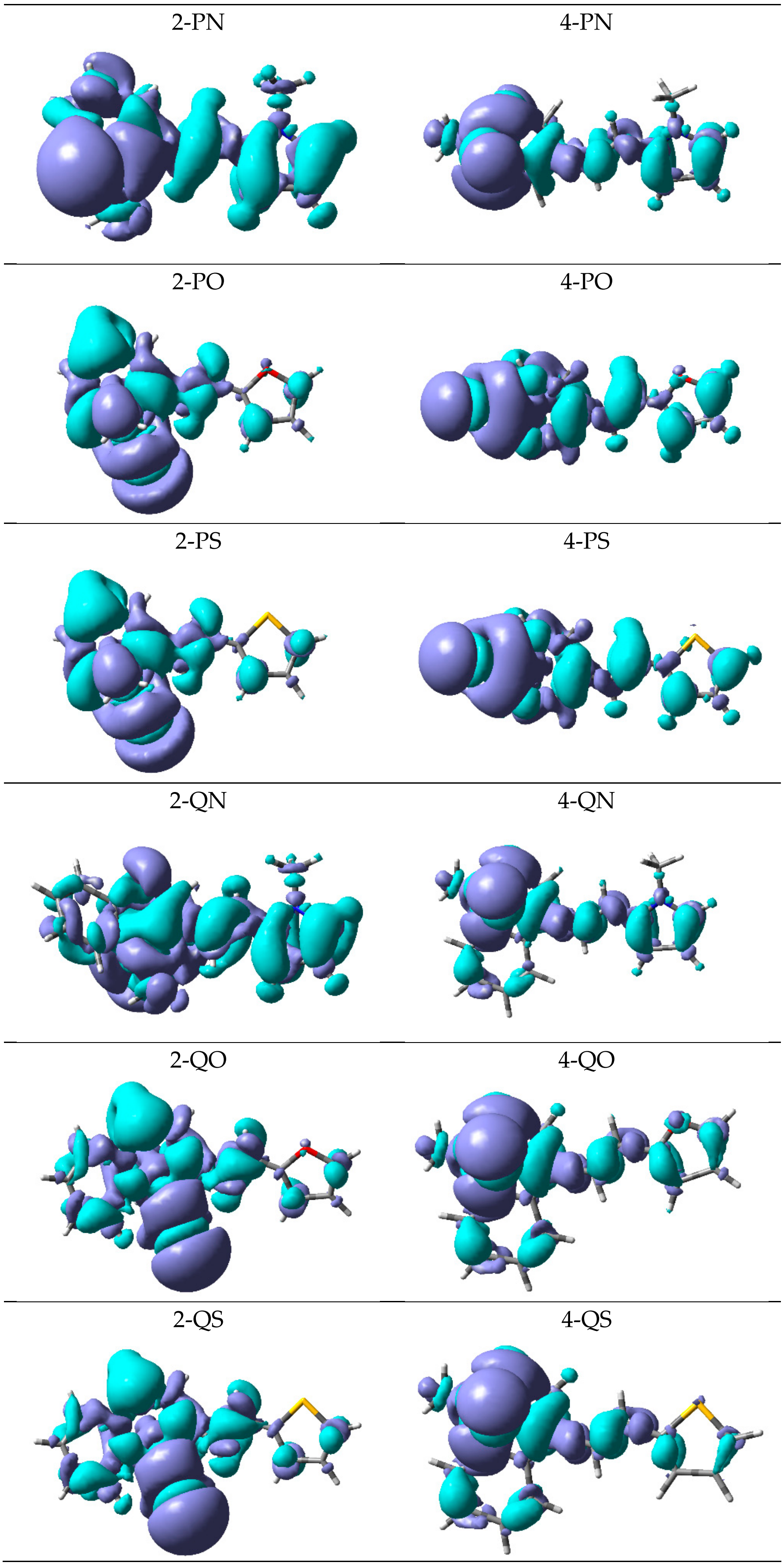

Figure S4. The density differnces plots.

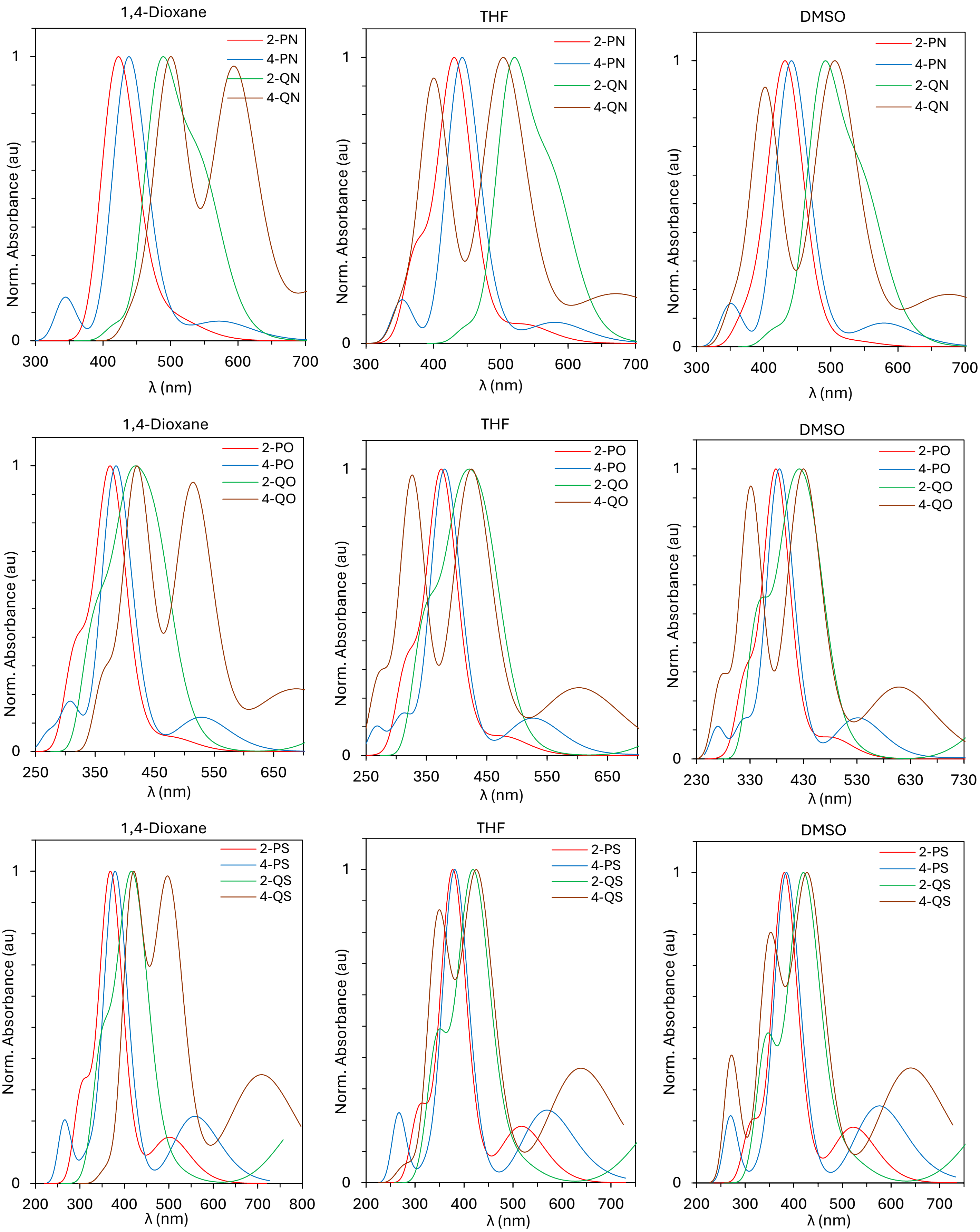

Figure S5. Graphical representation of theoretically determined absorption spectra.

Table S2. The frontier orbital energies (ΔE_{GAP} – energy gap, χ – absolute electronegativity, μ – chemical potentials, η – absolute hardness , σ – absolute softness, ω – global electrophilicity, S – global softness).

No	Solvent	E_{HOMO}	E_{LUMO}	ΔE_{GAP}	η	μ	χ	σ	*pi*	*S*	ω
2-PN	1.4-Dx	-4.5659	-1.9469	2.6191	1.3095	-3.2564	3.2564	0.7636	-3.2564	0.6548	4.0489
	THF	-4.6405	-2.0446	2.5959	1.2980	-3.3425	3.3425	0.7704	-3.3425	0.6490	4.3039
	DMSO	-4.5314	-2.0946	2.4367	1.2184	-3.3130	3.3130	0.8208	-3.3130	0.6092	4.5044
4-PN	1.4-Dx	-4.4614	-2.0454	2.4160	1.2080	-3.2534	3.2534	0.8278	-3.2534	0.6040	4.3810
	THF	-4.5314	-2.1493	2.3820	1.1910	-3.3404	3.3404	0.8396	-3.3404	0.5955	4.6843
	DMSO	-4.5700	-2.2035	2.3665	1.1833	-3.3868	3.3868	0.8451	-3.3868	0.5916	4.8469
2-PO	1.4-Dx	-4.7711	-2.1295	2.6416	1.3208	-3.4503	3.4503	0.7571	-3.4503	0.6604	4.5065
	THF	-4.8286	-2.2016	2.6270	1.3135	-3.5151	3.5151	0.7613	-3.5151	0.6567	4.7035
	DMSO	-4.5586	-2.7129	1.8456	0.9228	-3.6358	3.6358	1.0836	-3.6358	0.4614	7.1622
4-PO	1.4-Dx	-4.6421	-2.2269	2.4152	1.2076	-3.4345	3.4345	0.8281	-3.4345	0.6038	4.8840
	THF	-4.6759	-2.3137	2.3622	1.1811	-3.4948	3.4948	0.8467	-3.4948	0.5905	5.1705
	DMSO	-4.4522	-2.7233	1.7289	0.8644	-3.5877	3.5877	1.1568	-3.5877	0.4322	7.4451
2-PS	1.4-Dx	-4.8084	-2.1722	2.6362	1.3181	-3.4903	3.4903	0.7587	-3.4903	0.6591	4.6211
	THF	-4.8438	-2.2430	2.6008	1.3004	-3.5434	3.5434	0.7690	-3.5434	0.6502	4.8275
	DMSO	-4.5746	-2.7304	1.8443	0.9221	-3.6525	3.6525	1.0844	-3.6525	0.4611	7.2336
4-PS	1.4-Dx	-4.6685	-2.2753	2.3932	1.1966	-3.4719	3.4719	0.8357	-3.4719	0.5983	5.0370
	THF	-4.4408	-2.7687	1.6720	0.8360	-3.6047	3.6047	1.1962	-3.6047	0.4180	7.7716
	DMSO	-4.4707	-2.7361	1.7346	0.8673	-3.6034	3.6034	1.1530	-3.6034	0.4337	7.4855
2-QN	1.4-Dx	-4.4190	-2.0778	2.3412	1.1706	-3.2484	3.2484	0.8543	-3.2484	0.5853	4.5071
	THF	-4.4974	-2.1801	2.3173	1.1586	-3.3387	3.3387	0.8631	-3.3387	0.5793	4.8105
	DMSO	-4.5414	-2.2324	2.3091	1.1545	-3.3869	3.3869	0.8661	-3.3869	0.5773	4.9678
4-QN	1.4-Dx	-4.4024	-2.1208	2.2816	1.1408	-3.2616	3.2616	0.8766	-3.2616	0.5704	4.6625
	THF	-4.4906	-2.2514	2.2392	1.1196	-3.3710	3.3710	0.8932	-3.3710	0.5598	5.0749
	DMSO	-4.5442	-2.3232	2.2209	1.1105	-3.4337	3.4337	0.9005	-3.4337	0.5552	5.3088
2-QO	1.4-Dx	-4.2535	-1.3310	2.9225	1.4612	-2.7923	2.7923	0.6843	-2.7923	0.7306	2.6679
	THF	-4.4220	-2.7810	1.6410	0.8205	-3.6015	3.6015	1.2188	-3.6015	0.4102	7.9041
	DMSO	-4.4220	-2.7810	1.6410	0.8205	-3.6015	3.6015	1.2188	-3.6015	0.4102	7.9041
4-QO	1.4-Dx	-4.5880	-2.2982	2.2898	1.1449	-3.4431	3.4431	0.8734	-3.4431	0.5724	5.1773
	THF	-4.6557	-2.4120	2.2438	1.1219	-3.5339	3.5339	0.8914	-3.5339	0.5609	5.5657
	DMSO	-4.6979	-2.4707	2.2272	1.1136	-3.5843	3.5843	0.8980	-3.5843	0.5568	5.7685
2-QS	1.4-Dx	-4.3469	-2.8321	1.5147	0.7574	-3.5895	3.5895	1.3204	-3.5895	0.3787	8.5062
	THF	-4.4048	-2.8131	1.5917	0.7959	-3.6090	3.6090	1.2565	-3.6090	0.3979	8.1826
	DMSO	-4.4421	-2.8008	1.6413	0.8206	-3.6215	3.6215	1.2186	-3.6215	0.4103	7.9909
4-QS	1.4-Dx	-4.6160	-2.3415	2.2745	1.1373	-3.4787	3.4787	0.8793	-3.4787	0.5686	5.3205
	THF	-4.6680	-2.4522	2.2158	1.1079	-3.5601	3.5601	0.9026	-3.5601	0.5539	5.7201
	DMSO	-4.7055	-2.5099	2.1956	1.0978	-3.6077	3.6077	0.9109	-3.6077	0.5489	5.9281

Table S3. CT parameters for the bright low-lying excited state.

No	Solvent	2-Ar		4-Ar	
		q_{CT} (e)	D_{CT} (Å)	q_{CT} (e)	D_{CT} (Å)
PN	1.4-Dx	0.541	2.651	0.356	1.970
	THF	0.539	2.737	0.351	2.101
	DMSO	0.537	2.862	0.351	2.153
PO	1.4-Dx	0.484	2.109	0.331	1.456
	THF	0.481	2.355	0.333	1.557
	DMSO	0.556	1.654	0.544	2.982
PS	1.4-Dx	0.479	2.062	0.321	1.275
	THF	0.466	2.268	0.542	3.025
	DMSO	0.559	1.667	0.542	3.025
QN	1.4-Dx	0.497	2.141	0.335	1.344
	THF	0.497	2.436	0.328	1.561
	DMSO	0.497	2.585	0.327	1.693
QO	1.4-Dx	0.933	2.773	0.317	1.018
	THF	0.466	1.277	0.315	1.350
	DMSO	0.530	1.550	0.315	1.350
QS	1.4-Dx	0.417	0.924	0.311	0.894
	THF	0.479	1.314	0.307	1.081
	DMSO	0.543	1.576	0.308	1.196

Table S4. Occupancy (e), energy (a.u.) and polarity (%) of natural bond orbitals (NBOs) and hybrids calculated for investigated compounds.

Solvent	NBO orbital	Orbital energy	Ocupancy		Charge	Polarity	NBO hybrid	Atomic orbitals
**2-PN**								
1,4-DX	σ (N – C)	-0.58399	1.96800	N	-0.56070	69.78	$sp^{7.59}$	s(11.65%) p(88.35%)
				I	0.18821	30.22	$sp^{14.56}$	s(6.43%) p(93.57%)
THF	σ (N – C)	-0.58622	1.96828	N	-0.55947	69.31	$sp^{7.63}$	s(11.58%) p(88.42%)
				I	0.17298	30.69	$sp^{14.72}$	s(6.36%) p(93.64%)
DMSO	σ (N – C)	-0.58750	1.96850	N	-0.55916	69.08	$sp^{7.64}$	s(11.57%) p(88.43%)
				I	0.16546	30.92	$sp^{14.73}$	s(6.36%) p(93.64%)
**4-PN**								
1,4-DX	σ (N – C)	-0.58755	1.97008	N	-0.56549	68.64	$sp^{7.18}$	s(12.23%) p(87.77%)
				I	0.12381	31.36	$sp^{15.56}$	s(6.04%) p(93.96%)
THF	σ (N – C)	-0.58924	1.96987	N	-0.56422	68.04	$sp^{7.25}$	s(12.13%) p(87.87%)
				I	0.10810	31.96	$sp^{15.53}$	s(6.05%) p(93.95%)
DMSO	σ (N – C)	-0.58924	1.96987	N	-0.56422	68.04	$sp^{7.29}$	s(12.13%) p(87.87%)
				I	0.10810	31.96	$sp^{15.53}$	s(6.05%) p(93.95%)

**2-PO**								
1,4-DX	σ (N – C)	-0.59020	1.96715	N	-0.56011	69.95	$sp^{7.53}$	s(11.73%) p(88.27%)
				I	0.19897	30.05	$sp^{15.11}$	s(6.21%) p(93.79%)
THF	σ (N – C)	-0.59158	1.96748	N	-0.55891	69.55	$sp^{7.55}$	s(11.70%) p(88.30%)
				I	0.18908	30.45	$sp^{15.18}$	s(6.18%) p(93.82%)
DMSO	σ (N – C)	-0.61272	1.97118	N	-0.55661	71.30	$sp^{7.03}$	s(12.46%) p(87.54%)
				I	-0.40873	28.70	$sp^{15.05}$	s(6.23%) p(93.77%)
**4-PO**								
1,4-DX	σ (N – C)	-0.59341	1.96919	N	-0.56377	68.76	$sp^{7.53}$	s(12.31%) p(87.69%)
				I	0.14266	31.24	$sp^{16.06}$	s(5.86%) p(94.14%)
THF	σ (N – C)	-0.59442	1.96842	N	-0.56251	68.31	$sp^{7.21}$	s(12.19%) p(87.81%)
				I	0.13469	31.69	$sp^{16.08}$	s(5.86%) p(94.14%)
DMSO	σ (N – C)	-0.61022	1.96923	N	-0.55932	71.07	$sp^{7.01}$	s(12.48%) p(87.52%)
				I	0.34260	28.93	$sp^{16.27}$	s(5.79%) p(94.21%)
**2-PS**								
1,4-DX	σ (N – C)	-0.59130	1.96692	N	-0.55992	70.04	$sp^{7.53}$	s(11.72%) p(88.28%)
				I	0.20541	29.96	$sp^{15.12}$	s(6.20%) p(93.80%)
THF	σ (N – C)	-0.59238	1.96718	N	-0.55878	69.61	$sp^{7.55}$	s(11.70%) p(88.30%)
				I	0.19308	30.39	$sp^{15.25}$	s(6.15%) p(93.85%)
DMSO	σ (N – C)	-0.61333	1.97107	N	-0.55698	71.34	$sp^{7.02}$	s(12.47%) p(87.53%)
				I	0.39807	28.66	$sp^{15.11}$	s(6.21%) p(93.79%)
**4-PS**								
1,4-DX	σ (N – C)	-0.59465	1.96892	N	-0.56377	68.83	$sp^{7.02}$	s(12.33%) p(87.67%)
				I	0.14652	31.17	$sp^{15.11}$	s(5.84%) p(94.16%)
THF	σ (N – C)	-0.61116	1.96931	N	-0.55922	70.92	$sp^{7.02}$	s(12.44%) p(87.56%)
				I	0.33189	29.08	$sp^{15.11}$	s(5.84%) p(94.16%)
DMSO	σ (N – C)	-0.61116	1.96931	N	-0.55922	70.92	$sp^{7.02}$	s(12.44%) p(87.56%)
				I	0.33189	29.08	$sp^{15.11}$	s(5.84%) p(94.16%)
**2-QN**								
1,4-DX	σ (N – C)	-0.58255	1.96004	N	-0.55446	70.70	$sp^{7.93}$	s(11.19%) p(88.81%)
				I	0.21396	29.30	$sp^{14.39}$	s(6.50%) p(93.50%)
THF	σ (N – C)	-0.58506	1.96054	N	-0.55291	70.32	$sp^{7.98}$	s(11.13%) p(88.87%)
				I	0.20319	29.68	$sp^{14.52}$	s(6.44%) p(93.56%)
DMSO	σ (N – C)	-0.58649	1.96077	N	-0.55232	70.15	$sp^{8.00}$	S(11.11%) p(88.89%)
				I	0.19904	29.85	$sp^{14.59}$	s(6.42%) p(93.58%)

**4-QN**								
1,4-DX	σ (N – C)	-0.58514	1.96216	N	-0.56044	70.18	$sp^{7.47}$	s(11.81%) p(88.19%)
				I	0.16664	29.8	$sp^{15.10}$	s(6.21%) p(93.79%)
THF	σ (N – C)	-0.58814	1.96238	N	-0.55920	69.87	$sp^{7.52}$	s(11.73%) p(88.27%)
				I	0.16325	30.13	$sp^{14.91}$	s(6.29%) p(93.71%)
DMSO	σ (N – C)	-0.58990	1.96248	N	-0.55855	69.73	$sp^{7.55}$	s(11.70%) p(88.30%)
				I	0.16245	30.27	$sp^{14.82}$	s(6.32%) p(93.68%)
**2-QO**								
1,4-DX	σ (N – C)	-0.61514	1.97172	N	-0.55112	71.08	$sp^{7.20}$	s(12.20%) p(87.80%)
				I	0.36834	28.92	$sp^{14.03}$	s(6.65%) p(93.35%)
THF	σ (N – C)	-0.61715	1.97172	N	-0.55149	71.51	$sp^{7.10}$	s(12.34%) p(87.66%)
				I	0.40333	28.49	$sp^{14.42}$	s(6.48%) p(93.52%)
DMSO	σ (N – C)	-0.61817	1.97171	N	-0.55211	71.78	$sp^{8.00}$	s(12.43%) p(87.57%)
				I	0.42178	28.22	$sp^{14.67}$	s(6.38%) p(93.62%)
**4-QO**								
1,4-DX	σ (N – C)	-0.59142	1.96207	N	-0.55934	70.43	$sp^{7.40}$	s(11.91%) p(88.09%)
				I	0.19095	29.57	$sp^{15.33}$	s(6.12%) p(93.88%)
THF	σ (N – C)	-0.59379	1.96247	N	-0.55723	69.9	$Sp^{7.44}$	s(11.84%) p(88.16%)
				I	0.18515	30.03	$sp^{15.50}$	s(6.06%) p(93.94%)
DMSO	σ (N – C)	-0.59519	1.96254	N	-0.55638	69.80	$sp^{7.46}$	s(11.82%) p(88.18%)
				I	0.18444	30.20	$sp^{15.57}$	s(6.03%) p(93.97%)
**2-QS**								
1,4-DX	σ (N – C)	-0.61685	1.97173	N	-0.55090	71.18	$sp^{7.17}$	s(12.24%) p(87.76%)
				I	0.37752	28.82	$sp^{14.07}$	s(6.64%) p(93.36%)
THF	σ (N – C)	-0.61828	1.97167	N	-0.55168	71.59	$sp^{7.09}$	s(12.37%) p(87.63%)
				I	0.40878	28.41	$sp^{14.47}$	s(6.47%) p(93.53%)
DMSO	σ (N – C)	-0.61906	1.97163	N	-0.55243	71.84	$sp^{7.04}$	s(12.45%) p(87.55%)
				I	0.42582	28.1	$sp^{14.70}$	s(6.37%) p(93.63%)
**4-QS**								
1,4-DX	σ (N – C)	-0.59263	1.96197	N	-0.55932	70.49	$sp^{7.39}$	s(11.92%) p(88.08%)
				I	0.19494	29.51	$sp^{15.39}$	s(6.10%) p(93.90%)
THF	σ (N – C)	-0.59474	1.96230	N	-0.55719	70.02	$sp^{7.43}$	s(11.86%) p(88.14%)
				I	0.18901	29.98	$sp^{15.59}$	s(6.03%) p(93.97%)
DMSO	σ (N – C)	-0.59607	1.96235	N	-0.55631	69.85	$sp^{7.45}$	S(11.83%) p(88.17%)
				I	0.18853	30.15	$sp^{15.69}$	s(5.99%) p(94.01%)

Table S5. Solvation free energy values.

No	ΔG_{solv} (kcal/mol)		
	1,4-Dx	THF	DMSO
2-PN	-8.37	-20.54	-12.66
4-PN	-8.89	-21.14	-12.31
2-PO	-7.38	-18.90	-10.35
4-PO	-7.92	-16.89	-10.95
2-PS	-7.65	-19.40	-11.44
4-PS	-8.29	-13.45	-11.75
2-QN	-9.99	-22.98	-14.06
4-QN	-9.98	-23.43	-14.14
2-QO	-9.70	-19.23	-11.23
4-QO	-9.76	-19.88	-11.38
2-QS	-9.12	-20.77	-12.04
4-QS	-9.22	-21.77	-12.97

Table S6. Theoretically determined absorption band maxima.

No	Solvent	2-Ar		4-Ar	
		λ_{ABS} (nm)	f	λ_{ABS} (nm)	f
PN	1,4-Dx	423.99	0.73	438.70	0.86
	THF	430.32	0.71	442.22	0.90
	DMSO	430.99	0.76	440.60	0.89
PO	1,4-Dx	375.27	0.72	380.07	0.80
	THF	374.02	0.71	380.52	0.82
	DMSO	378.14	0.73	385.85	0.82
PS	1,4-Dx	368.56	0.75	379.07	0.79
	THF	377.69	0.76	381.24	0.76
	DMSO	380.47	0.70	385.79	0.76
QN	1,4-Dx	488.86	0.64	500.86	0.69
	THF	490.39	0.65	503.24	0.66
	DMSO	491.85	0.65	505.42	0.67
QO	1,4-Dx	416.62	0.61	418.38	0.68
	THF	421.38	0.65	425.33	0.62
	DMSO	422.57	0.63	430.32	0.63
QS	1,4-Dx	416.67	0.64	421.98	0.63
	THF	418.47	0.70	425.31	0.65
	DMSO	419.43	0.67	427.71	0.64

Table S7. Theoretical values of dipole moments in the ground (μ_{GS}) and excited states (μ_{CT}).

No	Solvent	2-Ar		4-Ar	
		μ_{GS} (D)	μ_{CT} (D)	μ_{GS} (D)	μ_{CT} (D)
PN	1,4-Dx	2.64	8.39	4.84	15.77
	THF	2.67	10.83	5.45	16.62
	DMSO	2.63	11.53	5.93	17.47
PO	1,4-Dx	3.87	9.66	5.43	15.81
	THF	4.48	10.27	6.26	17.5
	DMSO	5.88	11.44	8.75	18.66
PS	1,4-Dx	3.98	9.98	6.08	17.53
	THF	4.73	10.47	8.91	19.50
	DMSO	6.45	12.85	9.81	19.62
QN	1,4-Dx	2.36	10.26	5.13	15.73
	THF	2.44	10.35	5.99	16.98
	DMSO	2.45	10.53	6.43	17.33
QO	1,4-Dx	5.76	12.19	5.26	17.47
	THF	6.27	13.08	6.23	18.91
	DMSO	6.96	13.96	6.74	19.83
QS	1,4-Dx	5.66	13.66	5.98	19.64
	THF	7.03	13.48	7.06	20.72
	DMSO	7.77	13.57	7.64	21.35

References

1. Koelsch, C.F. The Condensation of α-Picoline Methiodide with Aromatic Aldehydes. *J. Am. Chem. Soc.* **1944**, *66*, 2126–2126, doi:10.1021/ja01240a505.
2. Ballistreri, F.P.; Barresi, V.; Consiglio, G.; Fortuna, C.G.; Longo, M.L.; Musumarra, G. Synthesis, spectroscopic characterization and in vitro antitumor activity of new trans 1-heteroaryl-2-(1-methylpyridinium-2-yl) ethylenes. *ARKIVOC* **2003**, *(i)*, 105–117.
3. Phillips, A.P. Condensation of aromatic aldehydes with γ-picoline methiodide. *J. Org. Chem.* **1949**, *14*, 302–305, doi:10.1021/jo01154a017.
4. Acheson, R.M.; Harrison, D.R. The synthesis, spectra, and reactions of some S-alkylthiophenium salts. *Journal of the Chemical Society C: Organic* **1970**, 1764–1784, doi:10.1039/J39700001764.
5. Bradamante, S.; Facchetti, A.; Pagani, G.A. Heterocycles as donor and acceptor units in push–pull conjugated molecules. Part 1. *J. Phys. Org. Chem.* **1997**, *10*, 514–524, doi:10.1002/(SICI)1099-1395(199707)10:7<514::AID-POC897>3.0.CO;2-J.
6. Xie, X.; Zuffo, M.; Teulade-Fichou, M.-P.; Granzhan, A. Identification of optimal fluorescent probes for G-quadruplex nucleic acids through systematic exploration of mono- and distyryl dye libraries. *Beilstein Journal of Organic Chemistry* **2019**, *15*, 1872–1889, doi:10.3762/bjoc.15.183.
7. Werner, L.F. The methiodides of the condensation products of some cyclic aldehydes with quinaldine and alpha picoline, and their possible value as indicators in acidimetry. *J. Am. Chem. Soc.* **1920**, *42*, 2309–2314, doi:10.1021/ja01456a024.

8. Cavallito, C.J.; Yun, H.S.; Kaplan, T.; Smith, J.C.; Foldes, F.F. Choline acetyltransferase inhibitors. Dimensional and substituent effects among styrylpyridine analogs. *J. Med. Chem.* **1970**, *13*, 221–224, doi:10.1021/jm00296a013.

9. Abbotto, A.; Beverina, L.; Bozio, R.; Bradamante, S.; Ferrante, C.; Pagani, G.A.; Signorini, R. Push–Pull Organic Chromophores for Frequency-Upconverted Lasing. *Adv. Mater.* **2000**, *12*, 1963–1967, doi:10.1002/1521-4095(200012)12:24<1963::AID-ADMA1963>3.0.CO;2-S.
